# Illuminating the daily life experiences of adolescents with and without ADHD: protocol for an ecological momentary assessment study

**DOI:** 10.1136/bmjopen-2023-077222

**Published:** 2023-09-29

**Authors:** Aja Murray, Lydia Speyer, Melissa Thye, Tracy Stewart, Ingrid Obsuth, Jennifer Kane, Katie Whyte, John Devaney, Luis Augusto Rohde, Anastasia Ushakova, Sinead Rhodes

**Affiliations:** 1 Department of Psychology, University of Edinburgh, Edinburgh, UK; 2 Department of Psychology, Lancaster University, Edinburgh, UK; 3 Moray House of Education and Sport, University of Edinburgh, Edinburgh, UK; 4 Clinical and Health Psychology Department, University of Edinburgh, Edinburgh, UK; 5 Department of Psychology, St Andrew’s University, St Andrews, UK; 6 School of Social and Political Science, University of Edinburgh, Edinburgh, UK; 7 ADHD Outpatient Program and Developmental Psychiatry Program, Hospital de Clinicas de Porto Alegre, Universidade Federal do Rio Grande do Sul, Porto Alegre, Brazil; 8 National Institute of Developmental Psychiatry & National Center for Research and Innovation in Mental Health, Sao Paolo, Brazil; 9 UniEduk, Brazil, Brazil; 10 Centre for Computing, Health Informatics and Statistics, Lancaster University, Lancaster, UK; 11 Centre for Clinical Brain Sciences, University of Edinburgh, Edinburgh, UK

**Keywords:** Child & adolescent psychiatry, Depression & mood disorders, Anxiety disorders, Adolescent

## Abstract

**Introduction:**

Adolescents with attention-deficit/hyperactivity disorder (ADHD) are at elevated risk of a range of difficulties, among which emotion regulation, peer and co-occurring mental health problems are prominent challenges. To better support adolescents with ADHD, ecologically valid interventions that can be embedded in daily life to target the most proximal antecedents of these challenges are needed. Ecological momentary assessment (EMA) designs are ideally suited to meeting this need.

**Methods and analyses:**

In the mental health in the moment ADHD study, we will use an EMA design to capture the daily life experiences of approximately 120 adolescents aged 11–14 years with a clinical diagnosis of ADHD and the same number of age-matched and gender-matched peers without a diagnosis of ADHD. We will combine this with comprehensive information gathered from online surveys. Analysing the data using techniques such as dynamic structural equation modelling, we will examine, among other research questions, the role of emotion regulation and peer problems in mediating the links between characteristics of ADHD and commonly co-occurring outcomes such as anxiety, depression and conduct problems. The results can help inform interventions to support improved peer functioning and emotion regulation for adolescents with ADHD.

**Ethics and dissemination:**

This study received a favourable ethical opinion through the National Health Service ethical review board and the University of Edinburgh PPLS Research Ethics panel. The results will be disseminated through journal publications, conferences and seminar presentations and to relevant stakeholders, such as those with ADHD, their families and clinicians.

**STRENGTHS AND LIMITATIONS OF THIS STUDY**

- This study will use an ecological momentary assessment (EMA) design to collect high temporal resolution, ecologically value daily life experience data from young people with attention-deficit/hyperactivity disorder.
- It will use cutting edge statistical analysis techniques to disentangle different aspects of emotional functioning in daily life to identify key intervention targets.
- A limitation is that data will be collected only over a short period, therefore, developmental changes will not be captured.
- Further, EMA methods may be vulnerable to low response rates, careless responding or dropout and the reliance on self-reports can be associated with reporting biases.

## Introduction

Attention-deficit/hyperactivity disorder (ADHD) symptoms may increase the risk of experiencing a range of difficulties in adolescence^1–3^ and prominent among these are inter-related challenges in emotion regulation and peer relationships.^4^ These have been suggested to contribute to the development of co-occurring mental health conditions such as anxiety, depression and conduct problems^5–7^; outcomes identified as a high priority for further research in consultations with neurodivergent experts-by-experience (Embracing Complexity, 2022). However, critical reviews^8–10^ have suggested a strong need for better interventions to support neurodivergent adolescents facing these challenges. In particular, current interventions have been criticised for a lack of generalisability to real-life contexts, for not directly targeting the interconnections between emotion dysregulation and peer problems, and for a lack of tailoring to the specific needs of adolescents (eg, Morris *et al*^9^). The goal of the present study is to provide evidence that helps address this gap, using a data collection technique that gathers rich ‘in-the-moment’ data about the daily life experiences and functioning of adolescents with and without ADHD.

Difficulties with emotion regulation among children and adolescents with ADHD are well documented.^4 11^ Emotion regulation can be defined as the set of processes by which individuals evaluate, inhibit or modify their emotional reactions in order to show a socially appropriate behaviour and/or achieve some goal.^12^ Emotional dysregulation may manifest in a number of ways, including in impaired emotion recognition or understanding, a lack of empathy/callous-unemotional traits and emotional lability or reactivity. In comparison with neurotypical peers, youth with ADHD show greater emotional lability or reactivity^13^ characterised by more intense, undercontrolled or rapidly shifting emotions than are appropriate for developmental stage and context and which consequently impacts functioning.^14^ This may include dysregulation of positive emotions, leading to excessive excitability.^1^

Emotion dysregulation often exists alongside and has been proposed to contribute to peer difficulties such as bullying victimisation and perpetration, peer aggression, rejection, dislike and conflict among adolescents with ADHD.^1 15^ As a result of emotion regulation difficulties, adolescents with ADHD may react more strongly and more aggressively to provocations, making them targets for victimisation and placing them at higher risk of showing reactive aggression.^16^ They may become overexcited and experience resultant difficulties in attending to what is required of them in a given social situation.^1^ A role for rejection sensitivity, defined as the tendency to anxiously anticipate, overperceive and react intensely to social rejection (potentially arising as a result of repeated experiences of rejection), has also been proposed.^17 18^ Intense and dysregulated reactions to perceived rejection (eg, excessive reassurance seeking or anger) may be experienced as aversive to peers and result in a ‘self-fulfilling prophecy’ of peer rejection.^18^ Overall, it has been noted that some youth with ADHD, as a result of emotion regulation difficulties, may be perceived as intense, disruptive or overly exuberant, potentially leading to greater exposure to dislike, rejection and victimisation.^15^ Indeed, given their interconnectedness, the self-regulation of emotions, alongside behavioural inhibition, is thought to be one of the most important aspects of interventions to support better social functioning in adolescence.^9^

Emotion dysregulation and peer problems are also thought to contribute to the development of co-occurring mental health and behavioural challenges associated with ADHD symptoms. For example, previous research in children and adolescents has found that peer issues and emotion regulation mediate the associations between ADHD symptoms and internalising problems such as anxiety and depression^5 6 19 20^ and externalising problems such as aggression, oppositional and conduct problem behaviours.^21 22^

However, the daily life mechanisms underpinning these links and—correspondingly—the most proximal targets for intervention remain poorly understood, particularly in the critical period of adolescence. Ecological momentary assessment (EMA) has proven valuable in child and adolescent mental health research for capturing symptoms and related experiences in near real time and in the flow of daily life and can thus inform more ecologically embedded intervention approaches.^23^ It is particularly valuable for capturing concepts such as emotional (dys)regulation and social interactions in daily life contexts.^6^ For example, EMA data can be used to provide measures of different aspects of emotional functioning in daily life, such as overall levels of negative affect, affective lability, emotional inertia and emotional reactivity to stressors or provocations.^24 25^

There have, however, been only a handful EMA studies of young people with ADHD during the critical period of adolescence.^26 27^ Reviewing the literature on EMA studies of ADHD populations, a recent review^26^ identified less than 10 studies conducted in adolescents, most of which had small sample sizes and none of which addressed the links between emotion regulation, peer issues and co-occurring mental health and behavioural outcomes. Only one previous EMA study has examined the impact of emotion regulation on peer issues and mental health outcomes in a sample of adolescents with ADHD.^1^ Using a daily diary design, they found that adolescents with ADHD tend to experience more negative emotion, less positive emotion and more emotional variability. Variability in some emotions was associated with outcomes such as social functioning and externalising problems.

These initial findings point to the value of further exploring, using EMA, the daily life manifestation and impacts of emotion regulation and peer interactions in adolescents with ADHD. For example, as well as seeking to extend these findings to examine a wider range of emotion regulation and peer issues, it will be valuable to examine factors that may impact these domains, their interconnections, and their links to mental health and behavioural outcomes. This will provide illumination on further modifiable targets for intervention, such as physical activity, improved sleep, secure attachment to caregivers and positive teacher–student relationships.^25 28–30^ Further, strengths associated with ADHD characteristics, such as a high level of energy and drive, the ability to focus intensely on tasks of interest, and positive temperamental factors^31^ will be explored as these too could potentially be leveraged as part of more effective interventions.

We will thus use an EMA design to illuminate how, in the flow of their daily lives, adolescents with ADHD may experience difficulties with emotion regulation and peer problems, how these may contribute to co-occurring mental health and behavioural problems. We will also examine other potential modifiable targets for intervention that may impact emotion regulation, peer problems, mental health and behaviour as well as their links among adolescents with ADHD (eg, ADHD strengths, relationships, sleep, physical activity). Core research questions are:

- Which aspects of emotion regulation differ between adolescents with and without ADHD, for example, negative or positive emotional lability, emotional reactivity, emotional inertia, emotion regulation strategy use and emotion regulation strategy success?
- Which aspects of emotion regulation mediate the association between ADHD diagnosis/symptoms and peer problems in daily life?
- Do emotion regulation and peer issues mediate the links between ADHD diagnosis/symptoms and anxiety, depression and conduct problems?

Exploratory analyses may be used to answer a range of additional questions such as exploration of moderation of the links between ADHD diagnosis/symptoms and outcomes to identify other potential intervention targets.

## Methods and analyses

### Participants

Participants are expected to include approximately 120 adolescents aged 11–14 years with a clinical diagnosis of ADHD and 120 matched controls. The clinical sample will be recruited via Child and Adolescent Mental Health Services (CAMHS) in two primary sites in the UK (one in Scotland and one in England) and community partners. In CAMHS, the recruitment procedure will vary from service to service; however, in general participants will be recruited at medication clinics and through ongoing casework.

A matched control (on age±3 months, gender, and if possible, city and socioeconomic status based on Scottish Index of Multiple Deprivation in Scotland and Index of Multiple Deprivation in England) will be selected for each participant with a clinical diagnosis of ADHD from a larger pool of participants taking part in adolescent mental health studies (co-)led by the first author. To maximise relevance to community-ascertained samples, minor modifications to the protocol will be made, detailed in the Measures section.

#### Inclusion and exclusion criteria

Inclusion criteria for the clinical sample will be: aged 11–14 years, a formal clinical diagnosis of ADHD (parent-reported in the case of the youth recruited via the community), the ability to provide informed assent and parental informed consent to participate and the ability to independently complete online surveys and EMA protocols. There will be no explicit exclusion criteria based on co-occurring neurodevelopmental conditions as ADHD commonly co-occurs with other neurodevelopmental conditions^32^ and excluding youth with these conditions would undermine the representativeness of the sample. It would also preclude the opportunity to illuminate how ADHD characteristics may interact with those of other neurodevelopmental conditions.^33^ For similar reasons and given our goal of illuminating candidate mechanisms by which ADHD characteristics may impact mental health and behavioural outcomes, we will also not impose any exclusion criteria related to any other co-occurring psychiatric issues (eg, anxiety, depression, conduct disorder). However, we acknowledge that the requirement to be able to complete the online surveys and EMA surveys independently may result in the exclusion of some young people (eg, those with a moderate to severe intellectual disability). Adaptation of the protocol to better include these groups will be the subject of future research. Inclusion and exclusion criteria for the control sample will be identical, except that a formal clinical diagnosis of ADHD will be an exclusion criterion for controls.

### Patient and public involvement

Adolescents with ADHD and parents of adolescents with ADHD are the most important stakeholders in this project. Their views have informed the project design, including the current protocol and will continue to inform the project and the dissemination of its outputs. Young person and parent advisors were recruited via the ADHD charities and organisations with whom our team has collaborative links. Examples of major changes made to the protocol based on their input included extending the inclusion criteria to include lower ages, reducing the length of the surveys, including more positively worded and fewer negatively worded items, removing elements of the project that would have involved location tracking and wearable technology, adding a measure of rejection sensitivity and embedding more fun and educational elements into the study, such as providing ‘ADHD facts’ at the end of each completed EMA survey.

### Data collection

**Figure 1:**
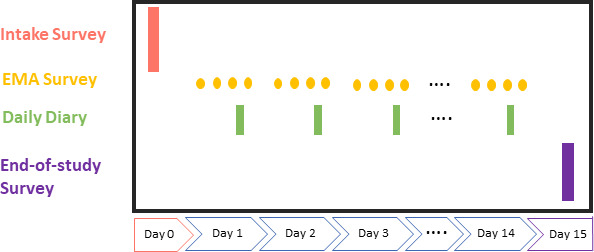
Summary of data collection schedule. EMA, ecological momentary assessment.

Data collection is projected to begin in August 2023 and be completed in 12 months. Figure 1 summarises the study flow. Informed parental consent and adolescent assent will be collected, then both adolescents and their parents will be invited to complete an online ‘intake’ survey. This reflects the importance of obtaining a ‘multi-informant’ perspective.^34–36^

On completion of the online intake survey, adolescent participants will initiate the *EMA surveys*. They will be provided with instructions on how to take part and offered on-boarding support via a video call with study staff. Participants will use their own smartphones where possible; however, to avoid selection biases and promote inclusion, study smartphones will be available. An email helpline will also be available for participants to receive help on any issues they encounter.

EMA data collection will take place over a 2-week period and involve five measures per day, all administered via a smartphone application. Four of these measures will be delivered at quasirandom intervals (random within four blocks) and include identical measures of momentary experiences. The fifth will be a brief ‘daily diary’ that captures concepts that vary over slightly longer timescales (eg, sleep, medication use, physical activity). All measures will be designed to take less than 2 min as long questionnaires are associated with increased burden and lower compliance and our young person advisory group (YPAG)/parent advisory group (PAG) emphasised the importance of minimising burden.^37^

At the end of the 2-week EMA period, participants will complete an online *end-of study* survey. Here, they will complete repeat measures of a subset of the questions administered in the original survey, as well as a brief set of questions capturing their participation experiences. Full questionnaires are provided in online supplemental material.

10.1136/bmjopen-2023-077222.supp1 Supplementary data

### Measures

#### Adolescent measures

The measures that adolescents will complete over the course of the study are presented in table 1. Full descriptions of the measures are provided in online supplemental material.

**Table 1:** Measures for adolescent surveys

Phase	Measures	Sources	items, n
Intake			
	Demographics	Gender, date of birth, ethnicity, postcode	4
	Internalising problems	Revised Child Anxiety and Depression Scale (RCADS-11)^57^	11
	ADHD strengths*	Newly developed measure	8
	Emotion dysregulation	Difficulties in Emotion Regulation Scale-16^58^	16
	Emotion regulation strategy use	Emotion Regulation Questionnaire adapted for Children and Adolescents^59^	10
	Peer problems and friendships	Social Relationships Questionnaire^60 61^	11
	Attachment security	Inventory of Parent and Peer Attachment-10^62^	10
	Quality of teacher–student relationships	Measure used in prior longitudinal study^63^	3
	Self-esteem	Rosenberg Self-Esteem Questionnaire^64^	10
	Autistic traits	Autism Symptom SElf-ReporT^65 66^	7
	Rejection sensitivity	Child Rejection Sensitivity Questionnaire^67 68^	6
EMA			
	ADHD symptoms	Adapted from Diagnostic and Statistical Manual (DSM) criteria and prior ADHD EMA studies^3^	3
	Medication use*	Has medication been taken since last assessment	1
	Emotions	Adapted from Positive Affect Negative Affect Schedule expanded^69^	6
	Emotion regulation strategies	Adapted version of the Emotion Regulation Questionnaire^70^	2
	Peer interactions	Newly developed measure	4
Daily diary			
	Medication use*	Whether and when medication was taken in the last 24 hours	2
	Physical activity	Adapted from an open EMA item pool^71^	3
	Sleep	Abbreviated version of the Consensus Sleep Diary core items^72^	7
End			
	Internalising problems	RCADS-11^57^	11
	Participation experiences	EMA and daily diary enjoyment, burden and impact on routine; suggestions	4

*Not completed by the control sample.

ADHD, attention-deficit/hyperactivity disorder; EMA, ecological momentary assessment.

#### Parent measures

The measures that parents will complete over the course of the study are presented in table 2. Full descriptions of the measures are provided in online supplemental material.

**Table 2:** Measures for parent surveys

Phase	Measures	Sources	Items, n
Intake			
	Demographics	Respondent gender, ethnicity, education, postcode, free school meals	5
	Child’s ADHD or other diagnoses	Diagnosis source, diagnosis age, other diagnoses	3
	Child’s ADHD medication use	Medication Adherence Report Scale^73^	5
	Child’s ADHD strengths*	Newly developed measure	8
	Child’s ADHD symptoms	Disruptive Behavior Disorder Rating Scale (DBD)^74^	18
	Child’s internalising problems	Revised Child Anxiety and Depression Scale (RCADS-11)^57^	11
	Child’s peer conflict and friendships	Social Relationships Questionnaire^60 61^	11
End			
	Child’s ADHD, conduct and oppositional symptoms	DBD^74^	42
	Child’s internalising problems	RCADS-11^57^	11
	Child’s participation experiences	Study enjoyment and likelihood to participate again; suggestions	3

*Not completed by parents of the control sample.

ADHD, attention-deficit/hyperactivity disorder.

### Piloting

Prior to the main data collection, the protocol will be piloted in a sample of n=10 adolescents (5 with and five without ADHD). Further modifications may be made to the protocol after piloting.

### Statistical procedure

Data will be analysed using techniques such as dynamic structural equation modelling (DSEM).^38–40^ For example, using two-level DSEM with participant random effects, it will be possible to derive individual-level indices of the mean levels, variability and covariation of different concepts measured in the EMA. For example, we can derive indices of emotion regulation such as: variability in emotional state (lability), strength of relations between prior and subsequent emotional state (inertia), strength of relations between an event (eg, a peer conflict) and emotional state (reactivity) and examine their links to peer, mental health and behavioural issues, including as mediators of the links between ADHD symptoms and these outcomes. They can be studied alongside emotion regulation strategy use, resulting emotional states, and differences in these between adolescents with and without ADHD. Specific confirmatory analyses will be preregistered and suitable multiple comparisons correction approaches selected as relevant.

### Sample size justification

Our sample size (n=120 adolescents with ADHD and n=120 without) draws on several sample size determination approaches. It is partly based on a resource constraint approach, that is, we will maximise the sample size attainable within the resources available.^41^ It is informed by Monte Carlo simulation studies using DSEM population models with varying effect sizes for the within-person and between-person structures. These have suggested that a minimum of 50 respondents with 10 measurement points are needed to avoid small sample bias for random effects and to provide adequate coverage for effect sizes at the within-person and between-person level that are likely to be of clinical importance.^42 43^ This is supplemented by our own simulations using population parameters derived from data gathered using similar designs in previous studies suggest that 100 respondents (50 per group) measured 50 times provides adequate coverage for realistic parameter estimates, for example: https://osf.io/pvrma/ includes tailored simulations for this study. Taken together, our choice of n=120 per group balances feasibility with statistical precision and includes buffer for missing data and attrition.

### Ethics and dissemination

Ethical review for the collection of the data from the clinically ascertained sample will follow a three-stage process in which sponsorship will be sought from ACCORD, followed by ethical review by the National Health Service ethical review board, and finally, research and development approval. Ethical review for the collection of data based on community and school recruitment will be by the University of Edinburgh PPLS Research Ethics panel. The results will be disseminated through journal publications, conferences and seminar presentations and to relevant stakeholders, such as those with ADHD, their families and clinicians.

## Discussion

The current protocol describes the planned design for a combined traditional and EMA survey study^44^ of the daily life manifestation and impacts of emotion regulation and peer issues among adolescents with and without ADHD. Building on previous evidence that emotion regulation and peer problems are common and interconnected among youth with ADHD,^1 15^ we will seek to illuminate the most proximal targets in the daily lives of these youths to leverage in interventions. Adolescence is a critical transition period for emotion regulation and social development that may be particularly challenging for youth with ADHD.^15^ Reflecting the importance of involving those most affected by the research in mental health and neurodevelopmental research,^45^ the project has been and will continue to be informed by two panels of experts by experience, namely adolescents with ADHD and parents of adolescents with ADHD.

The study is motivated by a need for new data to inform more ecologically valid interventions that can provide effective improvements to adolescents’ emotional and social functioning in their daily lives.^9^ Though new, more integrative intervention approaches continue to be developed,^46^ the lack of attempts to address emotion regulation and social skills together and a lack of generalisability to real-life functioning are two major critiques of existing supports for adolescents with ADHD.^9^ By gathering information about how emotion regulation and (potential) peer problems manifest, how they are linked and how they contribute to the development of co-occurring mental health or behavioural concerns, our findings can inform improvements to interventions to enhance their ecological validity.

Our findings may be particularly valuable for informing smartphone-based interventions.^47^ These show strong promise for adolescents with ADHD because smartphone use is highly embedded within the daily routines of young people, can allow the regular practice of skills within ecological context and can be responsive to incoming data that, for example, may indicate high moments of risk for adverse outcomes (in ‘ecological momentary interventions’). Their scale-able and easily accessible format can also help make interventions more widely and more quickly available to the population of youth who may benefit from them. However, recent reviews of smartphone applications for ADHD have suggested that very few available applications are based on a robust evidence base.^47^ Further, mental health smartphone-based applications have been shown to be associated with low compliance^48^ and are frequently abandoned after only a small number of uses.^49^ Our study can help to gain insights into the factors that can promote better engagement with smartphone-delivered interventions. This will be particularly important for adolescents with ADHD, who may find sustained engagement with interventions difficult, necessitating tailored protocols that, for example, provide immediate and high value rewards for compliance.

Our findings can also help accelerate progress in the application of EMA methodologies in populations of adolescents with ADHD. To date, despite their promise, very few such studies have been conducted.^26 27^ By providing a template for such research (which we will make openly available in the form of openly accessible study materials, including measures and analysis code), and documenting challenges, solutions and benefits, this study can facilitate wider application of this technique.

### Limitations

Though a large body of research has investigated optimal design features for EMA in general,^37 50–52^ the relative novelty of the approach in adolescents with ADHD (see, eg, Breaux *et al* and Babinski and Welkie^1 53^ for notable exceptions) means that there is little past research to guide the selection of measures, sampling frequency and schedule, incentives, strategies to promote engagement and other design features. More broadly, there remains a lack of attention paid to the development and validation of measures for EMA^54^ and this means that there is little psychometric evidence underpinning available EMA measures of core constructs for the study such as emotion regulation and peer problems.^55^ Our study will thus help contribute to the development and validation of robust EMA measures but will not benefit from a strong pre-existing evidence base to guide which measures are likely to show the best psychometric properties. Finally, though EMA can overcome some of the reporting difficulties associated with traditional surveys (eg, reporting biases and accuracies due to their reliance on retrospective recall), they are not invulnerable to self-report biases. For example, previous research has suggested that some adolescents with ADHD may be vulnerable to positive illusory biases that may result in a lack of awareness of difficulty and thus overly optimistic reporting of functioning.^56^

## Supplementary Material

Reviewer comments

Author's manuscript
