# Supplementary material for: Illuminating the daily life experiences of adolescents with and without ADHD: protocol for an ecological momentary assessment study
Source: BMJ Open. 2023 Sep 29;13(9):e077222. doi: 10.1136/bmjopen-2023-077222 (PMC10546102; doi:10.1136/bmjopen-2023-077222)
Supplement: Supplementary data [file bmjopen-2023-077222supp001.pdf]

Supplementary Materials

S1: Description of Measures

Adolescents intake survey

Gender will be measured using an item with options ‘male’, ‘female’, ‘non-binary’, ‘gender fluid’, ‘questioning’, ‘prefer to self-describe’ and ‘prefer not to say’.

Date of birth will be collected and used to derive age at time of assessment.

Ethnicity will be measured using a set of items designed to be inclusive of different ethnic identities, with the additional options of “Prefer not to say” and “Not listed, please specify.”

Socioeconomic status (SES) will be estimated from respondent’s home postcodes and used to derive the (Scottish) index of multiple deprivation [(S)IMD].

Internalising problems, will be measured using an 11-item (6 anxiety items; 5 depression items) self-report *Revised Child Anxiety and Depression* Scale (38) . Previous research has suggest that the scale scores have favourable psychometric properties in adolescents (38)

ADHD strengths will be measured using a newly developed 8-item measure produced by the team in consultation with the youth and parent advisory groups. Items were written for strengths associated with ADHD that included hyperfocus, creativity, energy, interests, and problem-solving. While previous work has identified perceived strengths associated with ADHD (31), no suitable validated measure of ADHD strengths in this age group currently exists. Full details of the scale development and validation will be documented in future publications. This will not be completed by the control sample.

Emotion dysregulation will be measured using *Difficulties in Emotion Regulation Scale* (39)*,*16 item version (DERS-16 Bjureberg et al., 2016). It measures lack of emotional clarity, difficulties in engaging in goal-directed behaviour, impulse control difficulties, limited access to effective emotion regulation strategies, and non-acceptance of emotional responses. Previous research has suggested the scale scores has favourable psychometric properties in adolescents with mental health issues (e.g., Charak et al., 2019).

Emotion regulation strategy use will be measured using the *Emotion Regulation Questionnaire* (42) adapted for children and adolescents (ERQ-CA Gullone & Taffe, 2012). It measures cognitive re-appraisal and suppression in two corresponding subscales of 6 and 4 items respectively. Previous research has suggested that the scale scores have favourable psychometric properties in adolescent samples (43).

Peer problems and friendships will be measured using the Social Relationships Questionnaire (SRQ). This is an 11-item measure of adolescent peer conflict and friendships initially developed for parent-report but adapted for self-report in the current study. Responses are recorded on a 4-point Likert-type scale from *not true* to *very true*. The parent-report version of this measure has been used in prior studies of conduct and peer problems in adolescents with ADHD (44,45).

Attachment security will be measured using the Inventory of Parent and Peer Attachment [IPPA-10;(46)]. This is a 10-item scale measuring respondents’ perceptions of trust, communication and alienation in their relationship with a primary caregiver. The scale also yields an overall attachment security score.

The *quality of teacher-student relationships* will be assessed by rating three statements: ‘I get along with my teacher’; ‘The teacher is fair to me’; and ‘The teacher supports me’ on a 4-point Likert scale indicating the level of agreement with each

(*completely untrue* to *completely* true). The questions have been used in another longitudinal study and have shown good reliability (alpha = .79) and validity (47).

Self-esteem will be measured using the Rosenberg Self-Esteem questionnaire (48). The scale includes 10 items (5 positively and 5 negatively worded) and an overall self-esteem score can be obtained by (after reverse coding as relevant) summation of the item responses.

Autistic traits will be measured using the 7-item Autism Symptom SElf-ReporT (ASSERT). Derived from a structured diagnostic interview (49), the scale measures social difficulties (4 items) and repetitive behaviour and circumscribed interests (3 items) (50).

Rejection sensitivity will be measured using a 6-item version of the *Child Rejection Sensitivity Questionnaire* (51,52). The scale presents respondents with 6 different scenarios that could result in rejection and for each they are asked to rate how anxious and angry they would feel prior to the outcome and how likely rejection would be. Anxious and anger rejection sensitivity are calculated by multiplication of the likelihood of rejection and corresponding anger/anxious scores for each scenario. Scores for each scenario can be summed to provide overall angry and anxious rejection scores.

Parents intake survey

Respondent gender will be measured using the same item as in the adolescent self-report questionnaire.

Respondent ethnicity will be measured using a set of items designed to be inclusive of different ethnic identities, with the additional options of “Prefer not to say” and “Not listed, please specify.”

Respondent education will be measured by asking respondents “what is the highest degree or level of school you have completed?” A set of items designed to be representative of the educational qualifications throughout the UK are provided with the additional options of “Prefer not to say” and “Not listed, please specify.”

Socioeconomic status will be estimated using SIMD and IMD based on postal code. Parents will also be asked whether their child is eligible for free school meals.

Child’s ADHD Diagnosis and other mental health/neurodevelopmental diagnoses. Parents will be asked for information on who diagnosed their child with ADHD as well as the child’s age at diagnosis. Further, parents will be asked whether their child has any other neurodevelopmental or mental health condition. They can choose from a list or specify other diagnoses in a free text field.

Child’s ADHD medication use. Parents will be asked which ADHD medications their child uses, dosage, and adherence (48). The latter will be measured using the 5-item Medication Adherence Report Scale (MARS, Chan et al., 2020).

Child’s ADHD strengths will be measured using the parent-report version of the above-described ADHD strengths measure (see self-report measures above). They will not be completed by parents of the control sample.

Child’s ADHD, conduct and oppositional symptoms will be measured using the *Disruptive Behavior Disorder Rating Scale* (49). The ADHD subscale includes 9 items measuring symptoms of inattention as well as 9 items measuring symptoms of hyperactivity and impulsivity, scored on a 4-point scale from *not at all* to *very much.* The scale was originally developed under DSM-III-R criteria, however, the symptoms assessed through this measure map on well to criteria outlined in DSM-V (American Psychiatric Association, 2013).

Child’s internalising problems will be measured with a parent-report version of the RCADS-11.

Child's peer conflict and friendships will be measured with the parent-report version of the SRQ.

Ecological momentary assessment measures

ADHD symptoms will be measured using a 3-item momentary symptoms scale developed by the team with reference to DSM criteria. The items are similar to EMA items used in previous EMA ADHD studies (e.g., Pedersen et al., 2020) and cover inattention, hyperactivity, and impulsivity.

Medication use will be measured by asking whether the young person has taken their medication since the last assessment.

Emotions will be measured using abbreviated versions of the Positive Affect Negative Affect Schedule expanded version (PANAS-X;Watson & Clark, 1999). Affect items were selected based on covering a wide range of emotions and being most relevant to ADHD and associated impairments. These will be used to derive indices of emotional lability, inertia, and reactivity within DSEM (53)

Emotion regulation strategies will be measured using an EMA-adapted version of the Emotion Regulation Questionnaire (54). Two items will be used to measure: suppression, and cognitive re-appraisal to experience less negative affect.

Peer interactions will be measured using a brief set of 4 items capturing experiences of *acceptance*, *victimisation, aggression*, and *positive peer interactions.* These items were developed by the team, drawing on previous EMA measures of social experiences (55) and considering common social difficulties among adolescents with ADHD.

Daily diary measures

Medication use will be measured using a two-part item asking whether and when the young person took ADHD medication in the last 24 hours.

Physical activity will be measured using three items asking respondents to report on the amount and intensity of physical activity undertaken in the last 24 hours adapted from an open EMA item pool (56).

Sleep will be measured using an abbreviated version of the Consensus Sleep Diary core items (57).

Adolescents end-of-survey measure

The adolescent end-of-survey questionnaire will be used to re-assess *anxiety and depression* with the RCADS-11. To inform the further development of EMA methodology with adolescents with ADHD, we will ask a brief set of items on their *participation experiences*, including the perceived burden of the EMA and daily diaries, how *enjoyable* the EMA was, how much it changed their *routine*. We will also include an open text field soliciting views on how to improve EMA data collections from a participant's perspective.

Parents end-of-survey measures

The parents end-of survey questionnaire will be used to re-assess their child's emotional and behavioural problems using the RCADS-11 and DBD. It will also measure parents' perspective on the burden of the EMA and daily diary studies. We will also include an open text field soliciting views on how to improve EMA data collections from a participant's perspective.

S2: Adolescents intake online survey

Gender Which of these best describes your gender?

- ☐ Male
- ☐ Female
- ☐ Non-binary
- ☐ Gender fluid
- ☐ Questioning
- ☐ I prefer to self-describe: ___________________________
- ☐ Prefer not to say

Age What is your date of birth (day/month/year)?

____

Ethnicity Which of the following most accurately describes you? Please select all that apply.

- ☐ Bangladeshi
- ☐ Pakistani
- ☐ Indian
- ☐ Chinese
- ☐ Any other Asian background
- ☐ Caribbean
- ☐ African
- ☐ Any other Black, Black British, or Caribbean background
- ☐ White and Black Caribbean
- ☐ White and Black African
- ☐ White and Asian
- ☐ Any other Mixed or multiple ethnic background
- ☐ English, Welsh, Scottish, Northern Irish, or British
- ☐ Irish
- ☐ Gypsy or Irish Traveller
- ☐ Roma
- ☐ Any other White background
- ☐ Arab
- ☐ Prefer not to say
- ☐ Not listed, please specify: ___________________________

Postcode What is your postcode?

____

RCADS-11 Please select the word that shows how often each of these things happened to you over the past two weeks. There are no right or wrong answers.

	Never	Sometimes	Often	Always
I have trouble going to school in the mornings because I feel nervous or afraid	○	○	○	○
I have no energy for things	○	○	○	○
I worry when I go to bed at night	○	○	○	○
I worry about what is going to happen	○	○	○	○
Nothing is much fun anymore	○	○	○	○
All of a sudden I feel really scared for no reason at all	○	○	○	○
I feel worthless	○	○	○	○
I feel sad or empty	○	○	○	○
When I have a problem, my heart beats really fast	○	○	○	○
I am tired a lot	○	○	○	○
I worry I might look foolish	○	○	○	○

Strengths For each item, please select the response that is most true for you.

	Strongly Agree	Agree	Neither Disagree nor Agree	Disagree	Strongly Disagree
I am able to focus really well on the things I am interested in	○	○	○	○	○
I have lots of energy to get things done	○	○	○	○	○
I have tons of ideas	○	○	○	○	○
I am creative	○	○	○	○	○
I have a warm personality	○	○	○	○	○
I am full of enthusiasm	○	○	○	○	○
I am good at coming up with solutions to problems	○	○	○	○	○
I find it easy to develop new interests (e.g., hobbies)	○	○	○	○	○

DERS-16 Please select the response that is most true for you.

	Almost Never	Sometimes	About half the time	Most of the time	Almost always
I have difficulty making sense out of my feelings	○	○	○	○	○
I am confused about how I feel	○	○	○	○	○
When I'm upset, I have difficulty getting work done	○	○	○	○	○
When I'm upset, I become out of control	○	○	○	○	○
When I'm upset, I believe that I will remain that way for a long time	○	○	○	○	○
When I'm upset, I believe that I'll end up feeling very depressed	○	○	○	○	○
When I'm upset, I have difficulty focusing on other things	○	○	○	○	○
When I'm upset, I feel out of control	○	○	○	○	○
When I'm upset, I feel ashamed with myself for feeling that way	○	○	○	○	○
When I'm upset, I feel like I am weak	○	○	○	○	○
When I'm upset, I have difficulty controlling my behaviours	○	○	○	○	○
When I'm upset, I believe there is nothing I can do to make myself feel better	○	○	○	○	○
When I'm upset, I become irritated with myself for feeling that way	○	○	○	○	○
When I'm upset, I start to feel very bad about myself	○	○	○	○	○

When I'm upset, I have difficulty thinking about anything else	○	○	○	○	○
When I'm upset, my emotions feel overwhelming	○	○	○	○	○

ERQ-CA We would now like to ask you some questions about your emotional life. For each item, please select the response that is most true for you.

	Strongly Disagree	Disagree	Half and Half	Agree	Strongly Agree
When I want to feel happier, I think about something different	○	○	○	○	○
I keep my feelings to myself	○	○	○	○	○
When I want to feel less bad (e.g., sad, angry, or worried), I think about something different	○	○	○	○	○
When I am feeling happy, I am careful not to show it	○	○	○	○	○
When I'm worried about something, I make myself think about it in a way that helps me feel better	○	○	○	○	○
I control my feelings by not showing them	○	○	○	○	○
When I want to feel happier about something, I change the way I'm thinking about it	○	○	○	○	○
I control my feelings about things by changing the way I think about them	○	○	○	○	○
When I'm feeling bad (e.g. sad, angry, or worried), I am careful not to show it	○	○	○	○	○
When I want to feel less bad (e.g. sad, angry, or worried) about something, I change the way I'm thinking about it	○	○	○	○	○

SRQ We would like to learn more about your friendships. Please indicate how much each of the following statements applies to you.

	Not True	Sort of True	Mostly True	Very True
You have a "best friend" that you feel close to	○	○	○	○

If "Mostly True" or "Very True" selected display the following questions:

	Not True	Sort of True	Mostly True	Very True
This relationship is positive and beneficial for you (affectionate, supportive, intimate)	○	○	○	○
This relationship is negative for you (high conflict, aggression, betrayal, competitive)	○	○	○	○

Display following questions to all participants:

	Not True	Sort of True	Mostly True	Very True
You have a group of friends at school that you hang around with	○	○	○	○
You have a group of friends at home that you like to play with	○	○	○	○
You have always had problems with friendships	○	○	○	○
You seem to have fewer friends as you get older	○	○	○	○
You have difficulty making new friends	○	○	○	○
You have difficulty keeping the friends that you make	○	○	○	○
Your friend(s) are similar to you in the way they act and the things they like	○	○	○	○

You have problems with other people your age because you tease them	○	○	○	○
You have problems with other people your age because you get teased by them	○	○	○	○
You have difficulty responding appropriately to teasing	○	○	○	○

Caregiver What relationship does the person taking care of you most of the time (i.e. your primary caregiver) have to you?

- ☐ Mother
- ☐ Father
- ☐ Other, please specify: ________________________
- ☐ Prefer not to say

IPPA-10 For each item, please select the response that is most true for you. Please give your answers thinking about the person who takes care of you most of the time (i.e. your primary caregiver).

	Very Untrue	Untrue	Sometimes Untrue	True	Very True
I don't get enough attention from my primary caregiver.	○	○	○	○	○
My primary caregiver accepts me the way I am.	○	○	○	○	○
When my primary caregiver knows something is bothering me, he/she will ask me about it.	○	○	○	○	○
I tell my primary caregiver about my problems and worries.	○	○	○	○	○
I get easily upset with my primary caregiver.	○	○	○	○	○
My primary caregiver respects my feelings.	○	○	○	○	○
I get mad at my primary caregiver.	○	○	○	○	○
My primary caregiver helps me to understand myself better.	○	○	○	○	○
When I talk about my problems with my primary caregiver, I feel ashamed or stupid.	○	○	○	○	○
When I get mad about something, my primary caregiver tries to understand that.	○	○	○	○	○

Teacher For each item, please select the response that is most true for you. Please give your answers thinking about the teacher who knows you best (e.g., your registration/form teacher).

	Completely Untrue	Mostly Untrue	Mostly True	Completely True
I get along with my teacher	○	○	○	○
My teacher is fair to me	○	○	○	○
My teacher supports me	○	○	○	○

Self-Esteem Below is a list of statements dealing with your general feelings about yourself. Please indicate how strongly you agree or disagree with each statement.

	Strongly Agree	Agree	Disagree	Strongly Disagree
On the whole, I am satisfied with myself	○	○	○	○
At times I think I am no good at all	○	○	○	○
I feel that I have a number of good qualities	○	○	○	○
I am able to do things as well as most other people	○	○	○	○
I feel I do not have much to be proud of	○	○	○	○
I certainly feel useless at times	○	○	○	○
I feel that I'm a person of worth, at least on an equal plane with others	○	○	○	○
I wish I could have more respect for myself	○	○	○	○
All in all, I am inclined to feel that I am a failure	○	○	○	○
I take a positive attitude toward myself	○	○	○	○

ASSERT Below is a list of statements dealing with your general feelings about yourself. Please indicate how strongly you agree or disagree with each statement.

	Not True	Somewhat True	Certainly True
Do you find it difficult to socialize with, or to get in touch with people, especially people your own age?	○	○	○
Do you prefer to be alone rather than being together with other people?	○	○	○
Do you have difficulties perceiving social cues?	○	○	○
Do other people tell you that your behavior or your emotional responses are inappropriate or hurtful?	○	○	○
Do you have a strong interest or hobby that absorbs so much of your time that it hampers other activities?	○	○	○
Do you or do other people feel that you have very set routines or that you are very immersed in your own interests?	○	○	○
Do you or do other people feel that you impose your routines or interests on others?	○	○	○

CRSQ Please imagine yourself in each of the following situations described here and select how you would feel in each.

Imagine you had a really bad fight the other day with a friend. Now you have a serious problem and you wish you had your friend to talk to. You decide to wait for your friend after class and talk with him/her. You wonder if your friend will want to talk to you.

1a How NERVOUS would you feel, RIGHT THEN, about whether or not your friend will want to talk to you and listen to your problem?

- ☐ not nervous
- ☐ 2
- ☐ 3
- ☐ 4
- ☐ 5
- ☐ very, very nervous

1b How MAD would you feel, RIGHT THEN, about whether or not your friend will want to talk to you and listen to your problem?

- ☐ not mad
- ☐ 2
- ☐ 3
- ☐ 4
- ☐ 5
- ☐ very, very mad

1c Do you think he/she will want to talk to you and listen to your problem?

- ☐ Yes
- ☐ 2
- ☐ 3
- ☐ 4
- ☐ 5
- ☐ No

Imagine that a famous person is coming to visit your school. Your teacher is going to pick five kids to meet this person. You wonder if she will choose you

2a How NERVOUS would you feel, RIGHT THEN, about whether or not the teacher will choose you?

- ☐ not nervous
- ☐ 2
- ☐ 3
- ☐ 4
- ☐ 5
- ☐ very, very nervous

2b How MAD would you feel, RIGHT THEN, about whether or not the teacher will choose you?

- ☐ not mad
- ☐ 2
- ☐ 3
- ☐ 4
- ☐ 5
- ☐ very, very mad

2c Do you think the teacher will choose YOU to meet the special guest?

- ☐ Yes
- ☐ 2
- ☐ 3
- ☐ 4
- ☐ 5
- ☐ No

Now imagine that you're back in class. Your teacher asks for a volunteer to help plan a party for your class. Lots of kids raise their hands so you wonder if the teacher will choose YOU.

3a How NERVOUS would you feel, RIGHT THEN, about whether or not the teacher will choose you?

- ☐ not nervous
- ☐ 2
- ☐ 3
- ☐ 4
- ☐ 5
- ☐ very, very nervous

3b How MAD would you feel, RIGHT THEN, about whether or not the teacher will choose you?

- ☐ not mad
- ☐ 2
- ☐ 3
- ☐ 4
- ☐ 5
- ☐ very, very mad

3c Do you think the teacher will choose YOU?

- ☐ Yes
- ☐ 2
- ☐ 3
- ☐ 4
- ☐ 5
- ☐ No

Imagine it's Saturday and you're carrying groceries home for your family. It is raining hard and you want to get home FAST. Suddenly, the paper bag you are carrying rips. All your food tumbles to the ground. You look up and see a couple of kids from your class walking quickly. You wonder if they will stop and help you

4a How NERVOUS would you feel, RIGHT THEN, about whether or not those kids will want to stop and help you?

- ☐ not nervous
- ☐ 2
- ☐ 3
- ☐ 4
- ☐ 5
- ☐ very, very nervous

4b How MAD would you feel, RIGHT THEN, about whether or not those kids will want to stop and help you?

- ☐ not mad
- ☐ 2
- ☐ 3
- ☐ 4
- ☐ 5
- ☐ very, very mad

4c Do you think they will offer to help you?

- ☐ Yes
- ☐ 2
- ☐ 3
- ☐ 4
- ☐ 5
- ☐ No

Pretend you have moved and you are going to a different school. In this school, the teacher lets the kids in the class take home a video game to play with on the weekend. Every week so far, you have watched someone else take it home. You decide to ask the teacher if YOU can take home the video game this time. You wonder if she will let you have it

5a How NERVOUS would you feel, RIGHT THEN, about whether or not the teacher will let you take the video game home this time?

- ☐ not nervous
- ☐ 2
- ☐ 3
- ☐ 4
- ☐ 5
- ☐ very, very nervous

5b How MAD would you feel, RIGHT THEN, about whether or not the teacher will let you take the video game home this time?

- ☐ not mad
- ☐ 2
- ☐ 3
- ☐ 4
- ☐ 5
- ☐ very, very mad

5c Do you think the teacher is going to let you take home the video game this time?

- ☐ Yes
- ☐ 2
- ☐ 3
- ☐ 4
- ☐ 5
- ☐ No

Imagine you’re back in your classroom, and everyone is splitting up into groups to work on a special project together. You sit there and watch lots of other kids getting picked. As you wait, you wonder if the kids will want you for their group.

6a How NERVOUS would you feel, RIGHT THEN, about whether or not they will choose you?

- ☐ not nervous
- ☐ 2
- ☐ 3
- ☐ 4
- ☐ 5
- ☐ very, very nervous

6b How MAD would you feel, RIGHT THEN, about whether or not they will choose you?

- ☐ not mad
- ☐ 2
- ☐ 3
- ☐ 4
- ☐ 5
- ☐ very, very mad

6c Do you think the the kids in your class will choose you for their group?

- ☐ Yes
- ☐ 2
- ☐ 3
- ☐ 4
- ☐ 5
- ☐ No

S3: EMA and Daily Diary measures

EMA Measures

ADHD symptoms (inattention, hyperactivity, impulsivity)

I couldn't concentrate

Strongly agree	Agree	Neither agree nor disagree	Disagree	Strongly disagree

I acted before thinking

Strongly agree	Agree	Neither agree nor disagree	Disagree	Strongly disagree

I was hyperactive

Strongly agree	Agree	Neither agree nor disagree	Disagree	Strongly disagree

Emotions (PANAS)

In the last 30 minutes I felt…

Excited

Very slightly or not at all	A little	Moderately	Quite a bit	Extremely

Upset

Very slightly or not at all	A little	Moderately	Quite a bit	Extremely

Determined

Very slightly or not at all	A little	Moderately	Quite a bit	Extremely

Enthusiastic

Very slightly or not at all	A little	Moderately	Quite a bit	Extremely

Ashamed

Very slightly or not at all	A little	Moderately	Quite a bit	Extremely

Nervous

Very slightly or not at all	A little	Moderately	Quite a bit	Extremely

Emotion regulation strategy use

In the last 30 minutes, I…

Kept my emotions to myself

Strongly Disagree	Disagree	Neither agree nor disagree	Agree	Strongly Agree

When I wanted to feel less negative emotion (such as sadness or anger), I changed what I was thinking about

Strongly Disagree	Disagree	Neither agree nor disagree	Agree	Strongly Agree

Peer interactions

In relation to my peers (other adolescents my age) in the last 30 minutes, I…

Felt accepted

Strongly Disagree	Disagree	Neither agree nor disagree	Agree	Strongly Agree

Was teased

Strongly Disagree	Disagree	Neither agree nor disagree	Agree	Strongly Agree

Got into an argument

Strongly Disagree	Disagree	Neither agree nor disagree	Agree	Strongly Agree

I enjoyed interacting (online or in person) with others my age.

Strongly Disagree	Disagree	Neither agree nor disagree	Agree	Strongly Agree

Daily Diary Measures

Physical activity

How many minutes did you do vigorous activities including intensive sports or exercise (such as running or fast cycling) or intensive physical work (such as heavy lifting or digging)?

How many minutes did you do moderate activities (activities that make you breathe somewhat harder than usual such as playing tennis, bicycling, carrying light loads)?

How many minutes did you do light activities (activities that may not make you breathe somewhat harder than usual such as walking, climbing stairs, routine household chores, etc.)?

Medication use

Have you taken ADHD medication in the last 24 hours?

Sleep (consensus sleep scale core items)

What time did you get into bed last night?

What time did you try to go to sleep?

How long did it take you to fall asleep (in minutes)?

How many times did you wake up, not counting your final awakening?

In total, how long did these awakenings last (in minutes)?

What time was your final awakening?

How would you rate quality of your sleep?

Very poor	Poor	Fair	Good	Very Good

Comments (if applicable)

S4: Adolescents end-of-study survey

RCADS-11 Please select the word that shows how often each of these things happened to you over the past two weeks. There are no right or wrong answers.

	Never	Sometimes	Often	Always
I have trouble going to school in the mornings because I feel nervous or afraid	○	○	○	○
I have no energy for things	○	○	○	○
I worry when I go to bed at night	○	○	○	○
I worry about what is going to happen	○	○	○	○
Nothing is much fun anymore	○	○	○	○
All of a sudden I feel really scared for no reason at all	○	○	○	○
I feel worthless	○	○	○	○
I feel sad or empty	○	○	○	○
When I have a problem, my heart beats really fast	○	○	○	○
I am tired a lot	○	○	○	○
I worry I might look foolish	○	○	○	○

EMA feedback We would love to know how taking part in the smart-phone based surveys was for you. Please select the response that is most true for you thinking about your experiences of completing the smartphone-based surveys.

	Almost never	Sometimes	About half the time	Most of the time	Almost always
I found doing the smartphone surveys fun	○	○	○	○	○
I found doing the smartphone surveys hard work	○	○	○	○	○
Doing the smartphone surveys disrupted my routine	○	○	○	○	○

Suggestions Do you have thoughts or suggestions on how the smartphone surveys could be improved?

____

S5: Parents online intake survey

Gender Which of these best describes your gender?

- ☐ Male
- ☐ Female
- ☐ Non-binary
- ☐ Gender fluid
- ☐ Questioning
- ☐ I prefer to self-describe: ___________________________
- ☐ Prefer not to say

Age What is your date of birth (day/month/year)?

____

Ethnicity Which of the following most accurately describes you? Please select all that apply.

- ☐ Bangladeshi
- ☐ Pakistani
- ☐ Indian
- ☐ Chinese
- ☐ Any other Asian background
- ☐ Caribbean
- ☐ African
- ☐ Any other Black, Black British, or Caribbean background
- ☐ White and Black Caribbean
- ☐ White and Black African
- ☐ White and Asian
- ☐ Any other Mixed or multiple ethnic background
- ☐ English, Welsh, Scottish, Northern Irish, or British
- ☐ Irish
- ☐ Gypsy or Irish Traveller
- ☐ Roma
- ☐ Any other White background
- ☐ Arab
- ☐ Prefer not to say
- ☐ Not listed, please specify: ___________________________

Education What is the highest degree or level of school you have completed?

- ☐ Less than a high school diploma
- ☐ High school or equivalent (e.g. Scottish National level 5, GCSE)
- ☐ College/Sixth form (e.g. Scottish Highers/Higher National Certificate, Advanced Highers/Higher National Diploma, BTEC, NVQ, AS/A-levels)
- ☐ Further education college (e.g. HNC, HND)
- ☐ Vocational (e.g. apprenticeship, higher apprenticeship)
- ☐ Bachelor's degree (e.g. BA, BSc)
- ☐ Master's degree (e.g. MA, MSc, MEd)
- ☐ Doctorate or professional degree (e.g. MD, DDS, PhD)
- ☐ Prefer not to say
- ☐ Not listed, please specify: ___________________________

Postcode What is your postcode?

____

Meals Is your child eligible for free school meals?

- ☐ Yes
- ☐ No

Diagnosis Source Who diagnosed your child with ADHD?

- ☐ General Practitioner
- ☐ Pediatrician
- ☐ Psychiatrist
- ☐ Psychologist
- ☐ Neurologist
- ☐ Other, please specify: ________________________________

Diagnosis Age How old was your child when they were diagnosed with ADHD?

Diagnoses Other Does your child have any other mental health or neurodevelopmental diagnoses?

- ☐ Yes
- ☐ No

If yes, display this question:

Diagnoses List Which other diagnoses does your child have? Please select all that apply.

- ☐ Autism Spectrum Disorder
- ☐ Foetal Alcohol Syndrome
- ☐ Intellectual Disability
- ☐ Anxiety Disorder
- ☐ Depression
- ☐ Conduct Disorder
- ☐ Other, please specify: ________________________________

Medication Is your child currently taking any ADHD medication?

- ☐ Yes
- ☐ No

If yes, display this question:

Medication Details Which medicine is your child taking and what dosage?

- ☐ Medicine: ___________________________
- ☐ Dosage (e.g., XX mg, 2x a day): ___________________________

Medication Adherence Please select the response that is most true for your child.

	Always	Often	Sometimes	Rarely	Never
My child forgets to take their medication	○	○	○	○	○
My child changes the dosage of their medication	○	○	○	○	○
My child stops taking their medication for a while	○	○	○	○	○
My child decides to skip one of their medication dosages	○	○	○	○	○
My child uses their medication less than is prescribed	○	○	○	○	○

Strengths Please select the response that is most true for your child.

	Strongly Agree	Agree	Neither Disagree nor Agree	Disagree	Strongly Disagree
My child is able to focus really well on the things they are interested in	○	○	○	○	○
My child has lots of energy to get things done	○	○	○	○	○
My child has tons of ideas	○	○	○	○	○
My child is creative	○	○	○	○	○
My child has a warm personality	○	○	○	○	○
My child is full of enthusiasm	○	○	○	○	○
My child is good at coming up with solutions to problems	○	○	○	○	○
My child finds it easy to develop new interests (e.g., hobbies)	○	○	○	○	○

DBD Please select the option that best describes your child. Please give your answers on the basis of your child's behaviour over the last six months

	Not at All	Just a Little	Pretty Much	Very Much	Don't Know or Not Applicable
Often interrupts or intrudes on others (e.g., butts into conversations or games)	○	○	○	○	○
Has run away from home overnight at least twice while living in parental or parental surrogate home (or once without returning for a lengthy period)	○	○	○	○	○
Often argues with adults	○	○	○	○	○
Often lies to obtain goods or favors or to avoid obligations (i.e., "cons" others)	○	○	○	○	○
Often initiates physical fights with other members of his or her household	○	○	○	○	○
Has been physically cruel to people	○	○	○	○	○
Often talks excessively	○	○	○	○	○
Has stolen items of nontrivial value without confronting a victim (e.g., shoplifting, but without breaking and entering; forgery)	○	○	○	○	○
Is often easily distracted by extraneous stimuli	○	○	○	○	○

	Not at All	Just a Little	Pretty Much	Very Much	Don't Know or Not Applicable
Often engages in physically dangerous activities without considering possible consequences (not for the purpose of thrill-seeking), e.g., runs into street without looking	○	○	○	○	○
Often truant from school, beginning before age 13 years	○	○	○	○	○
Often fidgets with hands or feet or squirms in seat	○	○	○	○	○
Is often spiteful or vindictive	○	○	○	○	○
Often swears or uses obscene language	○	○	○	○	○
Often blames others for his or her mistakes or misbehavior	○	○	○	○	○
Has deliberately destroyed others' property (other than by fire setting)	○	○	○	○	○
Often actively defies or refuses to comply with adults' requests or rules	○	○	○	○	○
Often does not seem to listen when spoken to directly	○	○	○	○	○
Often blurts out answers before questions have been completed	○	○	○	○	○
Often initiates physical fights with others who do not live in his or her household (e.g., peers at school or in the neighborhood)	○	○	○	○	○
Often shifts from one uncompleted activity to another	○	○	○	○	○
Often has difficulty playing or engaging in leisure activities quietly	○	○	○	○	○

	Not at All	Just a Little	Pretty Much	Very Much	Don't Know or Not Applicable
Often fails to give close attention to details or makes careless mistakes in schoolwork, work, or other activities	○	○	○	○	○
Is often angry and resentful	○	○	○	○	○
Often leaves seat in classroom or in other situations in which remaining seated is expected	○	○	○	○	○
Is often touchy or easily annoyed by others	○	○	○	○	○
Often does not follow through on instructions and fails to finish schoolwork, chores, or duties in the workplace (not due to oppositional behavior or failure to understand instructions)	○	○	○	○	○
Often loses temper	○	○	○	○	○
Often has difficulty sustaining attention in tasks or play activities	○	○	○	○	○
Often has difficulty awaiting turn	○	○	○	○	○
Has forced someone into sexual activity	○	○	○	○	○
Often bullies, threatens, or intimidates others	○	○	○	○	○
Is often "on the go" or often acts as if "driven by a motor"	○	○	○	○	○
Often loses things necessary for tasks or activities (e.g., toys, school assignments, pencils, books, or tools)	○	○	○	○	○

Often runs about or climbs excessively in situations in which it is inappropriate (in adolescents or adults, may be limited to subjective feelings of restlessness)	○	○	○	○	○
Has been physically cruel to animals	○	○	○	○	○
Often avoids, dislikes, or is reluctant to engage in tasks that require sustained mental effort (such as schoolwork or homework)	○	○	○	○	○
Often stays out at night despite parental prohibitions, beginning before age 13 years	○	○	○	○	○
Often deliberately annoys people	○	○	○	○	○
Has stolen while confronting a victim (e.g., mugging, purse snatching, extortion, armed robbery)	○	○	○	○	○
Has deliberately engaged in fire setting with the intention of causing serious damage	○	○	○	○	○
Often has difficulty organizing tasks and activities	○	○	○	○	○
Has broken into someone else's house, building, or car	○	○	○	○	○
Is often forgetful in daily activities	○	○	○	○	○
Has used a weapon that can cause serious physical harm to others (e.g., a bat, brick, broken bottle, knife, gun)	○	○	○	○	○

RCADS-11 Please select the option that best describes your child. Please give your answers on the basis of your child's behaviour over the last six months.

	Never	Sometimes	Often	Always
My child has trouble going to school in the mornings because of feeling nervous or afraid	○	○	○	○
My child has no energy for things	○	○	○	○
My child worries when in bed at night	○	○	○	○
My child worries about what is going to happen	○	○	○	○
Nothing is much fun for my child anymore	○	○	○	○
All of a sudden my child will feel really scared for no reason at all	○	○	○	○
My child feels worthless	○	○	○	○
My child feels sad or empty	○	○	○	○
When my child has a problem, his/her heart beats really fast	○	○	○	○
My child is tired a lot	○	○	○	○
My child worries about looking foolish	○	○	○	○

SRQ We would like to learn more about your child's peer relationships. Please indicate how much each of the following statements applies to your child.

	Not True	Sort of True	Mostly True	Very True
My child has a "best friend" that he/she feels close to	○	○	○	○

If "Mostly True" or "Very True" selected display the following questions:

	Not True	Sort of True	Mostly True	Very True
This relationship is positive and beneficial for my child (affectionate, supportive, intimate)	○	○	○	○
This relationship is negative for my child (high conflict, aggression, betrayal, competitive)	○	○	○	○

Display following questions to all participants:

	Not True	Sort of True	Mostly True	Very True
My child has a group of friends at school that he/she hangs around with	○	○	○	○
My child has a group of friends at home that he/she likes to play with	○	○	○	○
My child has always had problems with peer relationships	○	○	○	○
My child seems to have fewer friends as he/she grows older	○	○	○	○
My child has difficulty making new friends	○	○	○	○
My child has difficulty keeping the friends that he/she makes	○	○	○	○

My child's friend(s) are similar to him/her in the way they act and the things they like	○	○	○	○
My child has problems with other children because he/she teases them	○	○	○	○
My child has problems with other children because he/she gets teased by them	○	○	○	○
My child has difficulty responding appropriately to teasing	○	○	○	○

S6: Parents end-of-study survey

DBD Please select the option that best describes your child. Please give your answers on the basis of your child's behaviour over the last six months

	Not at All	Just a Little	Pretty Much	Very Much	Don't Know or Not Applicable
Often interrupts or intrudes on others (e.g., butts into conversations or games)	○	○	○	○	○
Has run away from home overnight at least twice while living in parental or parental surrogate home (or once without returning for a lengthy period)	○	○	○	○	○
Often argues with adults	○	○	○	○	○
Often lies to obtain goods or favors or to avoid obligations (i.e., "cons" others)	○	○	○	○	○
Often initiates physical fights with other members of his or her household	○	○	○	○	○
Has been physically cruel to people	○	○	○	○	○
Often talks excessively	○	○	○	○	○
Has stolen items of nontrivial value without confronting a victim (e.g., shoplifting, but without breaking and entering; forgery)	○	○	○	○	○
Is often easily distracted by extraneous stimuli	○	○	○	○	○

	Not at All	Just a Little	Pretty Much	Very Much	Don't Know or Not Applicable
Often engages in physically dangerous activities without considering possible consequences (not for the purpose of thrill-seeking), e.g., runs into street without looking	○	○	○	○	○
Often truant from school, beginning before age 13 years	○	○	○	○	○
Often fidgets with hands or feet or squirms in seat	○	○	○	○	○
Is often spiteful or vindictive	○	○	○	○	○
Often swears or uses obscene language	○	○	○	○	○
Often blames others for his or her mistakes or misbehavior	○	○	○	○	○
Has deliberately destroyed others' property (other than by fire setting)	○	○	○	○	○
Often actively defies or refuses to comply with adults' requests or rules	○	○	○	○	○
Often does not seem to listen when spoken to directly	○	○	○	○	○
Often blurts out answers before questions have been completed	○	○	○	○	○
Often initiates physical fights with others who do not live in his or her household (e.g., peers at school or in the neighborhood)	○	○	○	○	○
Often shifts from one uncompleted activity to another	○	○	○	○	○
Often has difficulty playing or engaging in leisure activities quietly	○	○	○	○	○

	Not at All	Just a Little	Pretty Much	Very Much	Don't Know or Not Applicable
Often fails to give close attention to details or makes careless mistakes in schoolwork, work, or other activities	○	○	○	○	○
Is often angry and resentful	○	○	○	○	○
Often leaves seat in classroom or in other situations in which remaining seated is expected	○	○	○	○	○
Is often touchy or easily annoyed by others	○	○	○	○	○
Often does not follow through on instructions and fails to finish schoolwork, chores, or duties in the workplace (not due to oppositional behavior or failure to understand instructions)	○	○	○	○	○
Often loses temper	○	○	○	○	○
Often has difficulty sustaining attention in tasks or play activities	○	○	○	○	○
Often has difficulty awaiting turn	○	○	○	○	○
Has forced someone into sexual activity	○	○	○	○	○
Often bullies, threatens, or intimidates others	○	○	○	○	○
Is often "on the go" or often acts as if "driven by a motor"	○	○	○	○	○
Often loses things necessary for tasks or activities (e.g., toys, school assignments, pencils, books, or tools)	○	○	○	○	○

Often runs about or climbs excessively in situations in which it is inappropriate (in adolescents or adults, may be limited to subjective feelings of restlessness)	○	○	○	○	○
Has been physically cruel to animals	○	○	○	○	○
Often avoids, dislikes, or is reluctant to engage in tasks that require sustained mental effort (such as schoolwork or homework)	○	○	○	○	○
Often stays out at night despite parental prohibitions, beginning before age 13 years	○	○	○	○	○
Often deliberately annoys people	○	○	○	○	○
Has stolen while confronting a victim (e.g., mugging, purse snatching, extortion, armed robbery)	○	○	○	○	○
Has deliberately engaged in fire setting with the intention of causing serious damage	○	○	○	○	○
Often has difficulty organizing tasks and activities	○	○	○	○	○
Has broken into someone else's house, building, or car	○	○	○	○	○
Is often forgetful in daily activities	○	○	○	○	○
Has used a weapon that can cause serious physical harm to others (e.g., a bat, brick, broken bottle, knife, gun)	○	○	○	○	○

RCADS-11 Please select the option that best describes your child. Please give your answers on the basis of your child's behaviour over the last six months.

	Never	Sometimes	Often	Always
My child has trouble going to school in the mornings because of feeling nervous or afraid	○	○	○	○
My child has no energy for things	○	○	○	○
My child worries when in bed at night	○	○	○	○
My child worries about what is going to happen	○	○	○	○
Nothing is much fun for my child anymore	○	○	○	○
All of a sudden my child will feel really scared for no reason at all	○	○	○	○
My child feels worthless	○	○	○	○
My child feels sad or empty	○	○	○	○
When my child has a problem, his/her heart beats really fast	○	○	○	○
My child is tired a lot	○	○	○	○
My child worries about looking foolish	○	○	○	○

EMA feedback We would appreciate getting your feedback on the smart-phone based surveys that your child took part in.

	Almost never	Sometimes	About half the time	Most of the time	All of the time
My child found doing the smartphone surveys fun	○	○	○	○	○
I would allow my child to participate in a study like this involving smartphone surveys again	○	○	○	○	○

Suggestions Do you have thoughts or suggestions on how the smartphone surveys could be improved?

____
