# Supplementary material for: Illuminating the daily life experiences of adolescents with and without ADHD: protocol for an ecological momentary assessment study
Source: BMJ Open. 2023 Sep 29;13(9):e077222. doi: 10.1136/bmjopen-2023-077222 (PMC10546102; doi:10.1136/bmjopen-2023-077222)
Supplement: Author's manuscript [file bmjopen-2023-077222.draft_revisions.pdf]

BMJ Open is committed to open peer review. As part of this commitment we make the peer review history of every article we publish publicly available.

When an article is published we post the peer reviewers' comments and the authors' responses online. We also post the versions of the paper that were used during peer review. These are the versions that the peer review comments apply to.

The versions of the paper that follow are the versions that were submitted during the peer review process. They are not the versions of record or the final published versions. They should not be cited or distributed as the published version of this manuscript.

BMJ Open is an open access journal and the full, final, typeset and author-corrected version of record of the manuscript is available on our site with no access controls, subscription charges or pay-per-view fees (http://bmjopen.bmj.com).

If you have any questions on BMJ Open's open peer review process please email info.bmjopen@bmj.com

Illuminating the daily life experiences of adolescents with and without ADHD: Protocol for an ecological momentary assessment study

Journal:	*BMJ Open*
Manuscript ID	bmjopen-2023-077222
Article Type:	Protocol
Date Submitted by the Author:	28-Jun-2023
Complete List of Authors:	Murray, Aja; University of Edinburgh, Department of Psychology<br>Speyer, Lydia; University of Edinburgh<br>Thye, Melissa; University of Edinburgh, Department of Psychology<br>Stewart, Tracy; University of Edinburgh<br>Obsuth, Ingrid; University of Edinburgh, Clinical and Health Psychology Department<br>Kane, Jennifer; University of Edinburgh, Clinical and Health Psychology Department<br>Whyte, Katie; St Andrew's University, Department of Psychology<br>Devaney, John; University of Edinburgh<br>Rohde, Luis; Universidade Federal do Rio Grande do Sul<br>Ushakova, Anastasia; Lancaster University<br>Rhodes, Sinead ; University of Edinburgh
Keywords:	Child & adolescent psychiatry < PSYCHIATRY, Depression & mood disorders < PSYCHIATRY, Anxiety disorders < PSYCHIATRY

SCHOLARONE™
Manuscripts

Illuminating the daily life experiences of adolescents with and without ADHD: Protocol for an ecological momentary assessment study

Aja Louise Murray[1], Lydia Speyer[1,2], Melissa Thye[1], Tracy M. Stewart[3], Ingrid Obsuth[4], Jennifer Kane[4], Katie Whyte[5], John Devaney[6], Luis Rohde[7], Anastasia Ushakova[8], Sinead Rhodes[9]

[1]Department of Psychology, University of Edinburgh, UK

[2]Department of Psychology, University of Cambridge, UK

[3]Moray School House of Education and Sport, University of Edinburgh, UK

[4]Clinical and Health Psychology Department, University of Edinburgh, UK

[5]Department of Psychology, St Andrew's University, UK

[6] School of Social and Political Science, University of Edinburgh, UK

[7] ADHD Outpatient Program and Developmental Psychiatry Program, Hospital de Clinicas de Porto Alegre, Federal University of Rio Grande do Sul, Porto Alegre, Brazil, UniEduk, Brazil and National Institute of Developmental Psychiatry & National Center for Research and Innovation in Mental Health, Sao Paulo, Brazil

[8] Centre for Computing, Health Informatics and Statistics, Lancaster University, United Kingdom

[9]Centre for Clinical Brain Sciences, University of Edinburgh, UK

*Corresponding author at: 7 George Square, Edinburgh, EH8 9JZ, email: aja.murray@ed.ac.uk

For peer review only

Abstract

Introduction

Adolescents with Attention-Deficit/Hyperactivity Disorder (ADHD) are at elevated risk of a range of difficulties, among which emotion regulation, peer, and co-occurring mental health problems are prominent challenges. To better support adolescents with ADHD, ecologically valid interventions that can be embedded in daily life to target the most proximal antecedents of these challenges are needed. Ecological momentary assessment (EMA) designs are ideally suited to meeting this need.

Methods and analyses

In the mental health in the moment ADHD (MHIM-ADHD) study, we will use an EMA design to capture the daily life experiences of approximately 120 adolescents aged 11-14 years with a clinical diagnosis of ADHD and the same number of age- and gender-matched peers without a diagnosis of ADHD. We will combine this with comprehensive information gathered from online surveys. Analysing the data using techniques such as dynamic structural equation modelling (DSEM), we will examine, among other research questions, the role of emotion regulation and peer problems in mediating the links between characteristics of ADHD and commonly co-occurring outcomes such as anxiety, depression, and conduct problems. The results can help inform interventions to support improved peer functioning and emotion regulation for adolescents with ADHD.

Ethics and dissemination

This study received a favourable ethical opinion through the National Health Service (NHS) ethical review board and the local university institutional review board of the first author. Results will be disseminated through journal publications, conferences and seminar presentations and to relevant stakeholders, such as those with ADHD, their families, and clinicians.

Keywords

Attention Deficit Hyperactivity Disorder/ADHD, Ecological Momentary Assessment, Emotion Regulation, Peer Problems

Strengths and limitations of this study

- This study will use an ecological momentary assessment design to collect high temporal resolution, ecologically value daily life experience data from young people with ADHD.
- It will use cutting edge statistical analysis techniques to disentangle different aspects of emotional functioning in daily life to identify key intervention targets.
- A limitation is that data will be collected only over a short period, therefore, developmental changes will not be captured.
- Further, EMA methods may be vulnerable to low response rates, careless responding, or drop-out and the reliance on self-reports can be associated with reporting biases.

For peer review only

Introduction

ADHD symptoms may increase the risk of experiencing a range of difficulties in adolescence (1–3) and prominent among these are inter-related challenges in emotion regulation and peer relationships (4). These have been suggested to contribute to the development of co-occurring mental health conditions such as anxiety, depression, and conduct problems (5–7); outcomes identified as a high priority for further research in consultations with neurodivergent expert-by-experience consultations (Embracing Complexity, 2022). However, critical reviews (8–10) have suggested a strong need for better interventions to support neurodivergent adolescents facing these challenges. In particular, current interventions have been criticised for a lack of generalisability to real-life contexts, for not directly targeting the interconnections between emotion dysregulation and peer problems, and for a lack of tailoring to the specific needs of adolescents (e.g., Morris et al., 2021). The goal of the present study is to provide evidence that helps address this gap, using a data collection technique that gathers rich 'in-the-moment' data about the daily life experiences and functioning of adolescents with and without ADHD.

Difficulties with emotion regulation among children and adolescents with ADHD are well documented (4,11). Emotion regulation can be defined as the set of processes by which individuals evaluate, inhibit, or modify their emotional reactions in order to show a socially appropriate behaviour and/or achieve some goal (12). Emotional dysregulation may manifest in a number of ways, including in impaired emotion recognition or understanding, a lack of empathy/callous-unemotional traits and emotional lability or reactivity. In comparison with neurotypical peers, youth with ADHD show greater emotional lability or reactivity (13) characterized by more intense, under-controlled, or rapidly shifting emotions than are appropriate for developmental stage and context and which consequently impacts functioning (14). This may include dysregulation of positive emotions leading to excessive excitability (1).

Emotion dysregulation often exists alongside and has been proposed to contribute to peer difficulties such as bullying victimisation and perpetration, peer aggression, rejection, dislike, and conflict among adolescents with ADHD (1,15). As a result of emotion regulation difficulties, adolescents with ADHD may react more strongly and more aggressively to provocations, making them targets for victimisation and placing them at higher risk of showing reactive aggression (16). They may become over-excited and experience resultant difficulties in attending to what is required of them in a given social situation (1). A role for rejection sensitivity, defined as the tendency to anxiously anticipate, over-perceive, and react intensely to social rejection (potentially arising as a result of repeated experiences of rejection), has also been proposed (17,18). Intense and dysregulated reactions to perceived rejection (e.g., excessive reassurance seeking or anger) may be experienced as aversive to peers and result in a 'self-fulfilling prophecy' of peer rejection (18). Overall, it has been noted that some youth with ADHD, as a result of emotion regulation difficulties, may be perceived as intense, disruptive, or overly exuberant, potentially leading to greater exposure to dislike, rejection, and victimisation (15). Indeed, given their inter-connectedness, the self-regulation of emotions, alongside behavioural inhibition, is thought to be one of the most important aspects of interventions to support better social functioning in adolescence (9).

Emotion dysregulation and peer problems are also thought to contribute to the development of co-occurring mental health and behavioural challenges associated with ADHD symptoms. For example, previous research in children and adolescents has found that peer issues and emotion regulation mediate the associations between ADHD symptoms and internalising problems such as anxiety and depression (5,6,19,20) and externalising problems such as aggression, oppositional, and conduct problem behaviours (e.g., Slaughter et al., 2019; Yip et al., 2013).

However, the daily life mechanisms underpinning these links and - correspondingly - the most proximal targets for intervention remain poorly understood, particularly in the critical

For peer review only - http://bmjopen.bmj.com/site/about/guidelines.xhtml

period of adolescence. Ecological momentary assessment (EMA) has proven valuable in child and adolescent mental health research for capturing symptoms and related experiences in near real time and in the flow of daily life and can thus inform more ecologically embedded intervention approaches (Russell & Gajos, 2020). It is particularly valuable for capturing concepts such as emotional (dys)regulation and social interactions in daily life contexts (6). For example, EMA data can be used to provide measures of different aspects of emotional functioning in daily life, such as overall levels of negative affect, affective lability, emotional inertia, and emotional reactivity to stressors or provocations (24,25).

There have, however, been only a handful EMA studies of young people with ADHD during the critical period of adolescence (26,27). Reviewing the literature on EMA studies of ADHD populations, Koch et al. (2021) identified less than 10 studies conducted in adolescents, most of which had small sample sizes and none of which addressed the links between emotion regulation, peer issues and co-occurring mental health and behavioural outcomes. Only one previous EMA study has examined the impact of emotion regulation on peer issues and mental health outcomes in a sample of adolescents with ADHD. Using a daily diary design, Breaux et al. (2020) found that adolescents with ADHD tend to experience more negative emotion, less positive emotion, and more emotional variability. Variability in some emotions was associated with outcomes such as social functioning and externalising problems.

These initial findings point to the value of further exploring, using EMA, the daily life manifestation and impacts of emotion regulation and peer interactions in adolescents with ADHD. For example, as well as seeking to extend these findings to examine a wider range of emotion regulation and peer issues, it will be valuable to examine factors that may impact these domains, their inter-connections, and their links to mental health and behavioural outcomes This will provide illumination on further modifiable targets for intervention, such as physical activity, improved sleep, secure attachment to caregivers, and positive teacher-student relationships (25,28–30). Further, strengths associated with ADHD characteristics, such as a high level of energy and drive, the ability to focus intensely in tasks of interest, and positive temperamental factors (31) will be explored as these too could potentially be leveraged as part of more effective interventions.

We will thus use an EMA design to illuminate how, in the flow of their daily lives, adolescents with ADHD may experience difficulties with emotion regulation and peer problems, how these may contribute to co-occurring mental health and behavioural problems. We will also examine other potential modifiable targets for intervention that may impact emotion regulation, peer problems, mental health and behaviour as well as their links among adolescents with ADHD (e.g., ADHD strengths, relationships, sleep, physical activity). Core research questions are:

- Which aspects of emotion regulation differ between adolescents with and without ADHD, e.g., negative or positive emotional lability, emotional reactivity, emotional inertia, emotion regulation strategy use and emotion regulation strategy success?
- Which aspects of emotion regulation mediate the association between ADHD diagnosis/symptoms and peer problems in daily life?
- Do emotion regulation and peer issues mediate the links between ADHD diagnosis/symptoms and anxiety, depression and conduct problems?

Additional exploratory analyses may be used to answer a range of additional questions such as exploration of moderation of the links between ADHD diagnosis/symptoms and outcomes to identify other potential intervention targets.

For peer review only

Methods and analyses

Participants

Participants are expected to include approximately 120 adolescents aged 11-14 years with a clinical diagnosis of ADHD and 120 matched controls. The clinical sample will be recruited via Child and Adolescent Mental Health Services (CAMHS) in two primary sites in the UK (one in Scotland and one in England) and community partners. In CAMHS, the recruitment procedure will vary from service to service; however, in general participants will be recruited at medication clinics and through ongoing casework.

A matched control (on age +/- 3 months, gender, and if possible, city and socioeconomic status based on Scottish Index of Multiple Deprivation in Scotland and Index of Multiple Deprivation in England) will be selected for each participant with a clinical diagnosis of ADHD from a larger pool of participants taking part in adolescent mental health studies (co-)led by the first author. To maximise relevance to community-ascertained samples, minor modifications to the protocol will be made, detailed in the Measures section.

Inclusion and exclusion criteria

Inclusion criteria for the clinical sample will be: aged 11-14, a formal clinical diagnosis of ADHD (parent-reported in the case of the youth recruited via the community), the ability to provide informed assent and parental informed consent to participate, and the ability to independently complete online surveys and EMA protocols. There will be no explicit exclusion criteria based on co-occurring neurodevelopmental conditions as ADHD commonly co-occurs with other neurodevelopmental conditions (e.g., Rong et al., 2021) and excluding youth with these conditions would undermine the representativeness of the sample. It would also preclude the opportunity to illuminate how ADHD characteristics may interact with those of other neurodevelopmental conditions (e.g., Rosello et al., 2022). For similar reasons and given our goal of illuminating candidate mechanisms by which ADHD characteristics may impact mental health and behavioural outcomes, we will also not impose any exclusion criteria related to any other co-occurring psychiatric issues (e.g., anxiety, depression, conduct disorder). However, we acknowledge that the requirement to be able to complete the online surveys and EMA surveys independently may result in the exclusion of some young people (e.g., those with a moderate to severe intellectual disability). Adaptation of the protocol to better include these groups will be the subject of future research. Inclusion and exclusion criteria for the control sample will be identical, except that a formal clinical diagnosis of ADHD will be an exclusion criterion for controls.

Ethics and dissemination

Ethical review for the collection of the data from the clinically ascertained sample will follow a three-stage process in which sponsorship will be sought from ACCORD, followed by ethical review by the National Health Service (NHS) ethical review board, and finally, research and development approval. Ethical review for the collection of data based on community and school recruitment will be by the local university institutional review board of the first author. Results will be disseminated through journal publications, conferences and seminar presentations and to relevant stakeholders, such as those with ADHD, their families, and clinicians.

Patient and public involvement

Adolescents with ADHD and parents of adolescents with ADHD are the most important stakeholders in this project. Their views have informed the project design,

For peer review only

including the current protocol and will continue to inform the project and the dissemination of its outputs. Young person and parent advisors were recruited via the ADHD charities and organisations with whom our team has collaborative links. Examples of major changes made to the protocol based on their input included extending the inclusion criteria to include lower ages, reducing the length of the surveys, including more positively- and fewer negatively-worded items, removing elements of the project that would have involved location tracking and wearable technology, adding a measure of rejection sensitivity, and embedding more fun and educational elements into the study, such as providing 'ADHD facts' at the end of each completed EMA survey.

Data collection

Data collection is projected to begin in August 2023 and be completed in 12 months. Figure 1 summarises the study flow. Informed parental consent and adolescent assent will be collected, then both adolescents and their parents will be invited to complete an online 'intake' survey. This reflects the importance of obtaining a 'multi-informant' perspective (34–36).

On completion of the online intake survey, adolescent participants will initiate the *EMA surveys*. They will be provided with both written and video instructions on how to take part, and offered on-boarding support via a video call with study staff. Participants will use their own smartphones where possible; however, to avoid selection biases and promote inclusion, study smartphones will be available. One week into the EMA period, they will be offered the option of scheduling a 'check-in'. An email helpline will also be available for participants to receive help on any issues they encounter.

EMA data collection will take place over a two-week period and involve 5 measures per day, all administered via a smartphone application. Four of these measures will be delivered at quasi-random intervals (random within four blocks) and include identical measures of momentary experiences. The 5th will be a brief 'daily diary' that captures concepts that vary over slightly longer timescales (e.g., sleep, medication use, physical activity). All measures will be designed to take less than 2 minutes as long questionnaires are associated with increased burden and lower compliance and our YPAG/PAG emphasised the importance of minimising burden (37).

At the end of the two-week EMA period, participants will complete an online *end-of study* survey. Here they will complete repeat measures of a subset of the questions administered in the original survey, as well as a brief set of questions capturing their participation experiences. Full questionnaires are provided in Supplementary Materials.

Measures

Adolescent measures

The measures that adolescents will complete over the course of the study are presented in **Table 1**. Full descriptions of the measures are provided in Supplemental Materials.

Parent measures

The measures that parents will complete over the course of the study are presented in **Table 2**. Full descriptions of the measures are provided in Supplemental Materials.

For peer review only

Table 1

Measures for adolescent surveys

Phase	Measures	Sources	No items
Intake			
	Demographics	Gender, Date of Birth, Ethnicity, Postcode	4
	Internalising problems	Revised Child Anxiety and Depression Scale (RCADS-11) (38)	11
	ADHD Strengths*	Newly developed measure	8
	Emotion dysregulation	Difficulties in Emotion Regulation Scale (DERS-16) (39)	16
	Emotion regulation strategy use	Emotion Regulation Questionnaire adapted for Children and Adolescents (ERQ-CA) (40)	10
	Peer problems and friendships	Social Relationships Questionnaire (SRQ) (41,42)	11
	Attachment security	Inventory of Parent and Peer Attachment (IPPA-10) (43)	10
	Quality of teacher-student relationships	Measure used in prior longitudinal study (44)	3
	Self-esteem	Rosenberg Self-Esteem questionnaire (45)	10
	Autistic traits	Autism Symptom SElf-ReporT (ASSERT) (46,47)	7
	Rejection sensitivity	Child Rejection Sensitivity Questionnaire (CRSQ) (48,49)	6
EMA			
	ADHD symptoms	Adapted from DSM criteria and prior ADHD EMA studies (3)	3
	Medication use	Has medication been taken since last assessment	1
	Emotions	Adapted from Positive Affect Negative Affect Schedule expanded (PANAS-X) (50)	6
	Emotion regulation strategies	Adapted version of the Emotion Regulation Questionnaire (51)	2
	Peer interactions	Newly developed measure	4
Daily Diary			
	Medication use	Whether and when medication was taken in last 24 hours	2
	Physical activity	Adapted from an open EMA item pool (52)	3
	Sleep	Abbreviated version of the Consensus Sleep Diary core items (53)	7
End			
	Internalising problems	Revised Child Anxiety and Depression Scale (RCADS-11) (38)	11
	Participation experiences	EMA and daily diary enjoyment, burden, and impact on routine; suggestions	4

Note. EMA, ecological momentary assessment; No, number. *Not completed by the control sample.

For peer review only

Table 2

Measures for parent surveys

Phase	Measures	Sources	No items
Intake			
	Demographics	Respondent Gender, Ethnicity, Education, Postcode, Free School Meals	5
	Child's ADHD or other diagnoses	Diagnosis Source, Diagnosis Age, Other Diagnoses	3
	Child's ADHD medication use	Medication Adherence Report Scale (54)	5
	Child's ADHD strengths*	Newly developed measure	8
	Child's ADHD, conduct and oppositional symptoms	Disruptive Behavior Disorder Rating Scale (DBD) (55)	45
	Child's internalising problems	Revised Child Anxiety and Depression Scale (RCADS-11) (38)	11
	Child's peer conflict and friendships	Social Relationships Questionnaire (SRQ) (41,42)	11
End			
	Child's ADHD, conduct and oppositional symptoms	Disruptive Behavior Disorder Rating Scale (DBD) (55)	45
	Child's internalising problems	Revised Child Anxiety and Depression Scale (RCADS-11) (38)	11
	Child's participation experiences	Study enjoyment and likelihood to participate again; suggestions	3

Note. No, number. *Not completed by parents of the control sample.

For peer review only

Piloting

Prior to the main data collection, the protocol will be piloted in a sample of n=10 adolescents (5 with and 5 without ADHD). Further modifications may be made to the protocol after piloting.

Statistical Procedure

Data will be analysed using techniques such as dynamic structural equation modelling (DSEM; Asparouhov et al., 2018; Asparouhov & Muthén, 2020; McNeish & Hamaker, 2020). For example, using two-level DSEM with participant random effects, it will be possible to derive individual-level indices of the mean levels, variability, and covariation of different concepts measured in the EMA. For example, we can derive indices of emotion regulation such as: variability in emotional state (lability), strength of relations between prior and subsequent emotional state (inertia), strength of relations between an event (e.g., a peer conflict) and emotional state (reactivity) and examine their links to peer, mental health, and behavioural issues, including as mediators of the links between ADHD symptoms and these outcomes. They can be studied alongside emotion regulation strategy use, resulting emotional states, and differences in these between adolescents with and without ADHD. Specific confirmatory analyses will be pre-registered and suitable multiple comparisons correction approaches selected as relevant.

Sample size justification

Our sample size (n=120 adolescents with ADHD and n=120 without) draws on several sample size determination approaches. It is partly based on a resource constraint approach, i.e., we will maximise the sample size attainable within the resources available (59). It is informed by Monte Carlo simulation studies using DSEM population models with varying effect sizes for the within- and between-person structures. These have suggested that a minimum of 50 respondents with 10 measurement points are needed to avoid small sample bias for random effects and to provide adequate coverage for effect sizes at the within- and between-person level that are likely to be of clinical importance (60,61). This is supplemented by our own simulations using population parameters derived from data gathered using similar designs in previous studies suggest that 100 respondents (50 per group) measured 50 times provides adequate coverage for realistic parameter estimates e.g.: https://osf.io/pvrma/. Taken together, our choice of n=120 per group balances feasibility with statistical precision and includes buffer for missing data and attrition.

Discussion

The current protocol describes the planned design for a combined traditional and EMA survey study (62) of the daily life manifestation and impacts of emotion regulation and peer issues among adolescents with and without ADHD. Building on previous evidence that emotion regulation and peer problems are common and inter-connected among youth with ADHD (1,15), we will seek to illuminate the most proximal targets in the daily lives of these youth to leverage in interventions. Adolescence is a critical transition period for emotion regulation and social development that may be particularly challenging for youth with ADHD (15). Reflecting the importance of involving those most affected by the research in mental health and neurodevelopmental research (63), the project has been and will continue to be informed by two panels of experts by experience, namely adolescents with ADHD and parents of adolescents with ADHD.

The study is motivated by a need for new data to inform more ecologically valid interventions that can provide effective improvements to adolescents' emotional and social

For peer review only

functioning in their daily lives (9). Though new, more integrative intervention approaches continue to be developed (e.g., Breaux & Langberg, 2020), the lack of attempts to address emotion regulation and social skills together and a lack of generalizability to real life functioning are two major critiques of existing supports for adolescents with ADHD (9). By gathering information about how emotion regulation and (potential) peer problems manifest, how they are linked and how they contribute to the development of co-occurring mental health or behavioural concerns, our findings can inform improvements to interventions to enhance their ecological validity.

Our findings may be particularly valuable for informing smartphone-based interventions (65). These show strong promise for adolescents with ADHD because smartphone use is highly embedded within the daily routines of young people, can allow the regular practice of skills within ecological context, and can be responsive to incoming data that, for example, may indicate high moments of risk for adverse outcomes (in 'ecological momentary interventions').Their scale-able and easily accessible format can also help make interventions more widely and more quickly available to the population of youth who may benefit from them. However, recent reviews of smartphone applications for ADHD have suggested that very few available applications are based on a robust evidence base (65). Further, mental health smartphone-based applications have been shown to be associated with low compliance (66) and are frequently abandoned after only a small number of uses (67). Our study can help to gain insights into the factors that can promote better engagement with smartphone-delivered interventions. This will be particularly important for adolescents with ADHD, who may find sustained engagement with interventions difficult, necessitating tailored protocols that, for example, provide immediate and high value rewards for compliance.

Our findings can also help accelerate progress in the application of EMA methodologies in populations of adolescents with ADHD. To date, despite their promise, very few such studies have been conducted (26,27). By providing a template for such research (which we will make openly available in the form of openly accessible study materials, including measures and analysis code), and documenting challenges, solutions, and benefits, this study can facilitate wider application of this technique.

Limitations

Though a large body of research has investigated optimal design features for EMA in general (37,68–70), the relative novelty of the approach in adolescents with ADHD (see e.g., Babinski & Welkie, 2020; Breaux et al., 2020 for notable exceptions) means that there is little past research to guide the selection of measures, sampling frequency and schedule, incentives, strategies to promote engagement, and other design features. More broadly, there remains a lack of attention paid to the development and validation of measures for EMA (72) and this means that there is little psychometric evidence underpinning available EMA measures of core constructs for the study such as emotion regulation and peer problems (e.g., Silk, 2019). Our study will thus help contribute to the development and validation of robust EMA measures but will not benefit from a strong pre-existing evidence base to guide which measures are likely to show the best psychometric properties. Finally, though EMA can overcome some of the reporting difficulties associated with traditional surveys (e.g., reporting biases and accuracies due to their reliance on retrospective recall), they are not invulnerable to self-report biases. For example, previous research has suggested that some adolescents with ADHD may be vulnerable to positive illusory biases that may result in a lack of awareness of difficulty and thus overly optimistic reporting of functioning (74).

For peer review only

Conclusions

EMA studies of the daily life functioning of adolescents with ADHD are needed to inform proximal intervention targets and more ecologically valid interventions for common and distressing outcomes experienced by this group, such as emotion dysregulation and peer problems. The current study outlines a protocol for a combined EMA and online survey study that can help meet this need and also accelerate progress in the application of EMA methodologies for illuminating the day-to-day manifestation and impacts of ADHD symptoms in the critical period of adolescence.

For peer review only

Acknowledgements

We are grateful to Chloe Bolota, Katie Dryburgh, Yi Yang, Zhuoni Xiao, Xinxin Zhu, and Ercan Ozdemir for their help in identifying and piloting measures used in this study.

Funding

The research described within this report was supported by a Medical Research Foundation (MRF) grant (Reference: MRF-001-0014-RG-MURR-C0932) and ALM was additionally supported by a Wellcome Trust Career Development Grant. The support of the MRF and Wellcome Trust is gratefully acknowledged.

Competing Interests

Luis Augusto Rohde has received grant or research support from, served as a consultant to, and served on the speakers' bureau of Abdiibrahim, Abbott, Aché, Bial, Medice, Novartis/Sandoz, Pfizer/Upjohn, and Shire/Takeda in the last three years. The ADHD and Juvenile Bipolar Disorder Outpatient Programs chaired by Dr Rohde have received unrestricted educational and research support from the following pharmaceutical companies in the last three years: Novartis/Sandoz and Shire/Takeda. Dr Rohde has received authorship royalties from Oxford Press and ArtMed.

Author Contributions

Conceptualisation: ALM; Funding acquisition: ALM AU SR TS LR IO JD LR ; Methodology: ALM JK LS IO MT; Writing – Original Draft: ALM; Writing – Review & Editing: ALM, LS, MT, TS, IO, JK, KW, JD, LR, AU, SR

References

1. Breaux R, Langberg JM, Swanson CS, Eadeh HM, Becker SP. Variability in positive and negative affect among adolescents with and without ADHD: Differential associations with functional outcomes. Journal of affective disorders. 2020;274:500–7.

2. Murray AL, Booth T, Eisner M, Auyeung B, Murray G, Ribeaud D. Sex differences in ADHD trajectories across childhood and adolescence. Developmental science. 2018;e12721.

3. Pedersen SL, Kennedy TM, Joseph HM, Riston SJ, Kipp HL, Molina BS. Real-world changes in adolescents' ADHD symptoms within the day and across school and non-school days. Journal of abnormal child psychology. 2020;48(12):1543–53.

4. Faraone SV, Rostain AL, Blader J, Busch B, Childress AC, Connor DF, et al. Practitioner Review: Emotional dysregulation in attention-deficit/hyperactivity disorder–implications for clinical recognition and intervention. Journal of Child Psychology and Psychiatry. 2019;60(2):133–50.

5. Antony EMA, Pihlajamäki MR, Speyer LG, Murray AL. Does emotion dysregulation mediate the association between ADHD symptoms and internalizing problems? A longitudinal within-person analysis in a large population-representative study. 2021;

For peer review only

6. Murray AL, Wong SC, Obsuth I, Rhodes S, Eisner M, Ribeaud D. An ecological momentary assessment study of the role of emotional dysregulation in co-occurring ADHD and internalising symptoms in adulthood. Journal of affective disorders. 2021;281:708–13.

7. Steinberg EA, Drabick DA. A developmental psychopathology perspective on ADHD and comorbid conditions: the role of emotion regulation. Child Psychiatry & Human Development. 2015;46(6):951–66.

8. Lenzi F, Cortese S, Harris J, Masi G. Pharmacotherapy of emotional dysregulation in adults with ADHD: A systematic review and meta-analysis. Neuroscience and Biobehavioral Reviews. 2018;359–67.

9. Morris S, Sheen J, Ling M, Foley D, Sciberras E. Interventions for adolescents with ADHD to improve peer social functioning: A systematic review and meta-analysis. Journal of attention disorders. 2021;25(10):1479–96.

10. Vacher C, Goujon A, Romo L, Purper-Ouakil D. Efficacy of psychosocial interventions for children with ADHD and emotion dysregulation: a systematic review. Psychiatry research. 2020;113151.

11. Shaw P, Stringaris A, Nigg J, Leibenluft E. Emotion dysregulation in attention deficit hyperactivity disorder. Focus. 2015;14(1):127–44.

12. Bunford N, Evans SW, Wymbs F. ADHD and emotion dysregulation among children and adolescents. Clinical child and family psychology review. 2015;18(3):185–217.

13. Graziano PA, Garcia A. Attention-deficit hyperactivity disorder and children's emotion dysregulation: A meta-analysis. Clinical psychology review. 2016;46:106–23.

14. Shaw P, Stringaris A, Nigg J, Leibenluft E. Emotion dysregulation in attention deficit hyperactivity disorder. American Journal of Psychiatry. 2014;171(3):276–93.

15. Bunford N, Evans SW, Langberg JM. Emotion dysregulation is associated with social impairment among young adolescents with ADHD. Journal of Attention Disorders. 2018;22(1):66–82.

16. Murray AL, Zych I, Ribeaud D, Eisner M. Developmental relations between ADHD symptoms and bullying perpetration and victimisation in adolescence. 2020;

17. Babinski DE, Kujawa A, Kessel EM, Arfer KB, Klein DN. Sensitivity to peer feedback in young adolescents with symptoms of ADHD: examination of neurophysiological and self-report measures. Journal of abnormal child psychology. 2019;47:605–17.

18. Lesnick J, Mendle J. Rejection sensitivity and negative urgency: A proposed framework of intersecting risk for peer stress. Developmental Review. 2021;62:100998.

19. Humphreys KL, Katz SJ, Lee SS, Hammen C, Brennan PA, Najman JM. The association of ADHD and depression: Mediation by peer problems and parent–child difficulties in two complementary samples. Journal of abnormal psychology. 2013;122(3):854.

20. Roy A, Hartman CA, Veenstra R, Oldehinkel AJ. Peer dislike and victimisation in pathways from ADHD symptoms to depression. European child & adolescent psychiatry. 2015;24(8):887–95.

For peer review only

21. Slaughter KE, Leaberry KD, Fogleman ND, Rosen PJ. Reactive and proactive aggression in children with and without ADHD and negative emotional lability. Social Development. 2019;

22. Yip VT, Ang RP, Ooi YP, Fung DS, Mehrotra K, Sung M, et al. The association between attention problems and internalizing and externalizing problems: The mediating role of peer problems. In: Child & Youth Care Forum. Springer; 2013. p. 571–84.

23. Russell MA, Gajos JM. Annual Research Review: Ecological momentary assessment studies in child psychology and psychiatry. Journal of Child Psychology and Psychiatry. 2020;61(3):376–94.

24. Speyer LG, Brown RH, Ribeaud D, Eisner M, Murray AL. The role of moment-to-moment dynamics of perceived stress and negative affect in co-occurring ADHD and internalising symptoms. Journal of Autism and Developmental Disorders. 2022;1–11.

25. Talty A, Speyer LG, Eisner M, Ribeaud D, Murray AL, Obsuth I. The role of student-teacher relationships in the association between adverse parenting and emotion dynamics–combining longitudinal and ecological momentary assessment data. 2022;

26. Koch ED, Moukhtarian TR, Skirrow C, Bozhilova N, Asherson P, Ebner-Priemer UW. Using e-diaries to investigate ADHD–State-of-the-art and the promising feature of just-in-time-adaptive interventions. Neuroscience & Biobehavioral Reviews. 2021;127:884–98.

27. Miguelez-Fernandez C, de Leon SJ, Baltasar-Tello I, Peñuelas-Calvo I, Barrigon ML, Capdevila AS, et al. Evaluating attention-deficit/hyperactivity disorder using ecological momentary assessment: a systematic review. ADHD Attention Deficit and Hyperactivity Disorders. 2018;10(4):247–65.

28. Lunsford-Avery JR, Krystal AD, Kollins SH. Sleep disturbances in adolescents with ADHD: A systematic review and framework for future research. Clinical psychology review. 2016;50:159–74.

29. Neudecker C, Mewes N, Reimers AK, Woll A. Exercise interventions in children and adolescents with ADHD: a systematic review. Journal of attention disorders. 2019;23(4):307–24.

30. Wylock JF, Borghini A, Slama H, Delvenne V. Child attachment and ADHD: a systematic review. European child & adolescent psychiatry. 2021;1–12.

31. Mahdi S, Viljoen M, Massuti R, Selb M, Almodayfer O, Karande S, et al. An international qualitative study of ability and disability in ADHD using the WHO-ICF framework. European child & adolescent psychiatry. 2017;26(10):1219–31.

32. Rong Y, Yang CJ, Jin Y, Wang Y. Prevalence of attention-deficit/hyperactivity disorder in individuals with autism spectrum disorder: a meta-analysis. Research in Autism Spectrum Disorders. 2021;83:101759.

33. Rosello R, Martinez-Raga J, Mira A, Pastor JC, Solmi M, Cortese S. Cognitive, social, and behavioral manifestations of the co-occurrence of autism spectrum disorder and attention-deficit/hyperactivity disorder: A systematic review. Autism. 2022;26(4):743–60.

34. De Los Reyes A. Strategic objectives for improving understanding of informant discrepancies in developmental psychopathology research. Development and psychopathology. 2013;25(3):669–82.

For peer review only

35. Murray AL, Booth T, Ribeaud D, Eisner M. Disagreeing about development: An analysis of parent-teacher agreement in ADHD symptom trajectories across the elementary school years. International journal of methods in psychiatric research. 2018;e1723.

36. Murray AL, Ribeaud D, Eisner M, Murray G, McKenzie K. Should We Subtype ADHD According to the Context in Which Symptoms Occur? Criterion Validity of Recognising Context-Based ADHD Presentations. Child Psychiatry & Human Development. 2018;1–13.

37. Eisele G, Vachon H, Lafit G, Kuppens P, Houben M, Myin-Germeys I, et al. The effects of sampling frequency and questionnaire length on perceived burden, compliance, and careless responding in experience sampling data in a student population. Assessment. 2020;1073191120957102.

38. Radez J, Waite P, Chorpita B, Creswell C, Orchard F, Percy R, et al. Using the 11-item Version of the RCADS to Identify Anxiety and Depressive Disorders in Adolescents. Research on child and adolescent psychopathology. 2021;49:1241–57.

39. Bjureberg J, Ljótsson B, Tull MT, Hedman E, Sahlin H, Lundh LG, et al. Development and validation of a brief version of the difficulties in emotion regulation scale: The DERS-16. J Psychopathol Behav Assess. 2016 Jun;38(2):284–96.

40. Gullone E, Taffe J. The Emotion Regulation Questionnaire for Children and Adolescents (ERQ–CA): A psychometric evaluation. Psychological Assessment. 2012;24:409–17.

41. Hinshaw SP, Owens EB, Sami N, Fargeon S. Prospective follow-up of girls with attention-deficit/hyperactivity disorder into adolescence: Evidence for continuing cross-domain impairment. Journal of consulting and clinical psychology. 2006;74(3):489.

42. Owens EB, Hinshaw SP. Childhood conduct problems and young adult outcomes among women with childhood attention-deficit/hyperactivity disorder (ADHD). Journal of abnormal psychology. 2016;125(2):220.

43. Buist KL, Deković M, Meeus WH, van Aken MA. Attachment in adolescence: A social relations model analysis. Journal of Adolescent research. 2004;19(6):826–50.

44. Obsuth I, Murray AL, Knoll M, Ribeaud D, Eisner M. Teacher-student relationships in childhood as a protective factor against adolescent delinquency up to age 17: A propensity score matching approach. Crime & Delinquency. 2021;00111287211014153.

45. Rosenberg M. Rosenberg self-esteem scale (SES). Society and the adolescent self-image. 1965;

46. Gillberg C, Gillberg C, Råstam M, Wentz E. The Asperger Syndrome (and high-functioning autism) Diagnostic Interview (ASDI): a preliminary study of a new structured clinical interview. Autism. 2001;5(1):57–66.

47. Posserud MB, Breivik K, Gillberg C, Lundervold AJ. ASSERT–the autism symptom SElf-ReporT for adolescents and adults: bifactor analysis and validation in a large adolescent population. Research in Developmental Disabilities. 2013;34(12):4495–503.

48. Bondü R, Esser G. Justice and rejection sensitivity in children and adolescents with ADHD symptoms. European Child & Adolescent Psychiatry. 2015;24:185–98.

For peer review only

49. Downey G, Lebolt A, Rincón C, Freitas AL. Rejection sensitivity and children's interpersonal difficulties. Child development. 1998;69(4):1074–91.

50. Watson D, Clark LA. The PANAS-X: Manual for the positive and negative affect schedule-expanded form. 1999;

51. Gross JJ, John OP. Individual differences in two emotion regulation processes: implications for affect, relationships, and well-being. Journal of personality and social psychology. 2003;85(2):348.

52. Klein A, Clucas J, Krishnakumar A, Ghosh SS, Van Auken W, Thonet B, et al. Remote digital psychiatry for mobile mental health assessment and therapy: MindLogger platform development study. Journal of medical Internet research. 2021;23(11):e22369.

53. Carney CE, Buysse DJ, Ancoli-Israel S, Edinger JD, Krystal AD, Lichstein KL, et al. The consensus sleep diary: standardizing prospective sleep self-monitoring. Sleep. 2012;35(2):287–302.

54. Chan AHY, Horne R, Hankins M, Chisari C. The Medication Adherence Report Scale: A measurement tool for eliciting patients' reports of nonadherence. Br J Clin Pharmacol. 2020 Jul;86(7):1281–8.

55. Pelham Jr WE, Gnagy EM, Greenslade KE, Milich R. Teacher ratings of DSM-III-R symptoms for the disruptive behavior disorders. Journal of the American Academy of Child & Adolescent Psychiatry. 1992;31(2):210–8.

56. Asparouhov T, Hamaker EL, Muthén B. Dynamic structural equation models. Structural Equation Modeling: A Multidisciplinary Journal. 2018;25(3):359–88.

57. Asparouhov T, Muthén B. Comparison of models for the analysis of intensive longitudinal data. Structural Equation Modeling: A Multidisciplinary Journal. 2020;27(2):275–97.

58. McNeish D, Hamaker EL. A primer on two-level dynamic structural equation models for intensive longitudinal data in Mplus. Psychological methods. 2020;25(5):610.

59. Lakens D. Sample size justification. Collabra: Psychology. 2022;8(1):33267.

60. McNeish D. Two-level dynamic structural equation models with small samples. Structural equation modeling: a multidisciplinary journal. 2019;26(6):948–66.

61. Schultzberg M, Muthén B. Number of subjects and time points needed for multilevel time-series analysis: A simulation study of dynamic structural equation modeling. Structural Equation Modeling: A Multidisciplinary Journal. 2018;25(4):495–515.

62. Murray AL, Speyer LG, Brown R, Zhu X, Yang Y, Eisner M, et al. Advancing multi-timeframe developmental research through combining long-term cohort and ecological momentary assessment studies: The Decades-to-minutes (D2M) study [Internet]. PsyArXiv; 2022 [cited 2022 Apr 2]. Available from: https://psyarxiv.com/yqust/

63. Pavarini G, Lorimer J, Manzini A, Goundrey-Smith E, Singh I. Co-producing research with youth: The NeurOx young people's advisory group model. Health Expectations. 2019;22(4):743–51.

For peer review only

64. Breaux R, Langberg JM. Development and refinement of the RELAX intervention, an intervention targeting emotion dysregulation and interpersonal conflict in adolescents with ADHD: Results from a pilot study. Evidence-Based Practice in Child and Adolescent Mental Health. 2020;5(2):147–63.

65. Păsărelu CR, Andersson G, Dobrean A. Attention-deficit/hyperactivity disorder mobile apps: A systematic review. International journal of medical informatics. 2020;138:104133.

66. Melcher J, Patel S, Scheuer L, Hays R, Torous J. Assessing Engagement Features in an Observational Study of Mental Health Apps in College Students. Psychiatry Research. 2022;114470.

67. Baumel A, Muench F, Edan S, Kane JM. Objective user engagement with mental health apps: systematic search and panel-based usage analysis. Journal of medical Internet research. 2019;21(9):e14567.

68. Rintala A, Wampers M, Myin-Germeys I, Viechtbauer W. Momentary predictors of compliance in studies using the experience sampling method. Psychiatry research. 2020;286:112896.

69. Smyth JM, Jones DR, Wen CK, Materia FT, Schneider S, Stone A. Influence of ecological momentary assessment study design features on reported willingness to participate and perceptions of potential research studies: an experimental study. BMJ open. 2021;11(7):e049154.

70. Wrzus C, Neubauer AB. Ecological Momentary Assessment: A Meta-Analysis on Designs, Samples, and Compliance Across Research Fields. Assessment. 2021;10731911211067538.

71. Babinski DE, Welkie J. Feasibility of ecological momentary assessment of negative emotion in girls with ADHD: A pilot study. Psychological reports. 2020;123(4):1027–43.

72. Murray AL, Eisner M, Ribeaud D, Booth T. Validation of a brief measure of aggression for ecological momentary assessment research: the Aggression-ES-A. Assessment. 2020;1073191120976851.

73. Silk JS. Context and dynamics: The new frontier for developmental research on emotion regulation. Developmental psychology. 2019;55(9):2009.

74. Bourchtein E, Langberg JM, Owens JS, Evans SW, Perera RA. Is the positive illusory bias common in young adolescents with ADHD? A fresh look at prevalence and stability using latent profile and transition analyses. Journal of abnormal child psychology. 2017;45(6):1063–75.

Word Count

3,374

Figure Legend

Figure 1: Summary of data collection schedule

For peer review only

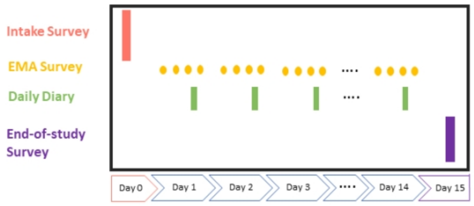

Figure 1: Summary of data collection schedule

338x190mm (96 x 96 DPI)

Supplementary Materials

S1: Description of Measures

Adolescents intake survey

Gender will be measured using an item with options ‘male’, ‘female’, ‘non-binary’, ‘gender fluid’, ‘questioning’, ‘prefer to self-describe’ and ‘prefer not to say’.

Date of birth will be collected and used to derive age at time of assessment.

Ethnicity will be measured using a set of items designed to be inclusive of different ethnic identities, with the additional options of “Prefer not to say” and “Not listed, please specify.”

Socioeconomic status (SES) will be estimated from respondent’s home postcodes and used to derive the (Scottish) index of multiple deprivation [(S)IMD].

Internalising problems, will be measured using an 11-item (6 anxiety items; 5 depression items) self-report *Revised Child Anxiety and Depression* Scale (38) . Previous research has suggest that the scale scores have favourable psychometric properties in adolescents (38)

ADHD strengths will be measured using a newly developed 8-item measure produced by the team in consultation with the youth and parent advisory groups. Items were written for strengths associated with ADHD that included hyperfocus, creativity, energy, interests, and problem-solving. While previous work has identified perceived strengths associated with ADHD (31), no suitable validated measure of ADHD strengths in this age group currently exists. Full details of the scale development and validation will be documented in future publications. This will not be completed by the control sample.

Emotion dysregulation will be measured using *Difficulties in Emotion Regulation Scale* (39)*,*16 item version (DERS-16 Bjureberg et al., 2016). It measures lack of emotional clarity, difficulties in engaging in goal-directed behaviour, impulse control difficulties, limited access to effective emotion regulation strategies, and non-acceptance of emotional responses. Previous research has suggested the scale scores has favourable psychometric properties in adolescents with mental health issues (e.g., Charak et al., 2019).

Emotion regulation strategy use will be measured using the *Emotion Regulation Questionnaire* (42) adapted for children and adolescents (ERQ-CA Gullone & Taffe, 2012). It measures cognitive re-appraisal and suppression in two corresponding subscales of 6 and 4 items respectively. Previous research has suggested that the scale scores have favourable psychometric properties in adolescent samples (43).

Peer problems and friendships will be measured using the Social Relationships Questionnaire (SRQ). This is an 11-item measure of adolescent peer conflict and friendships initially developed for parent-report but adapted for self-report in the current study. Responses are recorded on a 4-point Likert-type scale from *not true* to *very true*. The parent-report version of this measure has been used in prior studies of conduct and peer problems in adolescents with ADHD (44,45).

Attachment security will be measured using the Inventory of Parent and Peer Attachment [IPPA-10;(46)]. This is a 10-item scale measuring respondents’ perceptions of trust, communication and alienation in their relationship with a primary caregiver. The scale also yields an overall attachment security score.

The *quality of teacher-student relationships* will be assessed by rating three statements: ‘I get along with my teacher’; ‘The teacher is fair to me’; and ‘The teacher supports me’ on a 4-point Likert scale indicating the level of agreement with each

For peer review only

(*completely untrue* to *completely* true). The questions have been used in another longitudinal study and have shown good reliability (alpha = .79) and validity (47).

Self-esteem will be measured using the Rosenberg Self-Esteem questionnaire (48). The scale includes 10 items (5 positively and 5 negatively worded) and an overall self-esteem score can be obtained by (after reverse coding as relevant) summation of the item responses.

Autistic traits will be measured using the 7-item Autism Symptom SElf-ReporT (ASSERT). Derived from a structured diagnostic interview (49), the scale measures social difficulties (4 items) and repetitive behaviour and circumscribed interests (3 items) (50).

Rejection sensitivity will be measured using a 6-item version of the *Child Rejection Sensitivity Questionnaire* (51,52). The scale presents respondents with 6 different scenarios that could result in rejection and for each they are asked to rate how anxious and angry they would feel prior to the outcome and how likely rejection would be. Anxious and anger rejection sensitivity are calculated by multiplication of the likelihood of rejection and corresponding anger/anxious scores for each scenario. Scores for each scenario can be summed to provide overall angry and anxious rejection scores.

Parents intake survey

Respondent gender will be measured using the same item as in the adolescent self-report questionnaire.

Respondent ethnicity will be measured using a set of items designed to be inclusive of different ethnic identities, with the additional options of “Prefer not to say” and “Not listed, please specify.”

Respondent education will be measured by asking respondents “what is the highest degree or level of school you have completed?” A set of items designed to be representative of the educational qualifications throughout the UK are provided with the additional options of “Prefer not to say” and “Not listed, please specify.”

Socioeconomic status will be estimated using SIMD and IMD based on postal code. Parents will also be asked whether their child is eligible for free school meals.

Child’s ADHD Diagnosis and other mental health/neurodevelopmental diagnoses. Parents will be asked for information on who diagnosed their child with ADHD as well as the child’s age at diagnosis. Further, parents will be asked whether their child has any other neurodevelopmental or mental health condition. They can choose from a list or specify other diagnoses in a free text field.

Child’s ADHD medication use. Parents will be asked which ADHD medications their child uses, dosage, and adherence (48). The latter will be measured using the 5-item Medication Adherence Report Scale (MARS, Chan et al., 2020).

Child’s ADHD strengths will be measured using the parent-report version of the above-described ADHD strengths measure (see self-report measures above). They will not be completed by parents of the control sample.

Child’s ADHD, conduct and oppositional symptoms will be measured using the *Disruptive Behavior Disorder Rating Scale* (49). The ADHD subscale includes 9 items measuring symptoms of inattention as well as 9 items measuring symptoms of hyperactivity and impulsivity, scored on a 4-point scale from *not at all* to *very much.* The scale was originally developed under DSM-III-R criteria, however, the symptoms assessed through this measure map on well to criteria outlined in DSM-V (American Psychiatric Association, 2013).

Child’s internalising problems will be measured with a parent-report version of the RCADS-11.

For peer review only

Child's peer conflict and friendships will be measured with the parent-report version of the SRQ.

Ecological momentary assessment measures

ADHD symptoms will be measured using a 3-item momentary symptoms scale developed by the team with reference to DSM criteria. The items are similar to EMA items used in previous EMA ADHD studies (e.g., Pedersen et al., 2020) and cover inattention, hyperactivity, and impulsivity.

Medication use will be measured by asking whether the young person has taken their medication since the last assessment.

Emotions will be measured using abbreviated versions of the Positive Affect Negative Affect Schedule expanded version (PANAS-X;Watson & Clark, 1999). Affect items were selected based on covering a wide range of emotions and being most relevant to ADHD and associated impairments. These will be used to derive indices of emotional lability, inertia, and reactivity within DSEM (53)

Emotion regulation strategies will be measured using an EMA-adapted version of the Emotion Regulation Questionnaire (54). Two items will be used to measure: suppression, and cognitive re-appraisal to experience less negative affect.

Peer interactions will be measured using a brief set of 4 items capturing experiences of *acceptance*, *victimisation, aggression*, and *positive peer interactions.* These items were developed by the team, drawing on previous EMA measures of social experiences (55) and considering common social difficulties among adolescents with ADHD.

Daily diary measures

Medication use will be measured using a two-part item asking whether and when the young person took ADHD medication in the last 24 hours.

Physical activity will be measured using three items asking respondents to report on the amount and intensity of physical activity undertaken in the last 24 hours adapted from an open EMA item pool (56).

Sleep will be measured using an abbreviated version of the Consensus Sleep Diary core items (57).

Adolescents end-of-survey measure

The adolescent end-of-survey questionnaire will be used to re-assess *anxiety and depression* with the RCADS-11. To inform the further development of EMA methodology with adolescents with ADHD, we will ask a brief set of items on their *participation experiences*, including the perceived burden of the EMA and daily diaries, how *enjoyable* the EMA was, how much it changed their *routine*. We will also include an open text field soliciting views on how to improve EMA data collections from a participant's perspective.

Parents end-of-survey measures

The parents end-of survey questionnaire will be used to re-assess their child's emotional and behavioural problems using the RCADS-11 and DBD. It will also measure parents' perspective on the burden of the EMA and daily diary studies. We will also include

For peer review only

an open text field soliciting views on how to improve EMA data collections from a participant’s perspective.

For peer review only

S2: Adolescents intake online survey

Gender Which of these best describes your gender?

- ☐ Male
- ☐ Female
- ☐ Non-binary
- ☐ Gender fluid
- ☐ Questioning
- ☐ I prefer to self-describe: ____________________________
- ☐ Prefer not to say

Age What is your date of birth (day/month/year)?

____

Ethnicity Which of the following most accurately describes you? Please select all that apply.

- ☐ Bangladeshi
- ☐ Pakistani
- ☐ Indian
- ☐ Chinese
- ☐ Any other Asian background
- ☐ Caribbean
- ☐ African
- ☐ Any other Black, Black British, or Caribbean background
- ☐ White and Black Caribbean
- ☐ White and Black African
- ☐ White and Asian
- ☐ Any other Mixed or multiple ethnic background
- ☐ English, Welsh, Scottish, Northern Irish, or British
- ☐ Irish
- ☐ Gypsy or Irish Traveller
- ☐ Roma
- ☐ Any other White background
- ☐ Arab
- ☐ Prefer not to say
- ☐ Not listed, please specify: ____________________________

Postcode What is your postcode?

____

For peer review only

RCADS-11 Please select the word that shows how often each of these things happened to you over the past two weeks. There are no right or wrong answers.

	Never	Sometimes	Often	Always
I have trouble going to school in the mornings because I feel nervous or afraid	○	○	○	○
I have no energy for things	○	○	○	○
I worry when I go to bed at night	○	○	○	○
I worry about what is going to happen	○	○	○	○
Nothing is much fun anymore	○	○	○	○
All of a sudden I feel really scared for no reason at all	○	○	○	○
I feel worthless	○	○	○	○
I feel sad or empty	○	○	○	○
When I have a problem, my heart beats really fast	○	○	○	○
I am tired a lot	○	○	○	○
I worry I might look foolish	○	○	○	○

For peer review only

Strengths For each item, please select the response that is most true for you.

	Strongly Agree	Agree	Neither Disagree nor Agree	Disagree	Strongly Disagree
I am able to focus really well on the things I am interested in	○	○	○	○	○
I have lots of energy to get things done	○	○	○	○	○
I have tons of ideas	○	○	○	○	○
I am creative	○	○	○	○	○
I have a warm personality	○	○	○	○	○
I am full of enthusiasm	○	○	○	○	○
I am good at coming up with solutions to problems	○	○	○	○	○
I find it easy to develop new interests (e.g., hobbies)	○	○	○	○	○

For peer review only

DERS-16 Please select the response that is most true for you.

	Almost Never	Sometimes	About half the time	Most of the time	Almost always
I have difficulty making sense out of my feelings	○	○	○	○	○
I am confused about how I feel	○	○	○	○	○
When I'm upset, I have difficulty getting work done	○	○	○	○	○
When I'm upset, I become out of control	○	○	○	○	○
When I'm upset, I believe that I will remain that way for a long time	○	○	○	○	○
When I'm upset, I believe that I'll end up feeling very depressed	○	○	○	○	○
When I'm upset, I have difficulty focusing on other things	○	○	○	○	○
When I'm upset, I feel out of control	○	○	○	○	○
When I'm upset, I feel ashamed with myself for feeling that way	○	○	○	○	○
When I'm upset, I feel like I am weak	○	○	○	○	○
When I'm upset, I have difficulty controlling my behaviours	○	○	○	○	○
When I'm upset, I believe there is nothing I can do to make myself feel better	○	○	○	○	○
When I'm upset, I become irritated with myself for feeling that way	○	○	○	○	○
When I'm upset, I start to feel very bad about myself	○	○	○	○	○

For peer review only

When I'm upset, I have difficulty thinking about anything else	○	○	○	○	○
When I'm upset, my emotions feel overwhelming	○	○	○	○	○

For peer review only

ERQ-CA We would now like to ask you some questions about your emotional life. For each item, please select the response that is most true for you.

	Strongly Disagree	Disagree	Half and Half	Agree	Strongly Agree
When I want to feel happier, I think about something different	○	○	○	○	○
I keep my feelings to myself	○	○	○	○	○
When I want to feel less bad (e.g., sad, angry, or worried), I think about something different	○	○	○	○	○
When I am feeling happy, I am careful not to show it	○	○	○	○	○
When I'm worried about something, I make myself think about it in a way that helps me feel better	○	○	○	○	○
I control my feelings by not showing them	○	○	○	○	○
When I want to feel happier about something, I change the way I'm thinking about it	○	○	○	○	○
I control my feelings about things by changing the way I think about them	○	○	○	○	○
When I'm feeling bad (e.g. sad, angry, or worried), I am careful not to show it	○	○	○	○	○
When I want to feel less bad (e.g. sad, angry, or worried) about something, I change the way I'm thinking about it	○	○	○	○	○

For peer review only

SRQ We would like to learn more about your friendships. Please indicate how much each of the following statements applies to you.

	Not True	Sort of True	Mostly True	Very True
You have a "best friend" that you feel close to	○	○	○	○

If "Mostly True" or "Very True" selected display the following questions:

	Not True	Sort of True	Mostly True	Very True
This relationship is positive and beneficial for you (affectionate, supportive, intimate)	○	○	○	○
This relationship is negative for you (high conflict, aggression, betrayal, competitive)	○	○	○	○

Display following questions to all participants:

	Not True	Sort of True	Mostly True	Very True
You have a group of friends at school that you hang around with	○	○	○	○
You have a group of friends at home that you like to play with	○	○	○	○
You have always had problems with friendships	○	○	○	○
You seem to have fewer friends as you get older	○	○	○	○
You have difficulty making new friends	○	○	○	○
You have difficulty keeping the friends that you make	○	○	○	○
Your friend(s) are similar to you in the way they act and the things they like	○	○	○	○

For peer review only

You have problems with other people your age because you tease them	○	○	○	○
You have problems with other people your age because you get teased by them	○	○	○	○
You have difficulty responding appropriately to teasing	○	○	○	○

For peer review only

Caregiver What relationship does the person taking care of you most of the time (i.e. your primary caregiver) have to you?

- ☐ Mother
- ☐ Father
- ☐ Other, please specify: ___________________________
- ☐ Prefer not to say

IPPA-10 For each item, please select the response that is most true for you. Please give your answers thinking about the person who takes care of you most of the time (i.e. your primary caregiver).

	Very Untrue	Untrue	Sometimes Untrue	True	Very True
I don't get enough attention from my primary caregiver.	○	○	○	○	○
My primary caregiver accepts me the way I am.	○	○	○	○	○
When my primary caregiver knows something is bothering me, he/she will ask me about it.	○	○	○	○	○
I tell my primary caregiver about my problems and worries.	○	○	○	○	○
I get easily upset with my primary caregiver.	○	○	○	○	○
My primary caregiver respects my feelings.	○	○	○	○	○
I get mad at my primary caregiver.	○	○	○	○	○
My primary caregiver helps me to understand myself better.	○	○	○	○	○
When I talk about my problems with my primary caregiver, I feel ashamed or stupid.	○	○	○	○	○
When I get mad about something, my primary caregiver tries to understand that.	○	○	○	○	○

For peer review only

Teacher For each item, please select the response that is most true for you. Please give your answers thinking about the teacher who knows you best (e.g., your registration/form teacher).

	Completely Untrue	Mostly Untrue	Mostly True	Completely True
I get along with my teacher	○	○	○	○
My teacher is fair to me	○	○	○	○
My teacher supports me	○	○	○	○

For peer review only

Self-Esteem Below is a list of statements dealing with your general feelings about yourself. Please indicate how strongly you agree or disagree with each statement.

	Strongly Agree	Agree	Disagree	Strongly Disagree
On the whole, I am satisfied with myself	○	○	○	○
At times I think I am no good at all	○	○	○	○
I feel that I have a number of good qualities	○	○	○	○
I am able to do things as well as most other people	○	○	○	○
I feel I do not have much to be proud of	○	○	○	○
I certainly feel useless at times	○	○	○	○
I feel that I'm a person of worth, at least on an equal plane with others	○	○	○	○
I wish I could have more respect for myself	○	○	○	○
All in all, I am inclined to feel that I am a failure	○	○	○	○
I take a positive attitude toward myself	○	○	○	○

For peer review only

ASSERT Below is a list of statements dealing with your general feelings about yourself. Please indicate how strongly you agree or disagree with each statement.

	Not True	Somewhat True	Certainly True
Do you find it difficult to socialize with, or to get in touch with people, especially people your own age?	○	○	○
Do you prefer to be alone rather than being together with other people?	○	○	○
Do you have difficulties perceiving social cues?	○	○	○
Do other people tell you that your behavior or your emotional responses are inappropriate or hurtful?	○	○	○
Do you have a strong interest or hobby that absorbs so much of your time that it hampers other activities?	○	○	○
Do you or do other people feel that you have very set routines or that you are very immersed in your own interests?	○	○	○
Do you or do other people feel that you impose your routines or interests on others?	○	○	○

For peer review only

CRSQ Please imagine yourself in each of the following situations described here and select how you would feel in each.

Imagine you had a really bad fight the other day with a friend. Now you have a serious problem and you wish you had your friend to talk to. You decide to wait for your friend after class and talk with him/her. You wonder if your friend will want to talk to you.

1a How NERVOUS would you feel, RIGHT THEN, about whether or not your friend will want to talk to you and listen to your problem?

- ☐ not nervous
- ☐ 2
- ☐ 3
- ☐ 4
- ☐ 5
- ☐ very, very nervous

1b How MAD would you feel, RIGHT THEN, about whether or not your friend will want to talk to you and listen to your problem?

- ☐ not mad
- ☐ 2
- ☐ 3
- ☐ 4
- ☐ 5
- ☐ very, very mad

1c Do you think he/she will want to talk to you and listen to your problem?

- ☐ Yes
- ☐ 2
- ☐ 3
- ☐ 4
- ☐ 5
- ☐ No

Imagine that a famous person is coming to visit your school. Your teacher is going to pick five kids to meet this person. You wonder if she will choose you

2a How NERVOUS would you feel, RIGHT THEN, about whether or not the teacher will choose you?

- ☐ not nervous
- ☐ 2
- ☐ 3
- ☐ 4
- ☐ 5
- ☐ very, very nervous

For peer review only

2b How MAD would you feel, RIGHT THEN, about whether or not the teacher will choose you?

- ☐ not mad
- ☐ 2
- ☐ 3
- ☐ 4
- ☐ 5
- ☐ very, very mad

2c Do you think the teacher will choose YOU to meet the special guest?

- ☐ Yes
- ☐ 2
- ☐ 3
- ☐ 4
- ☐ 5
- ☐ No

Now imagine that you're back in class. Your teacher asks for a volunteer to help plan a party for your class. Lots of kids raise their hands so you wonder if the teacher will choose YOU.

3a How NERVOUS would you feel, RIGHT THEN, about whether or not the teacher will choose you?

- ☐ not nervous
- ☐ 2
- ☐ 3
- ☐ 4
- ☐ 5
- ☐ very, very nervous

3b How MAD would you feel, RIGHT THEN, about whether or not the teacher will choose you?

- ☐ not mad
- ☐ 2
- ☐ 3
- ☐ 4
- ☐ 5
- ☐ very, very mad

3c Do you think the teacher will choose YOU?

- ☐ Yes
- ☐ 2
- ☐ 3
- ☐ 4
- ☐ 5
- ☐ No

For peer review only

Imagine it's Saturday and you're carrying groceries home for your family. It is raining hard and you want to get home FAST. Suddenly, the paper bag you are carrying rips. All your food tumbles to the ground. You look up and see a couple of kids from your class walking quickly. You wonder if they will stop and help you

4a How NERVOUS would you feel, RIGHT THEN, about whether or not those kids will want to stop and help you?

- ☐ not nervous
- ☐ 2
- ☐ 3
- ☐ 4
- ☐ 5
- ☐ very, very nervous

4b How MAD would you feel, RIGHT THEN, about whether or not those kids will want to stop and help you?

- ☐ not mad
- ☐ 2
- ☐ 3
- ☐ 4
- ☐ 5
- ☐ very, very mad

4c Do you think they will offer to help you?

- ☐ Yes
- ☐ 2
- ☐ 3
- ☐ 4
- ☐ 5
- ☐ No

Pretend you have moved and you are going to a different school. In this school, the teacher lets the kids in the class take home a video game to play with on the weekend. Every week so far, you have watched someone else take it home. You decide to ask the teacher if YOU can take home the video game this time. You wonder if she will let you have it

5a How NERVOUS would you feel, RIGHT THEN, about whether or not the teacher will let you take the video game home this time?

- ☐ not nervous
- ☐ 2
- ☐ 3
- ☐ 4
- ☐ 5
- ☐ very, very nervous

For peer review only

5b How MAD would you feel, RIGHT THEN, about whether or not the teacher will let you take the video game home this time?

- not mad
- 2
- 3
- 4
- 5
- very, very mad

5c Do you think the teacher is going to let you take home the video game this time?

- Yes
- 2
- 3
- 4
- 5
- No

Imagine you're back in your classroom, and everyone is splitting up into groups to work on a special project together. You sit there and watch lots of other kids getting picked. As you wait, you wonder if the kids will want you for their group.

6a How NERVOUS would you feel, RIGHT THEN, about whether or not they will choose you?

- not nervous
- 2
- 3
- 4
- 5
- very, very nervous

6b How MAD would you feel, RIGHT THEN, about whether or not they will choose you?

- not mad
- 2
- 3
- 4
- 5
- very, very mad

6c Do you think the the kids in your class will choose you for their group?

- Yes
- 2
- 3
- 4
- 5
- No

For peer review only

S3: EMA and Daily Diary measures

EMA Measures

ADHD symptoms (inattention, hyperactivity, impulsivity)

I couldn't concentrate

Strongly agree	Agree	Neither agree nor disagree	Disagree	Strongly disagree

I acted before thinking

Strongly agree	Agree	Neither agree nor disagree	Disagree	Strongly disagree

I was hyperactive

Strongly agree	Agree	Neither agree nor disagree	Disagree	Strongly disagree

Emotions (PANAS)

In the last 30 minutes I felt…

Excited

Very slightly or not at all	A little	Moderately	Quite a bit	Extremely

Upset

Very slightly or not at all	A little	Moderately	Quite a bit	Extremely

Determined

Very slightly or not at all	A little	Moderately	Quite a bit	Extremely

Enthusiastic

Very slightly or not at all	A little	Moderately	Quite a bit	Extremely

Ashamed

Very slightly or not at all	A little	Moderately	Quite a bit	Extremely

Nervous

Very slightly or not at all	A little	Moderately	Quite a bit	Extremely

For peer review only

Emotion regulation strategy use

In the last 30 minutes, I…

Kept my emotions to myself

Strongly Disagree	Disagree	Neither agree nor disagree	Agree	Strongly Agree

When I wanted to feel less negative emotion (such as sadness or anger), I changed what I was thinking about

Strongly Disagree	Disagree	Neither agree nor disagree	Agree	Strongly Agree

Peer interactions

In relation to my peers (other adolescents my age) in the last 30 minutes, I…

Felt accepted

Strongly Disagree	Disagree	Neither agree nor disagree	Agree	Strongly Agree

Was teased

Strongly Disagree	Disagree	Neither agree nor disagree	Agree	Strongly Agree

Got into an argument

Strongly Disagree	Disagree	Neither agree nor disagree	Agree	Strongly Agree

I enjoyed interacting (online or in person) with others my age.

Strongly Disagree	Disagree	Neither agree nor disagree	Agree	Strongly Agree

For peer review only

Daily Diary Measures

Physical activity

How many minutes did you do vigorous activities including intensive sports or exercise (such as running or fast cycling) or intensive physical work (such as heavy lifting or digging)?

How many minutes did you do moderate activities (activities that make you breathe somewhat harder than usual such as playing tennis, bicycling, carrying light loads)?

How many minutes did you do light activities (activities that may not make you breathe somewhat harder than usual such as walking, climbing stairs, routine household chores, etc.)?

Medication use

Have you taken ADHD medication in the last 24 hours?

Sleep (consensus sleep scale core items)

What time did you get into bed last night?

What time did you try to go to sleep?

How long did it take you to fall asleep (in minutes)?

How many times did you wake up, not counting your final awakening?

In total, how long did these awakenings last (in minutes)?

What time was your final awakening?

For peer review only

How would you rate quality of your sleep?

Very poor	Poor	Fair	Good	Very Good

Comments (if applicable)

For peer review only

S4: Adolescents end-of-study survey

RCADS-11 Please select the word that shows how often each of these things happened to you over the past two weeks. There are no right or wrong answers.

	Never	Sometimes	Often	Always
I have trouble going to school in the mornings because I feel nervous or afraid				
I have no energy for things				
I worry when I go to bed at night				
I worry about what is going to happen				
Nothing is much fun anymore				
All of a sudden I feel really scared for no reason at all				
I feel worthless				
I feel sad or empty				
When I have a problem, my heart beats really fast				
I am tired a lot				
I worry I might look foolish				

For peer review only

EMA feedback We would love to know how taking part in the smart-phone based surveys was for you. Please select the response that is most true for you thinking about your experiences of completing the smartphone-based surveys.

	Almost never	Sometimes	About half the time	Most of the time	Almost always
I found doing the smartphone surveys fun	○	○	○	○	○
I found doing the smartphone surveys hard work	○	○	○	○	○
Doing the smartphone surveys disrupted my routine	○	○	○	○	○

Suggestions Do you have thoughts or suggestions on how the smartphone surveys could be improved?

____

For peer review only

S5: Parents online intake survey

Gender Which of these best describes your gender?

- ☐ Male
- ☐ Female
- ☐ Non-binary
- ☐ Gender fluid
- ☐ Questioning
- ☐ I prefer to self-describe: ____________________________
- ☐ Prefer not to say

Age What is your date of birth (day/month/year)?

____

Ethnicity Which of the following most accurately describes you? Please select all that apply.

- ☐ Bangladeshi
- ☐ Pakistani
- ☐ Indian
- ☐ Chinese
- ☐ Any other Asian background
- ☐ Caribbean
- ☐ African
- ☐ Any other Black, Black British, or Caribbean background
- ☐ White and Black Caribbean
- ☐ White and Black African
- ☐ White and Asian
- ☐ Any other Mixed or multiple ethnic background
- ☐ English, Welsh, Scottish, Northern Irish, or British
- ☐ Irish
- ☐ Gypsy or Irish Traveller
- ☐ Roma
- ☐ Any other White background
- ☐ Arab
- ☐ Prefer not to say
- ☐ Not listed, please specify: ____________________________

Education What is the highest degree or level of school you have completed?

- ☐ Less than a high school diploma
- ☐ High school or equivalent (e.g. Scottish National level 5, GCSE)
- ☐ College/Sixth form (e.g. Scottish Highers/Higher National Certificate, Advanced Highers/Higher National Diploma, BTEC, NVQ, AS/A-levels)
- ☐ Further education college (e.g. HNC, HND)
- ☐ Vocational (e.g. apprenticeship, higher apprenticeship)
- ☐ Bachelor's degree (e.g. BA, BSc)
- ☐ Master's degree (e.g. MA, MSc, MEd)
- ☐ Doctorate or professional degree (e.g. MD, DDS, PhD)
- ☐ Prefer not to say
- ☐ Not listed, please specify: ____________________________

For peer review only

Postcode What is your postcode?

____

Meals Is your child eligible for free school meals?

- ☐ Yes
- ☐ No

Diagnosis Source Who diagnosed your child with ADHD?

- ☐ General Practitioner
- ☐ Pediatrician
- ☐ Psychiatrist
- ☐ Psychologist
- ☐ Neurologist
- ☐ Other, please specify: ______________________________

Diagnosis Age How old was your child when they were diagnosed with ADHD?

Diagnoses Other Does your child have any other mental health or neurodevelopmental diagnoses?

- ☐ Yes
- ☐ No

If yes, display this question:

Diagnoses List Which other diagnoses does your child have? Please select all that apply.

- ☐ Autism Spectrum Disorder
- ☐ Foetal Alcohol Syndrome
- ☐ Intellectual Disability
- ☐ Anxiety Disorder
- ☐ Depression
- ☐ Conduct Disorder
- ☐ Other, please specify: ______________________________

For peer review only

Medication Is your child currently taking any ADHD medication?

- ☐ Yes
- ☐ No

If yes, display this question:

Medication Details Which medicine is your child taking and what dosage?

- ☐ Medicine: ____________________________
- ☐ Dosage (e.g., XX mg, 2x a day): ____________________________

Medication Adherence Please select the response that is most true for your child.

	Always	Often	Sometimes	Rarely	Never
My child forgets to take their medication	○	○	○	○	○
My child changes the dosage of their medication	○	○	○	○	○
My child stops taking their medication for a while	○	○	○	○	○
My child decides to skip one of their medication dosages	○	○	○	○	○
My child uses their medication less than is prescribed	○	○	○	○	○

For peer review only

Strengths Please select the response that is most true for your child.

	Strongly Agree	Agree	Neither Disagree nor Agree	Disagree	Strongly Disagree
My child is able to focus really well on the things they are interested in	○	○	○	○	○
My child has lots of energy to get things done	○	○	○	○	○
My child has tons of ideas	○	○	○	○	○
My child is creative	○	○	○	○	○
My child has a warm personality	○	○	○	○	○
My child is full of enthusiasm	○	○	○	○	○
My child is good at coming up with solutions to problems	○	○	○	○	○
My child finds it easy to develop new interests (e.g., hobbies)	○	○	○	○	○

For peer review only

DBD Please select the option that best describes your child. Please give your answers on the basis of your child's behaviour over the last six months

	Not at All	Just a Little	Pretty Much	Very Much	Don't Know or Not Applicable
Often interrupts or intrudes on others (e.g., butts into conversations or games)	○	○	○	○	○
Has run away from home overnight at least twice while living in parental or parental surrogate home (or once without returning for a lengthy period)	○	○	○	○	○
Often argues with adults	○	○	○	○	○
Often lies to obtain goods or favors or to avoid obligations (i.e., "cons" others)	○	○	○	○	○
Often initiates physical fights with other members of his or her household	○	○	○	○	○
Has been physically cruel to people	○	○	○	○	○
Often talks excessively	○	○	○	○	○
Has stolen items of nontrivial value without confronting a victim (e.g., shoplifting, but without breaking and entering; forgery)	○	○	○	○	○
Is often easily distracted by extraneous stimuli	○	○	○	○	○

For peer review only

	Not at All	Just a Little	Pretty Much	Very Much	Don't Know or Not Applicable
Often engages in physically dangerous activities without considering possible consequences (not for the purpose of thrill-seeking), e.g., runs into street without looking	○	○	○	○	○
Often truant from school, beginning before age 13 years	○	○	○	○	○
Often fidgets with hands or feet or squirms in seat	○	○	○	○	○
Is often spiteful or vindictive	○	○	○	○	○
Often swears or uses obscene language	○	○	○	○	○
Often blames others for his or her mistakes or misbehavior	○	○	○	○	○
Has deliberately destroyed others' property (other than by fire setting)	○	○	○	○	○
Often actively defies or refuses to comply with adults' requests or rules	○	○	○	○	○
Often does not seem to listen when spoken to directly	○	○	○	○	○
Often blurts out answers before questions have been completed	○	○	○	○	○
Often initiates physical fights with others who do not live in his or her household (e.g., peers at school or in the neighborhood)	○	○	○	○	○
Often shifts from one uncompleted activity to another	○	○	○	○	○
Often has difficulty playing or engaging in leisure activities quietly	○	○	○	○	○

For peer review only

	Not at All	Just a Little	Pretty Much	Very Much	Don't Know or Not Applicable
Often fails to give close attention to details or makes careless mistakes in schoolwork, work, or other activities	○	○	○	○	○
Is often angry and resentful	○	○	○	○	○
Often leaves seat in classroom or in other situations in which remaining seated is expected	○	○	○	○	○
Is often touchy or easily annoyed by others	○	○	○	○	○
Often does not follow through on instructions and fails to finish schoolwork, chores, or duties in the workplace (not due to oppositional behavior or failure to understand instructions)	○	○	○	○	○
Often loses temper	○	○	○	○	○
Often has difficulty sustaining attention in tasks or play activities	○	○	○	○	○
Often has difficulty awaiting turn	○	○	○	○	○
Has forced someone into sexual activity	○	○	○	○	○
Often bullies, threatens, or intimidates others	○	○	○	○	○
Is often "on the go" or often acts as if "driven by a motor"	○	○	○	○	○
Often loses things necessary for tasks or activities (e.g., toys, school assignments, pencils, books, or tools)	○	○	○	○	○
Often runs about or climbs excessively in situations in which it is inappropriate (in adolescents or adults, may be	○	○	○	○	○

limited to subjective feelings of restlessness)					
Has been physically cruel to animals	○	○	○	○	○
Often avoids, dislikes, or is reluctant to engage in tasks that require sustained mental effort (such as schoolwork or homework)	○	○	○	○	○
Often stays out at night despite parental prohibitions, beginning before age 13 years	○	○	○	○	○
Often deliberately annoys people	○	○	○	○	○
Has stolen while confronting a victim (e.g., mugging, purse snatching, extortion, armed robbery)	○	○	○	○	○
Has deliberately engaged in fire setting with the intention of causing serious damage	○	○	○	○	○
Often has difficulty organizing tasks and activities	○	○	○	○	○
Has broken into someone else's house, building, or car	○	○	○	○	○
Is often forgetful in daily activities	○	○	○	○	○
Has used a weapon that can cause serious physical harm to others (e.g., a bat, brick, broken bottle, knife, gun)	○	○	○	○	○

For peer review only

RCADS-11 Please select the option that best describes your child. Please give your answers on the basis of your child's behaviour over the last six months.

	Never	Sometimes	Often	Always
My child has trouble going to school in the mornings because of feeling nervous or afraid	○	○	○	○
My child has no energy for things	○	○	○	○
My child worries when in bed at night	○	○	○	○
My child worries about what is going to happen	○	○	○	○
Nothing is much fun for my child anymore	○	○	○	○
All of a sudden my child will feel really scared for no reason at all	○	○	○	○
My child feels worthless	○	○	○	○
My child feels sad or empty	○	○	○	○
When my child has a problem, his/her heart beats really fast	○	○	○	○
My child is tired a lot	○	○	○	○
My child worries about looking foolish	○	○	○	○

For peer review only

SRQ We would like to learn more about your child's peer relationships. Please indicate how much each of the following statements applies to your child.

	Not True	Sort of True	Mostly True	Very True
My child has a "best friend" that he/she feels close to	○	○	○	○

If "Mostly True" or "Very True" selected display the following questions:

	Not True	Sort of True	Mostly True	Very True
This relationship is positive and beneficial for my child (affectionate, supportive, intimate)	○	○	○	○
This relationship is negative for my child (high conflict, aggression, betrayal, competitive)	○	○	○	○

Display following questions to all participants:

	Not True	Sort of True	Mostly True	Very True
My child has a group of friends at school that he/she hangs around with	○	○	○	○
My child has a group of friends at home that he/she likes to play with	○	○	○	○
My child has always had problems with peer relationships	○	○	○	○
My child seems to have fewer friends as he/she grows older	○	○	○	○
My child has difficulty making new friends	○	○	○	○
My child has difficulty keeping the friends that he/she makes	○	○	○	○

For peer review only

My child's friend(s) are similar to him/her in the way they act and the things they like

My child has problems with other children because he/she teases them

My child has problems with other children because he/she gets teased by them

My child has difficulty responding appropriately to teasing

For peer review only

S6: Parents end-of-study survey

DBD Please select the option that best describes your child. Please give your answers on the basis of your child's behaviour over the last six months

	Not at All	Just a Little	Pretty Much	Very Much	Don't Know or Not Applicable
Often interrupts or intrudes on others (e.g., butts into conversations or games)	○	○	○	○	○
Has run away from home overnight at least twice while living in parental or parental surrogate home (or once without returning for a lengthy period)	○	○	○	○	○
Often argues with adults	○	○	○	○	○
Often lies to obtain goods or favors or to avoid obligations (i.e., "cons" others)	○	○	○	○	○
Often initiates physical fights with other members of his or her household	○	○	○	○	○
Has been physically cruel to people	○	○	○	○	○
Often talks excessively	○	○	○	○	○
Has stolen items of nontrivial value without confronting a victim (e.g., shoplifting, but without breaking and entering; forgery)	○	○	○	○	○
Is often easily distracted by extraneous stimuli	○	○	○	○	○

For peer review only

	Not at All	Just a Little	Pretty Much	Very Much	Don't Know or Not Applicable
Often engages in physically dangerous activities without considering possible consequences (not for the purpose of thrill-seeking), e.g., runs into street without looking	◯	◯	◯	◯	◯
Often truant from school, beginning before age 13 years	◯	◯	◯	◯	◯
Often fidgets with hands or feet or squirms in seat	◯	◯	◯	◯	◯
Is often spiteful or vindictive	◯	◯	◯	◯	◯
Often swears or uses obscene language	◯	◯	◯	◯	◯
Often blames others for his or her mistakes or misbehavior	◯	◯	◯	◯	◯
Has deliberately destroyed others' property (other than by fire setting)	◯	◯	◯	◯	◯
Often actively defies or refuses to comply with adults' requests or rules	◯	◯	◯	◯	◯
Often does not seem to listen when spoken to directly	◯	◯	◯	◯	◯
Often blurts out answers before questions have been completed	◯	◯	◯	◯	◯
Often initiates physical fights with others who do not live in his or her household (e.g., peers at school or in the neighborhood)	◯	◯	◯	◯	◯
Often shifts from one uncompleted activity to another	◯	◯	◯	◯	◯
Often has difficulty playing or engaging in leisure activities quietly	◯	◯	◯	◯	◯

For peer review only

	Not at All	Just a Little	Pretty Much	Very Much	Don't Know or Not Applicable
Often fails to give close attention to details or makes careless mistakes in schoolwork, work, or other activities	○	○	○	○	○
Is often angry and resentful	○	○	○	○	○
Often leaves seat in classroom or in other situations in which remaining seated is expected	○	○	○	○	○
Is often touchy or easily annoyed by others	○	○	○	○	○
Often does not follow through on instructions and fails to finish schoolwork, chores, or duties in the workplace (not due to oppositional behavior or failure to understand instructions)	○	○	○	○	○
Often loses temper	○	○	○	○	○
Often has difficulty sustaining attention in tasks or play activities	○	○	○	○	○
Often has difficulty awaiting turn	○	○	○	○	○
Has forced someone into sexual activity	○	○	○	○	○
Often bullies, threatens, or intimidates others	○	○	○	○	○
Is often "on the go" or often acts as if "driven by a motor"	○	○	○	○	○
Often loses things necessary for tasks or activities (e.g., toys, school assignments, pencils, books, or tools)	○	○	○	○	○
Often runs about or climbs excessively in situations in which it is inappropriate (in adolescents or adults, may be	○	○	○	○	○

For peer review only

limited to subjective feelings of restlessness)					
Has been physically cruel to animals	○	○	○	○	○
Often avoids, dislikes, or is reluctant to engage in tasks that require sustained mental effort (such as schoolwork or homework)	○	○	○	○	○
Often stays out at night despite parental prohibitions, beginning before age 13 years	○	○	○	○	○
Often deliberately annoys people	○	○	○	○	○
Has stolen while confronting a victim (e.g., mugging, purse snatching, extortion, armed robbery)	○	○	○	○	○
Has deliberately engaged in fire setting with the intention of causing serious damage	○	○	○	○	○
Often has difficulty organizing tasks and activities	○	○	○	○	○
Has broken into someone else's house, building, or car	○	○	○	○	○
Is often forgetful in daily activities	○	○	○	○	○
Has used a weapon that can cause serious physical harm to others (e.g., a bat, brick, broken bottle, knife, gun)	○	○	○	○	○

For peer review only

RCADS-11 Please select the option that best describes your child. Please give your answers on the basis of your child's behaviour over the last six months.

	Never	Sometimes	Often	Always
My child has trouble going to school in the mornings because of feeling nervous or afraid	○	○	○	○
My child has no energy for things	○	○	○	○
My child worries when in bed at night	○	○	○	○
My child worries about what is going to happen	○	○	○	○
Nothing is much fun for my child anymore	○	○	○	○
All of a sudden my child will feel really scared for no reason at all	○	○	○	○
My child feels worthless	○	○	○	○
My child feels sad or empty	○	○	○	○
When my child has a problem, his/her heart beats really fast	○	○	○	○
My child is tired a lot	○	○	○	○
My child worries about looking foolish	○	○	○	○

For peer review only

EMA feedback We would appreciate getting your feedback on the smart-phone based surveys that your child took part in.

	Almost never	Sometimes	About half the time	Most of the time	All of the time
My child found doing the smartphone surveys fun	○	○	○	○	○
I would allow my child to participate in a study like this involving smartphone surveys again	○	○	○	○	○

For peer review only

Suggestions Do you have thoughts or suggestions on how the smartphone surveys could be improved?

____

Illuminating the daily life experiences of adolescents with and without ADHD: Protocol for an ecological momentary assessment study

Journal:	*BMJ Open*
Manuscript ID	bmjopen-2023-077222.R1
Article Type:	Protocol
Date Submitted by the Author:	18-Aug-2023
Complete List of Authors:	Murray, Aja; University of Edinburgh, Department of Psychology<br>Speyer, Lydia; University of Edinburgh<br>Thye, Melissa; University of Edinburgh, Department of Psychology<br>Stewart, Tracy; University of Edinburgh<br>Obsuth, Ingrid; University of Edinburgh, Clinical and Health Psychology Department<br>Kane, Jennifer; University of Edinburgh, Clinical and Health Psychology Department<br>Whyte, Katie; St Andrew’s University, Department of Psychology<br>Devaney, John; University of Edinburgh<br>Rohde, Luis; Universidade Federal do Rio Grande do Sul<br>Ushakova, Anastasia; Lancaster University<br>Rhodes, Sinead ; University of Edinburgh
**Primary Subject Heading**:	Mental health
Secondary Subject Heading:	Neurology
Keywords:	Child & adolescent psychiatry < PSYCHIATRY, Depression & mood disorders < PSYCHIATRY, Anxiety disorders < PSYCHIATRY, Adolescent

Illuminating the daily life experiences of adolescents with and without ADHD: Protocol for an ecological momentary assessment study

Aja Louise Murray[1], Lydia Speyer[1,2], Melissa Thye[1], Tracy M. Stewart[3], Ingrid Obsuth[4], Jennifer Kane[4], Katie Whyte[5], John Devaney[6], Luis Rohde[7], Anastasia Ushakova[8], Sinead Rhodes[9]

[1]Department of Psychology, University of Edinburgh, UK

[2]Department of Psychology, University of Cambridge, UK

[3]Moray School House of Education and Sport, University of Edinburgh, UK

[4]Clinical and Health Psychology Department, University of Edinburgh, UK

[5]Department of Psychology, St Andrew's University, UK

[6] School of Social and Political Science, University of Edinburgh, UK

[7] ADHD Outpatient Program and Developmental Psychiatry Program, Hospital de Clinicas de Porto Alegre, Federal University of Rio Grande do Sul, Porto Alegre, Brazil, UniEduk, Brazil and National Institute of Developmental Psychiatry & National Center for Research and Innovation in Mental Health, Sao Paulo, Brazil

[8] Centre for Computing, Health Informatics and Statistics, Lancaster University, United Kingdom

[9]Centre for Clinical Brain Sciences, University of Edinburgh, UK

*Corresponding author at: 7 George Square, Edinburgh, EH8 9JZ, email: aja.murray@ed.ac.uk

For peer review only

Abstract

Introduction

Adolescents with Attention-Deficit/Hyperactivity Disorder (ADHD) are at elevated risk of a range of difficulties, among which emotion regulation, peer, and co-occurring mental health problems are prominent challenges. To better support adolescents with ADHD, ecologically valid interventions that can be embedded in daily life to target the most proximal antecedents of these challenges are needed. Ecological momentary assessment (EMA) designs are ideally suited to meeting this need.

Methods and analyses

In the mental health in the moment ADHD (MHIM-ADHD) study, we will use an EMA design to capture the daily life experiences of approximately 120 adolescents aged 11-14 years with a clinical diagnosis of ADHD and the same number of age- and gender-matched peers without a diagnosis of ADHD. We will combine this with comprehensive information gathered from online surveys. Analysing the data using techniques such as dynamic structural equation modelling (DSEM), we will examine, among other research questions, the role of emotion regulation and peer problems in mediating the links between characteristics of ADHD and commonly co-occurring outcomes such as anxiety, depression, and conduct problems. The results can help inform interventions to support improved peer functioning and emotion regulation for adolescents with ADHD.

Ethics and dissemination

This study received a favourable ethical opinion through the National Health Service (NHS) ethical review board and the University of Edinburgh PPLS Research Ethics panel. Results will be disseminated through journal publications, conferences and seminar presentations and to relevant stakeholders, such as those with ADHD, their families, and clinicians.

Keywords

Attention Deficit Hyperactivity Disorder/ADHD, Ecological Momentary Assessment, Emotion Regulation, Peer Problems

Strengths and limitations of this study

- This study will use an ecological momentary assessment design to collect high temporal resolution, ecologically value daily life experience data from young people with ADHD.
- It will use cutting edge statistical analysis techniques to disentangle different aspects of emotional functioning in daily life to identify key intervention targets.
- A limitation is that data will be collected only over a short period, therefore, developmental changes will not be captured.
- Further, EMA methods may be vulnerable to low response rates, careless responding, or drop-out and the reliance on self-reports can be associated with reporting biases.

For peer review only

Introduction

ADHD symptoms may increase the risk of experiencing a range of difficulties in adolescence (1–3) and prominent among these are inter-related challenges in emotion regulation and peer relationships (4). These have been suggested to contribute to the development of co-occurring mental health conditions such as anxiety, depression, and conduct problems (5–7); outcomes identified as a high priority for further research in consultations with neurodivergent expert-by-experience consultations (Embracing Complexity, 2022). However, critical reviews (8–10) have suggested a strong need for better interventions to support neurodivergent adolescents facing these challenges. In particular, current interventions have been criticised for a lack of generalisability to real-life contexts, for not directly targeting the interconnections between emotion dysregulation and peer problems, and for a lack of tailoring to the specific needs of adolescents (e.g., Morris et al., 2021). The goal of the present study is to provide evidence that helps address this gap, using a data collection technique that gathers rich 'in-the-moment' data about the daily life experiences and functioning of adolescents with and without ADHD.

Difficulties with emotion regulation among children and adolescents with ADHD are well documented (4,11). Emotion regulation can be defined as the set of processes by which individuals evaluate, inhibit, or modify their emotional reactions in order to show a socially appropriate behaviour and/or achieve some goal (12). Emotional dysregulation may manifest in a number of ways, including in impaired emotion recognition or understanding, a lack of empathy/callous-unemotional traits and emotional lability or reactivity. In comparison with neurotypical peers, youth with ADHD show greater emotional lability or reactivity (13) characterized by more intense, under-controlled, or rapidly shifting emotions than are appropriate for developmental stage and context and which consequently impacts functioning (14). This may include dysregulation of positive emotions leading to excessive excitability (1).

Emotion dysregulation often exists alongside and has been proposed to contribute to peer difficulties such as bullying victimisation and perpetration, peer aggression, rejection, dislike, and conflict among adolescents with ADHD (1,15). As a result of emotion regulation difficulties, adolescents with ADHD may react more strongly and more aggressively to provocations, making them targets for victimisation and placing them at higher risk of showing reactive aggression (16). They may become over-excited and experience resultant difficulties in attending to what is required of them in a given social situation (1). A role for rejection sensitivity, defined as the tendency to anxiously anticipate, over-perceive, and react intensely to social rejection (potentially arising as a result of repeated experiences of rejection), has also been proposed (17,18). Intense and dysregulated reactions to perceived rejection (e.g., excessive reassurance seeking or anger) may be experienced as aversive to peers and result in a 'self-fulfilling prophecy' of peer rejection (18). Overall, it has been noted that some youth with ADHD, as a result of emotion regulation difficulties, may be perceived as intense, disruptive, or overly exuberant, potentially leading to greater exposure to dislike, rejection, and victimisation (15). Indeed, given their inter-connectedness, the self-regulation of emotions, alongside behavioural inhibition, is thought to be one of the most important aspects of interventions to support better social functioning in adolescence (9).

Emotion dysregulation and peer problems are also thought to contribute to the development of co-occurring mental health and behavioural challenges associated with ADHD symptoms. For example, previous research in children and adolescents has found that peer issues and emotion regulation mediate the associations between ADHD symptoms and internalising problems such as anxiety and depression (5,6,19,20) and externalising problems such as aggression, oppositional, and conduct problem behaviours (21,22).

However, the daily life mechanisms underpinning these links and - correspondingly - the most proximal targets for intervention remain poorly understood, particularly in the critical period of adolescence. Ecological momentary assessment (EMA) has proven valuable in

For peer review only

child and adolescent mental health research for capturing symptoms and related experiences in near real time and in the flow of daily life and can thus inform more ecologically embedded intervention approaches (23). It is particularly valuable for capturing concepts such as emotional (dys)regulation and social interactions in daily life contexts (6). For example, EMA data can be used to provide measures of different aspects of emotional functioning in daily life, such as overall levels of negative affect, affective lability, emotional inertia, and emotional reactivity to stressors or provocations (24,25).

There have, however, been only a handful EMA studies of young people with ADHD during the critical period of adolescence (26,27). Reviewing the literature on EMA studies of ADHD populations, a recent review (26) identified less than 10 studies conducted in adolescents, most of which had small sample sizes and none of which addressed the links between emotion regulation, peer issues and co-occurring mental health and behavioural outcomes. Only one previous EMA study has examined the impact of emotion regulation on peer issues and mental health outcomes in a sample of adolescents with ADHD (1). Using a daily diary design they found that adolescents with ADHD tend to experience more negative emotion, less positive emotion, and more emotional variability. Variability in some emotions was associated with outcomes such as social functioning and externalising problems.

These initial findings point to the value of further exploring, using EMA, the daily life manifestation and impacts of emotion regulation and peer interactions in adolescents with ADHD. For example, as well as seeking to extend these findings to examine a wider range of emotion regulation and peer issues, it will be valuable to examine factors that may impact these domains, their inter-connections, and their links to mental health and behavioural outcomes This will provide illumination on further modifiable targets for intervention, such as physical activity, improved sleep, secure attachment to caregivers, and positive teacher-student relationships (25,28–30). Further, strengths associated with ADHD characteristics, such as a high level of energy and drive, the ability to focus intensely in tasks of interest, and positive temperamental factors (31) will be explored as these too could potentially be leveraged as part of more effective interventions.

We will thus use an EMA design to illuminate how, in the flow of their daily lives, adolescents with ADHD may experience difficulties with emotion regulation and peer problems, how these may contribute to co-occurring mental health and behavioural problems. We will also examine other potential modifiable targets for intervention that may impact emotion regulation, peer problems, mental health and behaviour as well as their links among adolescents with ADHD (e.g., ADHD strengths, relationships, sleep, physical activity). Core research questions are:

- Which aspects of emotion regulation differ between adolescents with and without ADHD, e.g., negative or positive emotional lability, emotional reactivity, emotional inertia, emotion regulation strategy use and emotion regulation strategy success?
- Which aspects of emotion regulation mediate the association between ADHD diagnosis/symptoms and peer problems in daily life?
- Do emotion regulation and peer issues mediate the links between ADHD diagnosis/symptoms and anxiety, depression and conduct problems?

Additional exploratory analyses may be used to answer a range of additional questions such as exploration of moderation of the links between ADHD diagnosis/symptoms and outcomes to identify other potential intervention targets.

Methods and analyses

For peer review only

Participants

Participants are expected to include approximately 120 adolescents aged 11-14 years with a clinical diagnosis of ADHD and 120 matched controls. The clinical sample will be recruited via Child and Adolescent Mental Health Services (CAMHS) in two primary sites in the UK (one in Scotland and one in England) and community partners. In CAMHS, the recruitment procedure will vary from service to service; however, in general participants will be recruited at medication clinics and through ongoing casework.

A matched control (on age +/- 3 months, gender, and if possible, city and socioeconomic status based on Scottish Index of Multiple Deprivation in Scotland and Index of Multiple Deprivation in England) will be selected for each participant with a clinical diagnosis of ADHD from a larger pool of participants taking part in adolescent mental health studies (co-)led by the first author. To maximise relevance to community-ascertained samples, minor modifications to the protocol will be made, detailed in the Measures section.

Inclusion and exclusion criteria

Inclusion criteria for the clinical sample will be: aged 11-14, a formal clinical diagnosis of ADHD (parent-reported in the case of the youth recruited via the community), the ability to provide informed assent and parental informed consent to participate, and the ability to independently complete online surveys and EMA protocols. There will be no explicit exclusion criteria based on co-occurring neurodevelopmental conditions as ADHD commonly co-occurs with other neurodevelopmental conditions (32) and excluding youth with these conditions would undermine the representativeness of the sample. It would also preclude the opportunity to illuminate how ADHD characteristics may interact with those of other neurodevelopmental conditions (33). For similar reasons and given our goal of illuminating candidate mechanisms by which ADHD characteristics may impact mental health and behavioural outcomes, we will also not impose any exclusion criteria related to any other co-occurring psychiatric issues (e.g., anxiety, depression, conduct disorder). However, we acknowledge that the requirement to be able to complete the online surveys and EMA surveys independently may result in the exclusion of some young people (e.g., those with a moderate to severe intellectual disability). Adaptation of the protocol to better include these groups will be the subject of future research. Inclusion and exclusion criteria for the control sample will be identical, except that a formal clinical diagnosis of ADHD will be an exclusion criterion for controls.

Patient and public involvement

Adolescents with ADHD and parents of adolescents with ADHD are the most important stakeholders in this project. Their views have informed the project design, including the current protocol and will continue to inform the project and the dissemination of its outputs. Young person and parent advisors were recruited via the ADHD charities and organisations with whom our team has collaborative links. Examples of major changes made to the protocol based on their input included extending the inclusion criteria to include lower ages, reducing the length of the surveys, including more positively- and fewer negatively-worded items, removing elements of the project that would have involved location tracking and wearable technology, adding a measure of rejection sensitivity, and embedding more fun and educational elements into the study, such as providing ‘ADHD facts’ at the end of each completed EMA survey.

Data collection

For peer review only

Data collection is projected to begin in August 2023 and be completed in 12 months. Figure 1 summarises the study flow. Informed parental consent and adolescent assent will be collected, then both adolescents and their parents will be invited to complete an online ‘intake’ survey. This reflects the importance of obtaining a ‘multi-informant’ perspective (34–36).

On completion of the online intake survey, adolescent participants will initiate the *EMA surveys*. They will be provided with both written and video instructions on how to take part, and offered on-boarding support via a video call with study staff. Participants will use their own smartphones where possible; however, to avoid selection biases and promote inclusion, study smartphones will be available. One week into the EMA period, they will be offered the option of scheduling a ‘check-in’. An email helpline will also be available for participants to receive help on any issues they encounter.

EMA data collection will take place over a two-week period and involve 5 measures per day, all administered via a smartphone application. Four of these measures will be delivered at quasi-random intervals (random within four blocks) and include identical measures of momentary experiences. The 5th will be a brief ‘daily diary’ that captures concepts that vary over slightly longer timescales (e.g., sleep, medication use, physical activity). All measures will be designed to take less than 2 minutes as long questionnaires are associated with increased burden and lower compliance and our YPAG/PAG emphasised the importance of minimising burden (37).

At the end of the two-week EMA period, participants will complete an online *end-of study* survey. Here they will complete repeat measures of a subset of the questions administered in the original survey, as well as a brief set of questions capturing their participation experiences. Full questionnaires are provided in Supplementary Materials.

Measures

Adolescent measures

The measures that adolescents will complete over the course of the study are presented in **Table 1**. Full descriptions of the measures are provided in Supplemental Materials.

Parent measures

The measures that parents will complete over the course of the study are presented in **Table 2**. Full descriptions of the measures are provided in Supplemental Materials.

For peer review only

Table 1

Measures for adolescent surveys

Phase	Measures	Sources	No items
Intake			
	Demographics	Gender, Date of Birth, Ethnicity, Postcode	4
	Internalising problems	Revised Child Anxiety and Depression Scale (RCADS-11) (38)	11
	ADHD Strengths*	Newly developed measure	8
	Emotion dysregulation	Difficulties in Emotion Regulation Scale (DERS-16) (39)	16
	Emotion regulation strategy use	Emotion Regulation Questionnaire adapted for Children and Adolescents (ERQ-CA) (40)	10
	Peer problems and friendships	Social Relationships Questionnaire (SRQ) (41,42)	11
	Attachment security	Inventory of Parent and Peer Attachment (IPPA-10) (43)	10
	Quality of teacher-student relationships	Measure used in prior longitudinal study (44)	3
	Self-esteem	Rosenberg Self-Esteem questionnaire (45)	10
	Autistic traits	Autism Symptom SElf-ReporT (ASSERT) (46,47)	7
	Rejection sensitivity	Child Rejection Sensitivity Questionnaire (CRSQ) (48,49)	6
EMA			
	ADHD symptoms	Adapted from DSM criteria and prior ADHD EMA studies (3)	3
	Medication use	Has medication been taken since last assessment	1
	Emotions	Adapted from Positive Affect Negative Affect Schedule expanded (PANAS-X) (50)	6
	Emotion regulation strategies	Adapted version of the Emotion Regulation Questionnaire (51)	2
	Peer interactions	Newly developed measure	4
Daily Diary			
	Medication use	Whether and when medication was taken in last 24 hours	2
	Physical activity	Adapted from an open EMA item pool (52)	3
	Sleep	Abbreviated version of the Consensus Sleep Diary core items (53)	7
End			
	Internalising problems	Revised Child Anxiety and Depression Scale (RCADS-11) (38)	11
	Participation experiences	EMA and daily diary enjoyment, burden, and impact on routine; suggestions	4

Note. EMA, ecological momentary assessment; No, number. *Not completed by the control sample.

For peer review only

Table 2

Measures for parent surveys

Phase	Measures	Sources	No items
Intake			
	Demographics	Respondent Gender, Ethnicity, Education, Postcode, Free School Meals	5
	Child's ADHD or other diagnoses	Diagnosis Source, Diagnosis Age, Other Diagnoses	3
	Child's ADHD medication use	Medication Adherence Report Scale (54)	5
	Child's ADHD strengths*	Newly developed measure	8
	Child's ADHD, conduct and oppositional symptoms	Disruptive Behavior Disorder Rating Scale (DBD) (55)	45
	Child's internalising problems	Revised Child Anxiety and Depression Scale (RCADS-11) (38)	11
	Child's peer conflict and friendships	Social Relationships Questionnaire (SRQ) (41,42)	11
End			
	Child's ADHD, conduct and oppositional symptoms	Disruptive Behavior Disorder Rating Scale (DBD) (55)	45
	Child's internalising problems	Revised Child Anxiety and Depression Scale (RCADS-11) (38)	11
	Child's participation experiences	Study enjoyment and likelihood to participate again; suggestions	3

Note. No, number. *Not completed by parents of the control sample.

For peer review only

Piloting

Prior to the main data collection, the protocol will be piloted in a sample of n=10 adolescents (5 with and 5 without ADHD). Further modifications may be made to the protocol after piloting.

Statistical Procedure

Data will be analysed using techniques such as dynamic structural equation modelling (DSEM) (56–58). For example, using two-level DSEM with participant random effects, it will be possible to derive individual-level indices of the mean levels, variability, and covariation of different concepts measured in the EMA. For example, we can derive indices of emotion regulation such as: variability in emotional state (lability), strength of relations between prior and subsequent emotional state (inertia), strength of relations between an event (e.g., a peer conflict) and emotional state (reactivity) and examine their links to peer, mental health, and behavioural issues, including as mediators of the links between ADHD symptoms and these outcomes. They can be studied alongside emotion regulation strategy use, resulting emotional states, and differences in these between adolescents with and without ADHD. Specific confirmatory analyses will be pre-registered and suitable multiple comparisons correction approaches selected as relevant.

Sample size justification

Our sample size (n=120 adolescents with ADHD and n=120 without) draws on several sample size determination approaches. It is partly based on a resource constraint approach, i.e., we will maximise the sample size attainable within the resources available (59). It is informed by Monte Carlo simulation studies using DSEM population models with varying effect sizes for the within- and between-person structures. These have suggested that a minimum of 50 respondents with 10 measurement points are needed to avoid small sample bias for random effects and to provide adequate coverage for effect sizes at the within- and between-person level that are likely to be of clinical importance (60,61). This is supplemented by our own simulations using population parameters derived from data gathered using similar designs in previous studies suggest that 100 respondents (50 per group) measured 50 times provides adequate coverage for realistic parameter estimates e.g.: https://osf.io/pvrma/. Taken together, our choice of n=120 per group balances feasibility with statistical precision and includes buffer for missing data and attrition.

Ethics and dissemination

Ethical review for the collection of the data from the clinically ascertained sample will follow a three-stage process in which sponsorship will be sought from ACCORD, followed by ethical review by the National Health Service (NHS) ethical review board, and finally, research and development approval. Ethical review for the collection of data based on community and school recruitment will be by the University of Edinburgh PPLS Research Ethics panel. Results will be disseminated through journal publications, conferences and seminar presentations and to relevant stakeholders, such as those with ADHD, their families, and clinicians.

For peer review only

Discussion

The current protocol describes the planned design for a combined traditional and EMA survey study (62) of the daily life manifestation and impacts of emotion regulation and peer issues among adolescents with and without ADHD. Building on previous evidence that emotion regulation and peer problems are common and inter-connected among youth with ADHD (1,15), we will seek to illuminate the most proximal targets in the daily lives of these youth to leverage in interventions. Adolescence is a critical transition period for emotion regulation and social development that may be particularly challenging for youth with ADHD (15). Reflecting the importance of involving those most affected by the research in mental health and neurodevelopmental research (63), the project has been and will continue to be informed by two panels of experts by experience, namely adolescents with ADHD and parents of adolescents with ADHD.

The study is motivated by a need for new data to inform more ecologically valid interventions that can provide effective improvements to adolescents' emotional and social functioning in their daily lives (9). Though new, more integrative intervention approaches continue to be developed (64), the lack of attempts to address emotion regulation and social skills together and a lack of generalizability to real life functioning are two major critiques of existing supports for adolescents with ADHD (9). By gathering information about how emotion regulation and (potential) peer problems manifest, how they are linked and how they contribute to the development of co-occurring mental health or behavioural concerns, our findings can inform improvements to interventions to enhance their ecological validity.

Our findings may be particularly valuable for informing smartphone-based interventions (65). These show strong promise for adolescents with ADHD because smartphone use is highly embedded within the daily routines of young people, can allow the regular practice of skills within ecological context, and can be responsive to incoming data that, for example, may indicate high moments of risk for adverse outcomes (in 'ecological momentary interventions').Their scale-able and easily accessible format can also help make interventions more widely and more quickly available to the population of youth who may benefit from them. However, recent reviews of smartphone applications for ADHD have suggested that very few available applications are based on a robust evidence base (65). Further, mental health smartphone-based applications have been shown to be associated with low compliance (66) and are frequently abandoned after only a small number of uses (67). Our study can help to gain insights into the factors that can promote better engagement with smartphone-delivered interventions. This will be particularly important for adolescents with ADHD, who may find sustained engagement with interventions difficult, necessitating tailored protocols that, for example, provide immediate and high value rewards for compliance.

Our findings can also help accelerate progress in the application of EMA methodologies in populations of adolescents with ADHD. To date, despite their promise, very few such studies have been conducted (26,27). By providing a template for such research (which we will make openly available in the form of openly accessible study materials, including measures and analysis code), and documenting challenges, solutions, and benefits, this study can facilitate wider application of this technique.

Limitations

Though a large body of research has investigated optimal design features for EMA in general (37,68–70), the relative novelty of the approach in adolescents with ADHD (see, e.g., (1,71) for notable exceptions means that there is little past research to guide the selection of measures, sampling frequency and schedule, incentives, strategies to promote engagement, and other design features. More broadly, there remains a lack of attention paid to the development and validation of measures for EMA (72) and this means that there is

little psychometric evidence underpinning available EMA measures of core constructs for the study such as emotion regulation and peer problems (73). Our study will thus help contribute to the development and validation of robust EMA measures but will not benefit from a strong pre-existing evidence base to guide which measures are likely to show the best psychometric properties. Finally, though EMA can overcome some of the reporting difficulties associated with traditional surveys (e.g., reporting biases and accuracies due to their reliance on retrospective recall), they are not invulnerable to self-report biases. For example, previous research has suggested that some adolescents with ADHD may be vulnerable to positive illusory biases that may result in a lack of awareness of difficulty and thus overly optimistic reporting of functioning (74).

For peer review only

Acknowledgements

We are grateful to Chloe Bolota, Katie Dryburgh, Yi Yang, Zhuoni Xiao, Xinxin Zhu, and Ercan Ozdemir for their help in identifying and piloting measures used in this study.

Funding

The research described within this report was supported by a Medical Research Foundation (MRF) grant (Reference: MRF-001-0014-RG-MURR-C0932) and ALM was additionally supported by a Wellcome Trust Career Development Grant (REF: 225364/Z/22/Z).

Competing Interests

Luis Augusto Rohde has received grant or research support from, served as a consultant to, and served on the speakers' bureau of Abdiibrahim, Abbott, Aché, Bial, Medice, Novartis/Sandoz, Pfizer/Upjohn, and Shire/Takeda in the last three years. The ADHD and Juvenile Bipolar Disorder Outpatient Programs chaired by Dr Rohde have received unrestricted educational and research support from the following pharmaceutical companies in the last three years: Novartis/Sandoz and Shire/Takeda. Dr Rohde has received authorship royalties from Oxford Press and ArtMed.

Author Contributions

Conceptualisation: ALM; Funding acquisition: ALM AU SR TS LR IO JD LR ; Methodology: ALM JK LS IO MT; Writing – Original Draft: ALM; Writing – Review & Editing: ALM, LS, MT, TS, IO, JK, KW, JD, LR, AU, SR

References

1. Breaux R, Langberg JM, Swanson CS, Eadeh HM, Becker SP. Variability in positive and negative affect among adolescents with and without ADHD: Differential associations with functional outcomes. Journal of affective disorders. 2020;274:500–7.

2. Murray AL, Booth T, Eisner M, Auyeung B, Murray G, Ribeaud D. Sex differences in ADHD trajectories across childhood and adolescence. Developmental science. 2018;e12721.

3. Pedersen SL, Kennedy TM, Joseph HM, Riston SJ, Kipp HL, Molina BS. Real-world changes in adolescents' ADHD symptoms within the day and across school and non-school days. Journal of abnormal child psychology. 2020;48(12):1543–53.

4. Faraone SV, Rostain AL, Blader J, Busch B, Childress AC, Connor DF, et al. Practitioner Review: Emotional dysregulation in attention-deficit/hyperactivity disorder–implications for clinical recognition and intervention. Journal of Child Psychology and Psychiatry. 2019;60(2):133–50.

5. Antony EMA, Pihlajamäki MR, Speyer LG, Murray AL. Does emotion dysregulation mediate the association between ADHD symptoms and internalizing problems? A longitudinal within-person analysis in a large population-representative study. 2021;

6. Murray AL, Wong SC, Obsuth I, Rhodes S, Eisner M, Ribeaud D. An ecological momentary assessment study of the role of emotional dysregulation in co-occurring ADHD and internalising symptoms in adulthood. Journal of affective disorders. 2021;281:708–13.

For peer review only

7. Steinberg EA, Drabick DA. A developmental psychopathology perspective on ADHD and comorbid conditions: the role of emotion regulation. Child Psychiatry & Human Development. 2015;46(6):951–66.

8. Lenzi F, Cortese S, Harris J, Masi G. Pharmacotherapy of emotional dysregulation in adults with ADHD: A systematic review and meta-analysis. Neuroscience and Biobehavioral Reviews. 2018;359–67.

9. Morris S, Sheen J, Ling M, Foley D, Sciberras E. Interventions for adolescents with ADHD to improve peer social functioning: A systematic review and meta-analysis. Journal of attention disorders. 2021;25(10):1479–96.

10. Vacher C, Goujon A, Romo L, Purper-Ouakil D. Efficacy of psychosocial interventions for children with ADHD and emotion dysregulation: a systematic review. Psychiatry research. 2020;113151.

11. Shaw P, Stringaris A, Nigg J, Leibenluft E. Emotion dysregulation in attention deficit hyperactivity disorder. Focus. 2015;14(1):127–44.

12. Bunford N, Evans SW, Wymbs F. ADHD and emotion dysregulation among children and adolescents. Clinical child and family psychology review. 2015;18(3):185–217.

13. Graziano PA, Garcia A. Attention-deficit hyperactivity disorder and children's emotion dysregulation: A meta-analysis. Clinical psychology review. 2016;46:106–23.

14. Shaw P, Stringaris A, Nigg J, Leibenluft E. Emotion dysregulation in attention deficit hyperactivity disorder. American Journal of Psychiatry. 2014;171(3):276–93.

15. Bunford N, Evans SW, Langberg JM. Emotion dysregulation is associated with social impairment among young adolescents with ADHD. Journal of Attention Disorders. 2018;22(1):66–82.

16. Murray AL, Zych I, Ribeaud D, Eisner M. Developmental relations between ADHD symptoms and bullying perpetration and victimisation in adolescence. 2020;

17. Babinski DE, Kujawa A, Kessel EM, Arfer KB, Klein DN. Sensitivity to peer feedback in young adolescents with symptoms of ADHD: examination of neurophysiological and self-report measures. Journal of abnormal child psychology. 2019;47:605–17.

18. Lesnick J, Mendle J. Rejection sensitivity and negative urgency: A proposed framework of intersecting risk for peer stress. Developmental Review. 2021;62:100998.

19. Humphreys KL, Katz SJ, Lee SS, Hammen C, Brennan PA, Najman JM. The association of ADHD and depression: Mediation by peer problems and parent–child difficulties in two complementary samples. Journal of abnormal psychology. 2013;122(3):854.

20. Roy A, Hartman CA, Veenstra R, Oldehinkel AJ. Peer dislike and victimisation in pathways from ADHD symptoms to depression. European child & adolescent psychiatry. 2015;24(8):887–95.

21. Yip VT, Ang RP, Ooi YP, Fung DS, Mehrotra K, Sung M, et al. The association between attention problems and internalizing and externalizing problems: The mediating role of peer problems. In: Child & Youth Care Forum. Springer; 2013. p. 571–84.

22. Slaughter KE, Leaberry KD, Fogleman ND, Rosen PJ. Reactive and proactive aggression in children with and without ADHD and negative emotional lability. Social Development. 2019;

For peer review only

23. Russell MA, Gajos JM. Annual Research Review: Ecological momentary assessment studies in child psychology and psychiatry. Journal of Child Psychology and Psychiatry. 2020;61(3):376–94.

24. Speyer LG, Brown RH, Ribeaud D, Eisner M, Murray AL. The role of moment-to-moment dynamics of perceived stress and negative affect in co-occurring ADHD and internalising symptoms. Journal of Autism and Developmental Disorders. 2022;1–11.

25. Talty A, Speyer LG, Eisner M, Ribeaud D, Murray AL, Obsuth I. The role of student-teacher relationships in the association between adverse parenting and emotion dynamics–combining longitudinal and ecological momentary assessment data. 2022;

26. Koch ED, Moukhtarian TR, Skirrow C, Bozhilova N, Asherson P, Ebner-Priemer UW. Using e-diaries to investigate ADHD–State-of-the-art and the promising feature of just-in-time-adaptive interventions. Neuroscience & Biobehavioral Reviews. 2021;127:884–98.

27. Miguelez-Fernandez C, de Leon SJ, Baltasar-Tello I, Peñuelas-Calvo I, Barrigon ML, Capdevila AS, et al. Evaluating attention-deficit/hyperactivity disorder using ecological momentary assessment: a systematic review. ADHD Attention Deficit and Hyperactivity Disorders. 2018;10(4):247–65.

28. Lunsford-Avery JR, Krystal AD, Kollins SH. Sleep disturbances in adolescents with ADHD: A systematic review and framework for future research. Clinical psychology review. 2016;50:159–74.

29. Neudecker C, Mewes N, Reimers AK, Woll A. Exercise interventions in children and adolescents with ADHD: a systematic review. Journal of attention disorders. 2019;23(4):307–24.

30. Wylock JF, Borghini A, Slama H, Delvenne V. Child attachment and ADHD: a systematic review. European child & adolescent psychiatry. 2021;1–12.

31. Mahdi S, Viljoen M, Massuti R, Selb M, Almodayfer O, Karande S, et al. An international qualitative study of ability and disability in ADHD using the WHO-ICF framework. European child & adolescent psychiatry. 2017;26(10):1219–31.

32. Rong Y, Yang CJ, Jin Y, Wang Y. Prevalence of attention-deficit/hyperactivity disorder in individuals with autism spectrum disorder: a meta-analysis. Research in Autism Spectrum Disorders. 2021;83:101759.

33. Rosello R, Martinez-Raga J, Mira A, Pastor JC, Solmi M, Cortese S. Cognitive, social, and behavioral manifestations of the co-occurrence of autism spectrum disorder and attention-deficit/hyperactivity disorder: A systematic review. Autism. 2022;26(4):743–60.

34. De Los Reyes A. Strategic objectives for improving understanding of informant discrepancies in developmental psychopathology research. Development and psychopathology. 2013;25(3):669–82.

35. Murray AL, Booth T, Ribeaud D, Eisner M. Disagreeing about development: An analysis of parent-teacher agreement in ADHD symptom trajectories across the elementary school years. International journal of methods in psychiatric research. 2018;e1723.

36. Murray AL, Ribeaud D, Eisner M, Murray G, McKenzie K. Should We Subtype ADHD According to the Context in Which Symptoms Occur? Criterion Validity of Recognising Context-Based ADHD Presentations. Child Psychiatry & Human Development. 2018;1–13.

For peer review only

37. Eisele G, Vachon H, Lafit G, Kuppens P, Houben M, Myin-Germeys I, et al. The effects of sampling frequency and questionnaire length on perceived burden, compliance, and careless responding in experience sampling data in a student population. Assessment. 2020;1073191120957102.

38. Radez J, Waite P, Chorpita B, Creswell C, Orchard F, Percy R, et al. Using the 11-item Version of the RCADS to Identify Anxiety and Depressive Disorders in Adolescents. Research on child and adolescent psychopathology. 2021;49:1241–57.

39. Bjureberg J, Ljótsson B, Tull MT, Hedman E, Sahlin H, Lundh LG, et al. Development and validation of a brief version of the difficulties in emotion regulation scale: The DERS-16. J Psychopathol Behav Assess. 2016 Jun;38(2):284–96.

40. Gullone E, Taffe J. The Emotion Regulation Questionnaire for Children and Adolescents (ERQ–CA): A psychometric evaluation. Psychological Assessment. 2012;24:409–17.

41. Hinshaw SP, Owens EB, Sami N, Fargeon S. Prospective follow-up of girls with attention-deficit/hyperactivity disorder into adolescence: Evidence for continuing cross-domain impairment. Journal of consulting and clinical psychology. 2006;74(3):489.

42. Owens EB, Hinshaw SP. Childhood conduct problems and young adult outcomes among women with childhood attention-deficit/hyperactivity disorder (ADHD). Journal of abnormal psychology. 2016;125(2):220.

43. Buist KL, Deković M, Meeus WH, van Aken MA. Attachment in adolescence: A social relations model analysis. Journal of Adolescent research. 2004;19(6):826–50.

44. Obsuth I, Murray AL, Knoll M, Ribeaud D, Eisner M. Teacher-student relationships in childhood as a protective factor against adolescent delinquency up to age 17: A propensity score matching approach. Crime & Delinquency. 2021;00111287211014153.

45. Rosenberg M. Rosenberg self-esteem scale (SES). Society and the adolescent self-image. 1965;

46. Gillberg C, Gillberg C, Råstam M, Wentz E. The Asperger Syndrome (and high-functioning autism) Diagnostic Interview (ASDI): a preliminary study of a new structured clinical interview. Autism. 2001;5(1):57–66.

47. Posserud MB, Breivik K, Gillberg C, Lundervold AJ. ASSERT–the autism symptom SElf-ReporT for adolescents and adults: bifactor analysis and validation in a large adolescent population. Research in Developmental Disabilities. 2013;34(12):4495–503.

48. Bondü R, Esser G. Justice and rejection sensitivity in children and adolescents with ADHD symptoms. European Child & Adolescent Psychiatry. 2015;24:185–98.

49. Downey G, Lebolt A, Rincón C, Freitas AL. Rejection sensitivity and children’s interpersonal difficulties. Child development. 1998;69(4):1074–91.

50. Watson D, Clark LA. The PANAS-X: Manual for the positive and negative affect schedule-expanded form. 1999;

51. Gross JJ, John OP. Individual differences in two emotion regulation processes: implications for affect, relationships, and well-being. Journal of personality and social psychology. 2003;85(2):348.

For peer review only

52. Klein A, Clucas J, Krishnakumar A, Ghosh SS, Van Auken W, Thonet B, et al. Remote digital psychiatry for mobile mental health assessment and therapy: MindLogger platform development study. Journal of medical Internet research. 2021;23(11):e22369.

53. Carney CE, Buysse DJ, Ancoli-Israel S, Edinger JD, Krystal AD, Lichstein KL, et al. The consensus sleep diary: standardizing prospective sleep self-monitoring. Sleep. 2012;35(2):287–302.

54. Chan AHY, Horne R, Hankins M, Chisari C. The Medication Adherence Report Scale: A measurement tool for eliciting patients' reports of nonadherence. Br J Clin Pharmacol. 2020 Jul;86(7):1281–8.

55. Pelham Jr WE, Gnagy EM, Greenslade KE, Milich R. Teacher ratings of DSM-III-R symptoms for the disruptive behavior disorders. Journal of the American Academy of Child & Adolescent Psychiatry. 1992;31(2):210–8.

56. Asparouhov T, Hamaker EL, Muthén B. Dynamic structural equation models. Structural Equation Modeling: A Multidisciplinary Journal. 2018;25(3):359–88.

57. Asparouhov T, Muthén B. Comparison of models for the analysis of intensive longitudinal data. Structural Equation Modeling: A Multidisciplinary Journal. 2020;27(2):275–97.

58. McNeish D, Hamaker EL. A primer on two-level dynamic structural equation models for intensive longitudinal data in Mplus. Psychological methods. 2020;25(5):610.

59. Lakens D. Sample size justification. Collabra: Psychology. 2022;8(1):33267.

60. McNeish D. Two-level dynamic structural equation models with small samples. Structural equation modeling: a multidisciplinary journal. 2019;26(6):948–66.

61. Schultzberg M, Muthén B. Number of subjects and time points needed for multilevel time-series analysis: A simulation study of dynamic structural equation modeling. Structural Equation Modeling: A Multidisciplinary Journal. 2018;25(4):495–515.

62. Murray AL, Speyer LG, Brown R, Zhu X, Yang Y, Eisner M, et al. Advancing multi-timeframe developmental research through combining long-term cohort and ecological momentary assessment studies: The Decades-to-minutes (D2M) study [Internet]. PsyArXiv; 2022 [cited 2022 Apr 2]. Available from: https://psyarxiv.com/yqust/

63. Pavarini G, Lorimer J, Manzini A, Goundrey-Smith E, Singh I. Co-producing research with youth: The NeurOx young people's advisory group model. Health Expectations. 2019;22(4):743–51.

64. Breaux R, Langberg JM. Development and refinement of the RELAX intervention, an intervention targeting emotion dysregulation and interpersonal conflict in adolescents with ADHD: Results from a pilot study. Evidence-Based Practice in Child and Adolescent Mental Health. 2020;5(2):147–63.

65. Păsărelu CR, Andersson G, Dobrean A. Attention-deficit/hyperactivity disorder mobile apps: A systematic review. International journal of medical informatics. 2020;138:104133.

66. Melcher J, Patel S, Scheuer L, Hays R, Torous J. Assessing Engagement Features in an Observational Study of Mental Health Apps in College Students. Psychiatry Research. 2022;114470.

For peer review only

67. Baumel A, Muench F, Edan S, Kane JM. Objective user engagement with mental health apps: systematic search and panel-based usage analysis. Journal of medical Internet research. 2019;21(9):e14567.

68. Rintala A, Wampers M, Myin-Germeys I, Viechtbauer W. Momentary predictors of compliance in studies using the experience sampling method. Psychiatry research. 2020;286:112896.

69. Smyth JM, Jones DR, Wen CK, Materia FT, Schneider S, Stone A. Influence of ecological momentary assessment study design features on reported willingness to participate and perceptions of potential research studies: an experimental study. BMJ open. 2021;11(7):e049154.

70. Wrzus C, Neubauer AB. Ecological Momentary Assessment: A Meta-Analysis on Designs, Samples, and Compliance Across Research Fields. Assessment. 2021;10731911211067538.

71. Babinski DE, Welkie J. Feasibility of ecological momentary assessment of negative emotion in girls with ADHD: A pilot study. Psychological reports. 2020;123(4):1027–43.

72. Murray AL, Eisner M, Ribeaud D, Booth T. Validation of a brief measure of aggression for ecological momentary assessment research: the Aggression-ES-A. Assessment. 2020;1073191120976851.

73. Silk JS. Context and dynamics: The new frontier for developmental research on emotion regulation. Developmental psychology. 2019;55(9):2009.

74. Bourchtein E, Langberg JM, Owens JS, Evans SW, Perera RA. Is the positive illusory bias common in young adolescents with ADHD? A fresh look at prevalence and stability using latent profile and transition analyses. Journal of abnormal child psychology. 2017;45(6):1063–75.

Word Count

3,374

Figure Legend

Figure 1: Summary of data collection schedule

For peer review only

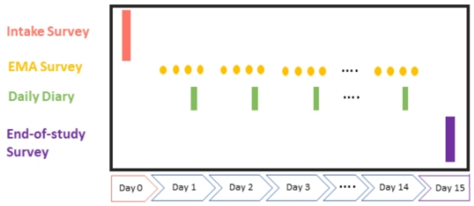

Figure 1: Summary of data collection schedule

338x190mm (96 x 96 DPI)

Supplementary Materials

S1: Description of Measures

Adolescents intake survey

Gender will be measured using an item with options 'male', 'female', 'non-binary', 'gender fluid', 'questioning', 'prefer to self-describe' and 'prefer not to say'.

Date of birth will be collected and used to derive age at time of assessment.

Ethnicity will be measured using a set of items designed to be inclusive of different ethnic identities, with the additional options of "Prefer not to say" and "Not listed, please specify."

Socioeconomic status (SES) will be estimated from respondent's home postcodes and used to derive the (Scottish) index of multiple deprivation [(S)IMD].

Internalising problems, will be measured using an 11-item (6 anxiety items; 5 depression items) self-report *Revised Child Anxiety and Depression* Scale (38) . Previous research has suggest that the scale scores have favourable psychometric properties in adolescents (38)

ADHD strengths will be measured using a newly developed 8-item measure produced by the team in consultation with the youth and parent advisory groups. Items were written for strengths associated with ADHD that included hyperfocus, creativity, energy, interests, and problem-solving. While previous work has identified perceived strengths associated with ADHD (31), no suitable validated measure of ADHD strengths in this age group currently exists. Full details of the scale development and validation will be documented in future publications. This will not be completed by the control sample.

Emotion dysregulation will be measured using *Difficulties in Emotion Regulation Scale* (39),16 item version (DERS-16 Bjureberg et al., 2016). It measures lack of emotional clarity, difficulties in engaging in goal-directed behaviour, impulse control difficulties, limited access to effective emotion regulation strategies, and non-acceptance of emotional responses. Previous research has suggested the scale scores has favourable psychometric properties in adolescents with mental health issues (e.g., Charak et al., 2019).

Emotion regulation strategy use will be measured using the *Emotion Regulation Questionnaire* (42) adapted for children and adolescents (ERQ-CA Gullone & Taffe, 2012). It measures cognitive re-appraisal and suppression in two corresponding subscales of 6 and 4 items respectively. Previous research has suggested that the scale scores have favourable psychometric properties in adolescent samples (43).

Peer problems and friendships will be measured using the Social Relationships Questionnaire (SRQ). This is an 11-item measure of adolescent peer conflict and friendships initially developed for parent-report but adapted for self-report in the current study. Responses are recorded on a 4-point Likert-type scale from *not true* to *very true*. The parent-report version of this measure has been used in prior studies of conduct and peer problems in adolescents with ADHD (44,45).

Attachment security will be measured using the Inventory of Parent and Peer Attachment [IPPA-10;(46)]. This is a 10-item scale measuring respondents' perceptions of trust, communication and alienation in their relationship with a primary caregiver. The scale also yields an overall attachment security score.

The *quality of teacher-student relationships* will be assessed by rating three statements: 'I get along with my teacher'; 'The teacher is fair to me'; and 'The teacher supports me' on a 4-point Likert scale indicating the level of agreement with each

For peer review only

(*completely untrue* to *completely* true). The questions have been used in another longitudinal study and have shown good reliability (alpha = .79) and validity (47).

Self-esteem will be measured using the Rosenberg Self-Esteem questionnaire (48). The scale includes 10 items (5 positively and 5 negatively worded) and an overall self-esteem score can be obtained by (after reverse coding as relevant) summation of the item responses.

Autistic traits will be measured using the 7-item Autism Symptom SElf-ReporT (ASSERT). Derived from a structured diagnostic interview (49), the scale measures social difficulties (4 items) and repetitive behaviour and circumscribed interests (3 items) (50).

Rejection sensitivity will be measured using a 6-item version of the *Child Rejection Sensitivity Questionnaire* (51,52)*.* The scale presents respondents with 6 different scenarios that could result in rejection and for each they are asked to rate how anxious and angry they would feel prior to the outcome and how likely rejection would be. Anxious and anger rejection sensitivity are calculated by multiplication of the likelihood of rejection and corresponding anger/anxious scores for each scenario. Scores for each scenario can be summed to provide overall angry and anxious rejection scores.

Parents intake survey

Respondent gender will be measured using the same item as in the adolescent self-report questionnaire.

Respondent ethnicity will be measured using a set of items designed to be inclusive of different ethnic identities, with the additional options of “Prefer not to say” and “Not listed, please specify.”

Respondent education will be measured by asking respondents “what is the highest degree or level of school you have completed?” A set of items designed to be representative of the educational qualifications throughout the UK are provided with the additional options of “Prefer not to say” and “Not listed, please specify.”

Socioeconomic status will be estimated using SIMD and IMD based on postal code. Parents will also be asked whether their child is eligible for free school meals.

Child’s ADHD Diagnosis and other mental health/neurodevelopmental diagnoses. Parents will be asked for information on who diagnosed their child with ADHD as well as the child’s age at diagnosis. Further, parents will be asked whether their child has any other neurodevelopmental or mental health condition. They can choose from a list or specify other diagnoses in a free text field.

Child’s ADHD medication use. Parents will be asked which ADHD medications their child uses, dosage, and adherence (48). The latter will be measured using the 5-item Medication Adherence Report Scale (MARS, Chan et al., 2020).

Child’s ADHD strengths will be measured using the parent-report version of the above-described ADHD strengths measure (see self-report measures above). They will not be completed by parents of the control sample.

Child’s ADHD, conduct and oppositional symptoms will be measured using the *Disruptive Behavior Disorder Rating Scale* (49)*.* The ADHD subscale includes 9 items measuring symptoms of inattention as well as 9 items measuring symptoms of hyperactivity and impulsivity, scored on a 4-point scale from *not at all* to *very much.* The scale was originally developed under DSM-III-R criteria, however, the symptoms assessed through this measure map on well to criteria outlined in DSM-V (American Psychiatric Association, 2013).

Child’s internalising problems will be measured with a parent-report version of the RCADS-11.

For peer review only

Child's peer conflict and friendships will be measured with the parent-report version of the SRQ.

Ecological momentary assessment measures

ADHD symptoms will be measured using a 3-item momentary symptoms scale developed by the team with reference to DSM criteria. The items are similar to EMA items used in previous EMA ADHD studies (e.g., Pedersen et al., 2020) and cover inattention, hyperactivity, and impulsivity.

Medication use will be measured by asking whether the young person has taken their medication since the last assessment.

Emotions will be measured using abbreviated versions of the Positive Affect Negative Affect Schedule expanded version (PANAS-X;Watson & Clark, 1999). Affect items were selected based on covering a wide range of emotions and being most relevant to ADHD and associated impairments. These will be used to derive indices of emotional lability, inertia, and reactivity within DSEM (53)

Emotion regulation strategies will be measured using an EMA-adapted version of the Emotion Regulation Questionnaire (54). Two items will be used to measure: suppression, and cognitive re-appraisal to experience less negative affect.

Peer interactions will be measured using a brief set of 4 items capturing experiences of *acceptance*, *victimisation, aggression*, and *positive peer interactions.* These items were developed by the team, drawing on previous EMA measures of social experiences (55) and considering common social difficulties among adolescents with ADHD.

Daily diary measures

Medication use will be measured using a two-part item asking whether and when the young person took ADHD medication in the last 24 hours.

Physical activity will be measured using three items asking respondents to report on the amount and intensity of physical activity undertaken in the last 24 hours adapted from an open EMA item pool (56).

Sleep will be measured using an abbreviated version of the Consensus Sleep Diary core items (57).

Adolescents end-of-survey measure

The adolescent end-of-survey questionnaire will be used to re-assess *anxiety and depression* with the RCADS-11. To inform the further development of EMA methodology with adolescents with ADHD, we will ask a brief set of items on their *participation experiences*, including the perceived burden of the EMA and daily diaries, how *enjoyable* the EMA was, how much it changed their *routine*. We will also include an open text field soliciting views on how to improve EMA data collections from a participant's perspective.

Parents end-of-survey measures

The parents end-of survey questionnaire will be used to re-assess their child's emotional and behavioural problems using the RCADS-11 and DBD. It will also measure parents' perspective on the burden of the EMA and daily diary studies. We will also include an open text field soliciting views on how to improve EMA data collections from a participant's perspective.

For peer review only

S2: Adolescents intake online survey

Gender Which of these best describes your gender?

- ☐ Male
- ☐ Female
- ☐ Non-binary
- ☐ Gender fluid
- ☐ Questioning
- ☐ I prefer to self-describe: ___________________________
- ☐ Prefer not to say

Age What is your date of birth (day/month/year)?

____

Ethnicity Which of the following most accurately describes you? Please select all that apply.

- ☐ Bangladeshi
- ☐ Pakistani
- ☐ Indian
- ☐ Chinese
- ☐ Any other Asian background
- ☐ Caribbean
- ☐ African
- ☐ Any other Black, Black British, or Caribbean background
- ☐ White and Black Caribbean
- ☐ White and Black African
- ☐ White and Asian
- ☐ Any other Mixed or multiple ethnic background
- ☐ English, Welsh, Scottish, Northern Irish, or British
- ☐ Irish
- ☐ Gypsy or Irish Traveller
- ☐ Roma
- ☐ Any other White background
- ☐ Arab
- ☐ Prefer not to say
- ☐ Not listed, please specify: ___________________________

Postcode What is your postcode?

____

For peer review only

RCADS-11 Please select the word that shows how often each of these things happened to you over the past two weeks. There are no right or wrong answers.

	Never	Sometimes	Often	Always
I have trouble going to school in the mornings because I feel nervous or afraid	○	○	○	○
I have no energy for things	○	○	○	○
I worry when I go to bed at night	○	○	○	○
I worry about what is going to happen	○	○	○	○
Nothing is much fun anymore	○	○	○	○
All of a sudden I feel really scared for no reason at all	○	○	○	○
I feel worthless	○	○	○	○
I feel sad or empty	○	○	○	○
When I have a problem, my heart beats really fast	○	○	○	○
I am tired a lot	○	○	○	○
I worry I might look foolish	○	○	○	○

For peer review only

Strengths For each item, please select the response that is most true for you.

	Strongly Agree	Agree	Neither Disagree nor Agree	Disagree	Strongly Disagree
I am able to focus really well on the things I am interested in	○	○	○	○	○
I have lots of energy to get things done	○	○	○	○	○
I have tons of ideas	○	○	○	○	○
I am creative	○	○	○	○	○
I have a warm personality	○	○	○	○	○
I am full of enthusiasm	○	○	○	○	○
I am good at coming up with solutions to problems	○	○	○	○	○
I find it easy to develop new interests (e.g., hobbies)	○	○	○	○	○

For peer review only

DERS-16 Please select the response that is most true for you.

	Almost Never	Sometimes	About half the time	Most of the time	Almost always
I have difficulty making sense out of my feelings	○	○	○	○	○
I am confused about how I feel	○	○	○	○	○
When I'm upset, I have difficulty getting work done	○	○	○	○	○
When I'm upset, I become out of control	○	○	○	○	○
When I'm upset, I believe that I will remain that way for a long time	○	○	○	○	○
When I'm upset, I believe that I'll end up feeling very depressed	○	○	○	○	○
When I'm upset, I have difficulty focusing on other things	○	○	○	○	○
When I'm upset, I feel out of control	○	○	○	○	○
When I'm upset, I feel ashamed with myself for feeling that way	○	○	○	○	○
When I'm upset, I feel like I am weak	○	○	○	○	○
When I'm upset, I have difficulty controlling my behaviours	○	○	○	○	○
When I'm upset, I believe there is nothing I can do to make myself feel better	○	○	○	○	○
When I'm upset, I become irritated with myself for feeling that way	○	○	○	○	○
When I'm upset, I start to feel very bad about myself	○	○	○	○	○

For peer review only

When I’m upset, I have difficulty thinking about anything else	○	○	○	○	○
When I’m upset, my emotions feel overwhelming	○	○	○	○	○

For peer review only

ERQ-CA We would now like to ask you some questions about your emotional life. For each item, please select the response that is most true for you.

	Strongly Disagree	Disagree	Half and Half	Agree	Strongly Agree
When I want to feel happier, I think about something different	○	○	○	○	○
I keep my feelings to myself	○	○	○	○	○
When I want to feel less bad (e.g., sad, angry, or worried), I think about something different	○	○	○	○	○
When I am feeling happy, I am careful not to show it	○	○	○	○	○
When I'm worried about something, I make myself think about it in a way that helps me feel better	○	○	○	○	○
I control my feelings by not showing them	○	○	○	○	○
When I want to feel happier about something, I change the way I'm thinking about it	○	○	○	○	○
I control my feelings about things by changing the way I think about them	○	○	○	○	○
When I'm feeling bad (e.g. sad, angry, or worried), I am careful not to show it	○	○	○	○	○
When I want to feel less bad (e.g. sad, angry, or worried) about something, I change the way I'm thinking about it	○	○	○	○	○

For peer review only

SRQ We would like to learn more about your friendships. Please indicate how much each of the following statements applies to you.

	Not True	Sort of True	Mostly True	Very True
You have a "best friend" that you feel close to	○	○	○	○

If "Mostly True" or "Very True" selected display the following questions:

	Not True	Sort of True	Mostly True	Very True
This relationship is positive and beneficial for you (affectionate, supportive, intimate)	○	○	○	○
This relationship is negative for you (high conflict, aggression, betrayal, competitive)	○	○	○	○

Display following questions to all participants:

	Not True	Sort of True	Mostly True	Very True
You have a group of friends at school that you hang around with	○	○	○	○
You have a group of friends at home that you like to play with	○	○	○	○
You have always had problems with friendships	○	○	○	○
You seem to have fewer friends as you get older	○	○	○	○
You have difficulty making new friends	○	○	○	○
You have difficulty keeping the friends that you make	○	○	○	○
Your friend(s) are similar to you in the way they act and the things they like	○	○	○	○

For peer review only

You have problems with other people your age because you tease them	○	○	○	○
You have problems with other people your age because you get teased by them	○	○	○	○
You have difficulty responding appropriately to teasing	○	○	○	○

For peer review only

Caregiver What relationship does the person taking care of you most of the time (i.e. your primary caregiver) have to you?

- ☐ Mother
- ☐ Father
- ☐ Other, please specify: ________________________
- ☐ Prefer not to say

IPPA-10 For each item, please select the response that is most true for you. Please give your answers thinking about the person who takes care of you most of the time (i.e. your primary caregiver).

	Very Untrue	Untrue	Sometimes Untrue	True	Very True
I don't get enough attention from my primary caregiver.	○	○	○	○	○
My primary caregiver accepts me the way I am.	○	○	○	○	○
When my primary caregiver knows something is bothering me, he/she will ask me about it.	○	○	○	○	○
I tell my primary caregiver about my problems and worries.	○	○	○	○	○
I get easily upset with my primary caregiver.	○	○	○	○	○
My primary caregiver respects my feelings.	○	○	○	○	○
I get mad at my primary caregiver.	○	○	○	○	○
My primary caregiver helps me to understand myself better.	○	○	○	○	○
When I talk about my problems with my primary caregiver, I feel ashamed or stupid.	○	○	○	○	○
When I get mad about something, my primary caregiver tries to understand that.	○	○	○	○	○

For peer review only

Teacher For each item, please select the response that is most true for you. Please give your answers thinking about the teacher who knows you best (e.g., your registration/form teacher).

	Completely Untrue	Mostly Untrue	Mostly True	Completely True
I get along with my teacher	○	○	○	○
My teacher is fair to me	○	○	○	○
My teacher supports me	○	○	○	○

For peer review only

Self-Esteem Below is a list of statements dealing with your general feelings about yourself. Please indicate how strongly you agree or disagree with each statement.

	Strongly Agree	Agree	Disagree	Strongly Disagree
On the whole, I am satisfied with myself	○	○	○	○
At times I think I am no good at all	○	○	○	○
I feel that I have a number of good qualities	○	○	○	○
I am able to do things as well as most other people	○	○	○	○
I feel I do not have much to be proud of	○	○	○	○
I certainly feel useless at times	○	○	○	○
I feel that I'm a person of worth, at least on an equal plane with others	○	○	○	○
I wish I could have more respect for myself	○	○	○	○
All in all, I am inclined to feel that I am a failure	○	○	○	○
I take a positive attitude toward myself	○	○	○	○

For peer review only

ASSERT Below is a list of statements dealing with your general feelings about yourself. Please indicate how strongly you agree or disagree with each statement.

	Not True	Somewhat True	Certainly True
Do you find it difficult to socialize with, or to get in touch with people, especially people your own age?	○	○	○
Do you prefer to be alone rather than being together with other people?	○	○	○
Do you have difficulties perceiving social cues?	○	○	○
Do other people tell you that your behavior or your emotional responses are inappropriate or hurtful?	○	○	○
Do you have a strong interest or hobby that absorbs so much of your time that it hampers other activities?	○	○	○
Do you or do other people feel that you have very set routines or that you are very immersed in your own interests?	○	○	○
Do you or do other people feel that you impose your routines or interests on others?	○	○	○

For peer review only

CRSQ Please imagine yourself in each of the following situations described here and select how you would feel in each.

Imagine you had a really bad fight the other day with a friend. Now you have a serious problem and you wish you had your friend to talk to. You decide to wait for your friend after class and talk with him/her. You wonder if your friend will want to talk to you.

1a How NERVOUS would you feel, RIGHT THEN, about whether or not your friend will want to talk to you and listen to your problem?

- ☐ not nervous
- ☐ 2
- ☐ 3
- ☐ 4
- ☐ 5
- ☐ very, very nervous

1b How MAD would you feel, RIGHT THEN, about whether or not your friend will want to talk to you and listen to your problem?

- ☐ not mad
- ☐ 2
- ☐ 3
- ☐ 4
- ☐ 5
- ☐ very, very mad

1c Do you think he/she will want to talk to you and listen to your problem?

- ☐ Yes
- ☐ 2
- ☐ 3
- ☐ 4
- ☐ 5
- ☐ No

Imagine that a famous person is coming to visit your school. Your teacher is going to pick five kids to meet this person. You wonder if she will choose you

2a How NERVOUS would you feel, RIGHT THEN, about whether or not the teacher will choose you?

- ☐ not nervous
- ☐ 2
- ☐ 3
- ☐ 4
- ☐ 5
- ☐ very, very nervous

For peer review only

2b How MAD would you feel, RIGHT THEN, about whether or not the teacher will choose you?

- ☐ not mad
- ☐ 2
- ☐ 3
- ☐ 4
- ☐ 5
- ☐ very, very mad

2c Do you think the teacher will choose YOU to meet the special guest?

- ☐ Yes
- ☐ 2
- ☐ 3
- ☐ 4
- ☐ 5
- ☐ No

Now imagine that you're back in class. Your teacher asks for a volunteer to help plan a party for your class. Lots of kids raise their hands so you wonder if the teacher will choose YOU.

3a How NERVOUS would you feel, RIGHT THEN, about whether or not the teacher will choose you?

- ☐ not nervous
- ☐ 2
- ☐ 3
- ☐ 4
- ☐ 5
- ☐ very, very nervous

3b How MAD would you feel, RIGHT THEN, about whether or not the teacher will choose you?

- ☐ not mad
- ☐ 2
- ☐ 3
- ☐ 4
- ☐ 5
- ☐ very, very mad

3c Do you think the teacher will choose YOU?

- ☐ Yes
- ☐ 2
- ☐ 3
- ☐ 4
- ☐ 5
- ☐ No

For peer review only

Imagine it's Saturday and you're carrying groceries home for your family. It is raining hard and you want to get home FAST. Suddenly, the paper bag you are carrying rips. All your food tumbles to the ground. You look up and see a couple of kids from your class walking quickly. You wonder if they will stop and help you

4a How NERVOUS would you feel, RIGHT THEN, about whether or not those kids will want to stop and help you?

- ☐ not nervous
- ☐ 2
- ☐ 3
- ☐ 4
- ☐ 5
- ☐ very, very nervous

4b How MAD would you feel, RIGHT THEN, about whether or not those kids will want to stop and help you?

- ☐ not mad
- ☐ 2
- ☐ 3
- ☐ 4
- ☐ 5
- ☐ very, very mad

4c Do you think they will offer to help you?

- ☐ Yes
- ☐ 2
- ☐ 3
- ☐ 4
- ☐ 5
- ☐ No

Pretend you have moved and you are going to a different school. In this school, the teacher lets the kids in the class take home a video game to play with on the weekend. Every week so far, you have watched someone else take it home. You decide to ask the teacher if YOU can take home the video game this time. You wonder if she will let you have it

5a How NERVOUS would you feel, RIGHT THEN, about whether or not the teacher will let you take the video game home this time?

- ☐ not nervous
- ☐ 2
- ☐ 3
- ☐ 4
- ☐ 5
- ☐ very, very nervous

For peer review only

5b How MAD would you feel, RIGHT THEN, about whether or not the teacher will let you take the video game home this time?

- ☐ not mad
- ☐ 2
- ☐ 3
- ☐ 4
- ☐ 5
- ☐ very, very mad

5c Do you think the teacher is going to let you take home the video game this time?

- ☐ Yes
- ☐ 2
- ☐ 3
- ☐ 4
- ☐ 5
- ☐ No

Imagine you're back in your classroom, and everyone is splitting up into groups to work on a special project together. You sit there and watch lots of other kids getting picked. As you wait, you wonder if the kids will want you for their group.

6a How NERVOUS would you feel, RIGHT THEN, about whether or not they will choose you?

- ☐ not nervous
- ☐ 2
- ☐ 3
- ☐ 4
- ☐ 5
- ☐ very, very nervous

6b How MAD would you feel, RIGHT THEN, about whether or not they will choose you?

- ☐ not mad
- ☐ 2
- ☐ 3
- ☐ 4
- ☐ 5
- ☐ very, very mad

6c Do you think the the kids in your class will choose you for their group?

- ☐ Yes
- ☐ 2
- ☐ 3
- ☐ 4
- ☐ 5
- ☐ No

For peer review only

S3: EMA and Daily Diary measures

EMA Measures

ADHD symptoms (inattention, hyperactivity, impulsivity)

I couldn't concentrate

Strongly agree	Agree	Neither agree nor disagree	Disagree	Strongly disagree

I acted before thinking

Strongly agree	Agree	Neither agree nor disagree	Disagree	Strongly disagree

I was hyperactive

Strongly agree	Agree	Neither agree nor disagree	Disagree	Strongly disagree

Emotions (PANAS)

In the last 30 minutes I felt…

Excited

Very slightly or not at all	A little	Moderately	Quite a bit	Extremely

Upset

Very slightly or not at all	A little	Moderately	Quite a bit	Extremely

Determined

Very slightly or not at all	A little	Moderately	Quite a bit	Extremely

Enthusiastic

Very slightly or not at all	A little	Moderately	Quite a bit	Extremely

Ashamed

Very slightly or not at all	A little	Moderately	Quite a bit	Extremely

Nervous

Very slightly or not at all	A little	Moderately	Quite a bit	Extremely

For peer review only

Emotion regulation strategy use

In the last 30 minutes, I…

Kept my emotions to myself

Strongly Disagree	Disagree	Neither agree nor disagree	Agree	Strongly Agree

When I wanted to feel less negative emotion (such as sadness or anger), I changed what I was thinking about

Strongly Disagree	Disagree	Neither agree nor disagree	Agree	Strongly Agree

Peer interactions

In relation to my peers (other adolescents my age) in the last 30 minutes, I…

Felt accepted

Strongly Disagree	Disagree	Neither agree nor disagree	Agree	Strongly Agree

Was teased

Strongly Disagree	Disagree	Neither agree nor disagree	Agree	Strongly Agree

Got into an argument

Strongly Disagree	Disagree	Neither agree nor disagree	Agree	Strongly Agree

I enjoyed interacting (online or in person) with others my age.

Strongly Disagree	Disagree	Neither agree nor disagree	Agree	Strongly Agree

For peer review only

Daily Diary Measures

Physical activity

How many minutes did you do vigorous activities including intensive sports or exercise (such as running or fast cycling) or intensive physical work (such as heavy lifting or digging)?

How many minutes did you do moderate activities (activities that make you breathe somewhat harder than usual such as playing tennis, bicycling, carrying light loads)?

How many minutes did you do light activities (activities that may not make you breathe somewhat harder than usual such as walking, climbing stairs, routine household chores, etc.)?

Medication use

Have you taken ADHD medication in the last 24 hours?

Sleep (consensus sleep scale core items)

What time did you get into bed last night?

What time did you try to go to sleep?

How long did it take you to fall asleep (in minutes)?

How many times did you wake up, not counting your final awakening?

In total, how long did these awakenings last (in minutes)?

What time was your final awakening?

For peer review only

How would you rate quality of your sleep?

Very poor	Poor	Fair	Good	Very Good

Comments (if applicable)

For peer review only

S4: Adolescents end-of-study survey

RCADS-11 Please select the word that shows how often each of these things happened to you over the past two weeks. There are no right or wrong answers.

	Never	Sometimes	Often	Always
I have trouble going to school in the mornings because I feel nervous or afraid	○	○	○	○
I have no energy for things	○	○	○	○
I worry when I go to bed at night	○	○	○	○
I worry about what is going to happen	○	○	○	○
Nothing is much fun anymore	○	○	○	○
All of a sudden I feel really scared for no reason at all	○	○	○	○
I feel worthless	○	○	○	○
I feel sad or empty	○	○	○	○
When I have a problem, my heart beats really fast	○	○	○	○
I am tired a lot	○	○	○	○
I worry I might look foolish	○	○	○	○

For peer review only

EMA feedback We would love to know how taking part in the smart-phone based surveys was for you. Please select the response that is most true for you thinking about your experiences of completing the smartphone-based surveys.

	Almost never	Sometimes	About half the time	Most of the time	Almost always
I found doing the smartphone surveys fun	○	○	○	○	○
I found doing the smartphone surveys hard work	○	○	○	○	○
Doing the smartphone surveys disrupted my routine	○	○	○	○	○

Suggestions Do you have thoughts or suggestions on how the smartphone surveys could be improved?

____

For peer review only

S5: Parents online intake survey

Gender Which of these best describes your gender?

- ☐ Male
- ☐ Female
- ☐ Non-binary
- ☐ Gender fluid
- ☐ Questioning
- ☐ I prefer to self-describe: ___________________________
- ☐ Prefer not to say

Age What is your date of birth (day/month/year)?

____

Ethnicity Which of the following most accurately describes you? Please select all that apply.

- ☐ Bangladeshi
- ☐ Pakistani
- ☐ Indian
- ☐ Chinese
- ☐ Any other Asian background
- ☐ Caribbean
- ☐ African
- ☐ Any other Black, Black British, or Caribbean background
- ☐ White and Black Caribbean
- ☐ White and Black African
- ☐ White and Asian
- ☐ Any other Mixed or multiple ethnic background
- ☐ English, Welsh, Scottish, Northern Irish, or British
- ☐ Irish
- ☐ Gypsy or Irish Traveller
- ☐ Roma
- ☐ Any other White background
- ☐ Arab
- ☐ Prefer not to say
- ☐ Not listed, please specify: ___________________________

Education What is the highest degree or level of school you have completed?

- ☐ Less than a high school diploma
- ☐ High school or equivalent (e.g. Scottish National level 5, GCSE)
- ☐ College/Sixth form (e.g. Scottish Highers/Higher National Certificate, Advanced Highers/Higher National Diploma, BTEC, NVQ, AS/A-levels)
- ☐ Further education college (e.g. HNC, HND)
- ☐ Vocational (e.g. apprenticeship, higher apprenticeship)
- ☐ Bachelor's degree (e.g. BA, BSc)
- ☐ Master's degree (e.g. MA, MSc, MEd)
- ☐ Doctorate or professional degree (e.g. MD, DDS, PhD)
- ☐ Prefer not to say
- ☐ Not listed, please specify: ___________________________

For peer review only

Postcode What is your postcode?

____

Meals Is your child eligible for free school meals?

- ☐ Yes
- ☐ No

Diagnosis Source Who diagnosed your child with ADHD?

- ☐ General Practitioner
- ☐ Pediatrician
- ☐ Psychiatrist
- ☐ Psychologist
- ☐ Neurologist
- ☐ Other, please specify: ______________________________

Diagnosis Age How old was your child when they were diagnosed with ADHD?

Diagnoses Other Does your child have any other mental health or neurodevelopmental diagnoses?

- ☐ Yes
- ☐ No

If yes, display this question:

Diagnoses List Which other diagnoses does your child have? Please select all that apply.

- ☐ Autism Spectrum Disorder
- ☐ Foetal Alcohol Syndrome
- ☐ Intellectual Disability
- ☐ Anxiety Disorder
- ☐ Depression
- ☐ Conduct Disorder
- ☐ Other, please specify: ______________________________

For peer review only

Medication Is your child currently taking any ADHD medication?

- ☐ Yes
- ☐ No

If yes, display this question:

Medication Details Which medicine is your child taking and what dosage?

- ☐ Medicine: ___________________________
- ☐ Dosage (e.g., XX mg, 2x a day): ___________________________

Medication Adherence Please select the response that is most true for your child.

	Always	Often	Sometimes	Rarely	Never
My child forgets to take their medication	○	○	○	○	○
My child changes the dosage of their medication	○	○	○	○	○
My child stops taking their medication for a while	○	○	○	○	○
My child decides to skip one of their medication dosages	○	○	○	○	○
My child uses their medication less than is prescribed	○	○	○	○	○

For peer review only

Strengths Please select the response that is most true for your child.

	Strongly Agree	Agree	Neither Disagree nor Agree	Disagree	Strongly Disagree
My child is able to focus really well on the things they are interested in	○	○	○	○	○
My child has lots of energy to get things done	○	○	○	○	○
My child has tons of ideas	○	○	○	○	○
My child is creative	○	○	○	○	○
My child has a warm personality	○	○	○	○	○
My child is full of enthusiasm	○	○	○	○	○
My child is good at coming up with solutions to problems	○	○	○	○	○
My child finds it easy to develop new interests (e.g., hobbies)	○	○	○	○	○

For peer review only

DBD Please select the option that best describes your child. Please give your answers on the basis of your child's behaviour over the last six months

	Not at All	Just a Little	Pretty Much	Very Much	Don't Know or Not Applicable
Often interrupts or intrudes on others (e.g., butts into conversations or games)	○	○	○	○	○
Has run away from home overnight at least twice while living in parental or parental surrogate home (or once without returning for a lengthy period)	○	○	○	○	○
Often argues with adults	○	○	○	○	○
Often lies to obtain goods or favors or to avoid obligations (i.e., "cons" others)	○	○	○	○	○
Often initiates physical fights with other members of his or her household	○	○	○	○	○
Has been physically cruel to people	○	○	○	○	○
Often talks excessively	○	○	○	○	○
Has stolen items of nontrivial value without confronting a victim (e.g., shoplifting, but without breaking and entering; forgery)	○	○	○	○	○
Is often easily distracted by extraneous stimuli	○	○	○	○	○

For peer review only

	Not at All	Just a Little	Pretty Much	Very Much	Don't Know or Not Applicable
Often engages in physically dangerous activities without considering possible consequences (not for the purpose of thrill-seeking), e.g., runs into street without looking	○	○	○	○	○
Often truant from school, beginning before age 13 years	○	○	○	○	○
Often fidgets with hands or feet or squirms in seat	○	○	○	○	○
Is often spiteful or vindictive	○	○	○	○	○
Often swears or uses obscene language	○	○	○	○	○
Often blames others for his or her mistakes or misbehavior	○	○	○	○	○
Has deliberately destroyed others' property (other than by fire setting)	○	○	○	○	○
Often actively defies or refuses to comply with adults' requests or rules	○	○	○	○	○
Often does not seem to listen when spoken to directly	○	○	○	○	○
Often blurts out answers before questions have been completed	○	○	○	○	○
Often initiates physical fights with others who do not live in his or her household (e.g., peers at school or in the neighborhood)	○	○	○	○	○
Often shifts from one uncompleted activity to another	○	○	○	○	○
Often has difficulty playing or engaging in leisure activities quietly	○	○	○	○	○

For peer review only

	Not at All	Just a Little	Pretty Much	Very Much	Don't Know or Not Applicable
Often fails to give close attention to details or makes careless mistakes in schoolwork, work, or other activities					
Is often angry and resentful					
Often leaves seat in classroom or in other situations in which remaining seated is expected					
Is often touchy or easily annoyed by others					
Often does not follow through on instructions and fails to finish schoolwork, chores, or duties in the workplace (not due to oppositional behavior or failure to understand instructions)					
Often loses temper					
Often has difficulty sustaining attention in tasks or play activities					
Often has difficulty awaiting turn					
Has forced someone into sexual activity					
Often bullies, threatens, or intimidates others					
Is often "on the go" or often acts as if "driven by a motor"					
Often loses things necessary for tasks or activities (e.g., toys, school assignments, pencils, books, or tools)					

For peer review only

Often runs about or climbs excessively in situations in which it is inappropriate (in adolescents or adults, may be limited to subjective feelings of restlessness)	○	○	○	○	○
Has been physically cruel to animals	○	○	○	○	○
Often avoids, dislikes, or is reluctant to engage in tasks that require sustained mental effort (such as schoolwork or homework)	○	○	○	○	○
Often stays out at night despite parental prohibitions, beginning before age 13 years	○	○	○	○	○
Often deliberately annoys people	○	○	○	○	○
Has stolen while confronting a victim (e.g., mugging, purse snatching, extortion, armed robbery)	○	○	○	○	○
Has deliberately engaged in fire setting with the intention of causing serious damage	○	○	○	○	○
Often has difficulty organizing tasks and activities	○	○	○	○	○
Has broken into someone else's house, building, or car	○	○	○	○	○
Is often forgetful in daily activities	○	○	○	○	○
Has used a weapon that can cause serious physical harm to others (e.g., a bat, brick, broken bottle, knife, gun)	○	○	○	○	○

For peer review only

RCADS-11 Please select the option that best describes your child. Please give your answers on the basis of your child's behaviour over the last six months.

	Never	Sometimes	Often	Always
My child has trouble going to school in the mornings because of feeling nervous or afraid	○	○	○	○
My child has no energy for things	○	○	○	○
My child worries when in bed at night	○	○	○	○
My child worries about what is going to happen	○	○	○	○
Nothing is much fun for my child anymore	○	○	○	○
All of a sudden my child will feel really scared for no reason at all	○	○	○	○
My child feels worthless	○	○	○	○
My child feels sad or empty	○	○	○	○
When my child has a problem, his/her heart beats really fast	○	○	○	○
My child is tired a lot	○	○	○	○
My child worries about looking foolish	○	○	○	○

For peer review only

SRQ We would like to learn more about your child's peer relationships. Please indicate how much each of the following statements applies to your child.

	Not True	Sort of True	Mostly True	Very True
My child has a "best friend" that he/she feels close to	○	○	○	○

If "Mostly True" or "Very True" selected display the following questions:

	Not True	Sort of True	Mostly True	Very True
This relationship is positive and beneficial for my child (affectionate, supportive, intimate)	○	○	○	○
This relationship is negative for my child (high conflict, aggression, betrayal, competitive)	○	○	○	○

Display following questions to all participants:

	Not True	Sort of True	Mostly True	Very True
My child has a group of friends at school that he/she hangs around with	○	○	○	○
My child has a group of friends at home that he/she likes to play with	○	○	○	○
My child has always had problems with peer relationships	○	○	○	○
My child seems to have fewer friends as he/she grows older	○	○	○	○
My child has difficulty making new friends	○	○	○	○
My child has difficulty keeping the friends that he/she makes	○	○	○	○

For peer review only

My child's friend(s) are similar to him/her in the way they act and the things they like	○	○	○	○
My child has problems with other children because he/she teases them	○	○	○	○
My child has problems with other children because he/she gets teased by them	○	○	○	○
My child has difficulty responding appropriately to teasing	○	○	○	○

For peer review only

S6: Parents end-of-study survey

DBD Please select the option that best describes your child. Please give your answers on the basis of your child's behaviour over the last six months

	Not at All	Just a Little	Pretty Much	Very Much	Don't Know or Not Applicable
Often interrupts or intrudes on others (e.g., butts into conversations or games)	○	○	○	○	○
Has run away from home overnight at least twice while living in parental or parental surrogate home (or once without returning for a lengthy period)	○	○	○	○	○
Often argues with adults	○	○	○	○	○
Often lies to obtain goods or favors or to avoid obligations (i.e., "cons" others)	○	○	○	○	○
Often initiates physical fights with other members of his or her household	○	○	○	○	○
Has been physically cruel to people	○	○	○	○	○
Often talks excessively	○	○	○	○	○
Has stolen items of nontrivial value without confronting a victim (e.g., shoplifting, but without breaking and entering; forgery)	○	○	○	○	○
Is often easily distracted by extraneous stimuli	○	○	○	○	○

For peer review only

	Not at All	Just a Little	Pretty Much	Very Much	Don't Know or Not Applicable
Often engages in physically dangerous activities without considering possible consequences (not for the purpose of thrill-seeking), e.g., runs into street without looking	○	○	○	○	○
Often truant from school, beginning before age 13 years	○	○	○	○	○
Often fidgets with hands or feet or squirms in seat	○	○	○	○	○
Is often spiteful or vindictive	○	○	○	○	○
Often swears or uses obscene language	○	○	○	○	○
Often blames others for his or her mistakes or misbehavior	○	○	○	○	○
Has deliberately destroyed others' property (other than by fire setting)	○	○	○	○	○
Often actively defies or refuses to comply with adults' requests or rules	○	○	○	○	○
Often does not seem to listen when spoken to directly	○	○	○	○	○
Often blurts out answers before questions have been completed	○	○	○	○	○
Often initiates physical fights with others who do not live in his or her household (e.g., peers at school or in the neighborhood)	○	○	○	○	○
Often shifts from one uncompleted activity to another	○	○	○	○	○
Often has difficulty playing or engaging in leisure activities quietly	○	○	○	○	○

For peer review only

	Not at All	Just a Little	Pretty Much	Very Much	Don't Know or Not Applicable
Often fails to give close attention to details or makes careless mistakes in schoolwork, work, or other activities	○	○	○	○	○
Is often angry and resentful	○	○	○	○	○
Often leaves seat in classroom or in other situations in which remaining seated is expected	○	○	○	○	○
Is often touchy or easily annoyed by others	○	○	○	○	○
Often does not follow through on instructions and fails to finish schoolwork, chores, or duties in the workplace (not due to oppositional behavior or failure to understand instructions)	○	○	○	○	○
Often loses temper	○	○	○	○	○
Often has difficulty sustaining attention in tasks or play activities	○	○	○	○	○
Often has difficulty awaiting turn	○	○	○	○	○
Has forced someone into sexual activity	○	○	○	○	○
Often bullies, threatens, or intimidates others	○	○	○	○	○
Is often "on the go" or often acts as if "driven by a motor"	○	○	○	○	○
Often loses things necessary for tasks or activities (e.g., toys, school assignments, pencils, books, or tools)	○	○	○	○	○

For peer review only

Often runs about or climbs excessively in situations in which it is inappropriate (in adolescents or adults, may be limited to subjective feelings of restlessness)	○	○	○	○	○
Has been physically cruel to animals	○	○	○	○	○
Often avoids, dislikes, or is reluctant to engage in tasks that require sustained mental effort (such as schoolwork or homework)	○	○	○	○	○
Often stays out at night despite parental prohibitions, beginning before age 13 years	○	○	○	○	○
Often deliberately annoys people	○	○	○	○	○
Has stolen while confronting a victim (e.g., mugging, purse snatching, extortion, armed robbery)	○	○	○	○	○
Has deliberately engaged in fire setting with the intention of causing serious damage	○	○	○	○	○
Often has difficulty organizing tasks and activities	○	○	○	○	○
Has broken into someone else's house, building, or car	○	○	○	○	○
Is often forgetful in daily activities	○	○	○	○	○
Has used a weapon that can cause serious physical harm to others (e.g., a bat, brick, broken bottle, knife, gun)	○	○	○	○	○

For peer review only

RCADS-11 Please select the option that best describes your child. Please give your answers on the basis of your child's behaviour over the last six months.

	Never	Sometimes	Often	Always
My child has trouble going to school in the mornings because of feeling nervous or afraid	○	○	○	○
My child has no energy for things	○	○	○	○
My child worries when in bed at night	○	○	○	○
My child worries about what is going to happen	○	○	○	○
Nothing is much fun for my child anymore	○	○	○	○
All of a sudden my child will feel really scared for no reason at all	○	○	○	○
My child feels worthless	○	○	○	○
My child feels sad or empty	○	○	○	○
When my child has a problem, his/her heart beats really fast	○	○	○	○
My child is tired a lot	○	○	○	○
My child worries about looking foolish	○	○	○	○

For peer review only

EMA feedback We would appreciate getting your feedback on the smart-phone based surveys that your child took part in.

	Almost never	Sometimes	About half the time	Most of the time	All of the time
My child found doing the smartphone surveys fun	○	○	○	○	○
I would allow my child to participate in a study like this involving smartphone surveys again	○	○	○	○	○

Suggestions Do you have thoughts or suggestions on how the smartphone surveys could be improved?

____

For peer review only
