# Supplementary material for: Illuminating the daily life experiences of adolescents with and without ADHD: protocol for an ecological momentary assessment study
Source: BMJ Open. 2023 Sep 29;13(9):e077222. doi: 10.1136/bmjopen-2023-077222 (PMC10546102; doi:10.1136/bmjopen-2023-077222)
Supplement: Reviewer comments [file bmjopen-2023-077222.reviewer_comments.pdf]

## PEER REVIEW HISTORY

BMJ Open publishes all reviews undertaken for accepted manuscripts. Reviewers are asked to complete a checklist review form (**http://bmjopen.bmj.com/site/about/resources/checklist.pdf**) and are provided with free text boxes to elaborate on their assessment. These free text comments are reproduced below.

## ARTICLE DETAILS

| | |
|---|---|
| **TITLE (PROVISIONAL)** | Illuminating the daily life experiences of adolescents with and without ADHD: Protocol for an ecological momentary assessment study |
| **AUTHORS** | Murray, Aja; Speyer, Lydia; Thye, Melissa; Stewart, Tracy; Obsuth, Ingrid; Kane, Jennifer; Whyte, Katie; Devaney, John; Rohde, Luis; Ushakova, Anastasia; Rhodes, Sinead |

This article was not externally reviewed at BMJ Open. Protocols that have been independently assessed prior to submission to BMJ Open are usually fast-tracked to publication on the grounds that further substantial changes will not be possible. This independent assessment will usually be external, independent review for both a substantial grant award from a non-commercial or government funder and ethics approval.
